# Electrochemical Insight into the Copper Redox Chemistry and H_2_O_2_ and O_2_ Reducing Capability of Two AA10 Lytic Polysaccharide Monooxygenases

**DOI:** 10.1021/acselectrochem.5c00266

**Published:** 2026-01-15

**Authors:** Ella K. Reid, Connor G. Miles, Henry O. Lloyd-Laney, Alison K. Nairn, Jessie Branch, Nicholas Garland, Nicholas D. J. Yates, Alex Ascham, Paul H. Walton, Glyn Hemsworth, Alison Parkin

**Affiliations:** † Department of Chemistry, 8748 University of York, Heslington, York YO10 5DD, U.K.; ‡ Department of Computer Science, 6396 University of Oxford, Oxford OX1 3QG, U.K.; § Astbury Centre for Structural Molecular Biology and School of Molecular and Cellular Biology, Faculty of Biological Sciences, 4468 University of Leeds, Leeds LS2 9JT, U.K.

**Keywords:** lytic polysaccharide monooxygenases, enzyme electrochemistry, type-II copper protein film voltammetry, bioelectrochemical assay

## Abstract

Lytic polysaccharide monooxygenases ([L]­PMOs) are copper-containing enzymes that catalyse cleavage of the glycosidic bond, a process central to microbial biomass degradation. Here, we describe electrochemical methods used to investigate the Cu^2+/1+^ redox chemistry and the polysaccharide-free catalytic activity of two AA10 LPMOs: *Cj*AA10B from *Cellvibrio japonicus* and *Cf*AA10 from *Cellulomonas fimi*. Immobilisation of these enzymes on the surface of a graphite electrode allows for direct electrochemical measurements of Cu^2+/1+^ redox cycling as well as the ability of both LPMOs to reduce H_2_O_2_ vs O_2_. These measurements can be advantageous when compared to biological dye assays as they provide direct kinetic measurements and allow for investigation over a wider range of environmental conditions. Values of *k*_cat_ and *K*_M_- are reported for H_2_O_2_ and O_2_ reduction by *Cj*AA10B and *Cf*AA10 from pH 5–7, with *Cf*AA10 consistently outperforming *Cj*AA10B. Both enzymes perform faster catalysis with H_2_O_2_ but when comparing the affinity-coupled specificity constant (*k*_cat_/*K*_M_), the LPMOs perform similarly with both H_2_O_2_ and O_2_, suggesting both substrates are viable. We also note an increase in redox signals as pH is decreased that correlates with EPR data suggesting a second species is formed <pH 5, postulated to occur due to the protonation of a glutamate residue (p*K*_a_ ∼ 4.6). The increase in signal size with decreasing pH that is seen for the non-catalytic Cu^2+/1+^ transition is interpreted in light of an increasing proportion of electroactive species at low pH; such a change in activity with pH is notably not observed in the presence of substrate (H_2_O_2_ or O_2_). This suggests that substrate binding modulates the active site, disrupting the effect of protonation. These findings establish electrochemistry as a powerful tool for probing LPMO activity.

## Graphical abstract

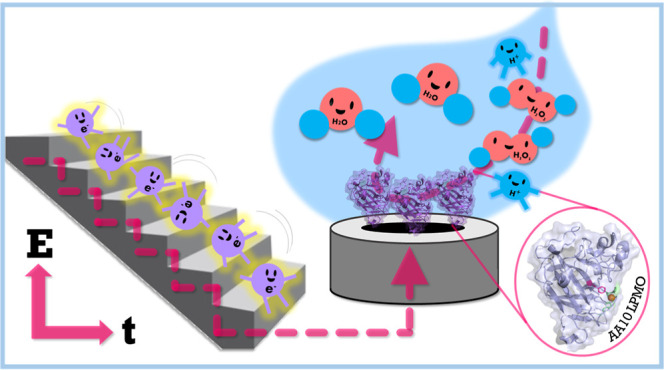

## Introduction

Lytic polysaccharide monooxygenases ([L]­PMOs) were first described as copper-containing enzymes in 2011.^1,2^ LPMOs are capable of degrading recalcitrant carbohydrate substrates by facilitating cleavage of the glycosidic bond at the C1/C4 position, an example mechanism of activation at the C4 position is shown in Figure 1 in which the LPMO inserts an oxygen atom into a C–H bond, initiating spontaneous glycosidic bond lysis. Along with applications in biofuel production due to their polysaccharide-degrading chemistries,^3,4^ LPMOs have also been recognized as microbial virulence factors.^5–7^ It is therefore useful to identify new techniques which enable LPMO reactivity to be related to enzyme sequence and structure. Both molecular oxygen, O_2_, and hydrogen peroxide, H_2_O_2_, have been found to act as the source of oxygen in the glycosidic bond cleavage reaction. When using O_2_ as a co-substrate the LPMO is acting as a classic oxidoreductase enzyme, requiring an exogenous electron donor, conversely, the use of H_2_O_2_ as a co-substrate only requires reductive activation of the LPMO rather than a continual electron supply (Figure S1). Therefore, this paper reports on how electrochemical methods can be used to quantitatively probe the oxidoreductase capabilities of two LPMO enzymes.

**Figure 1:**
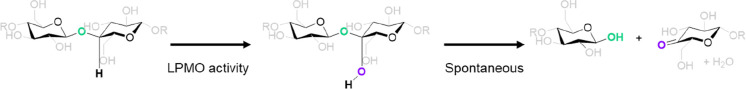
Example LPMO mechanism involving oxygen insertion to the C–H bond at the glycosidic linkage followed by spontaneous bond lysis.

LPMO enzymes are classified into different “auxiliary activity” (AA) classes of carbohydrate activating enzymes, on the basis of their amino-acid sequence, in the carbohydrate active enzymes (CAZy) database (http://www.cazy.org/).^8^ The work described here focuses on AA10 enzymes, a large family of LPMOs originating from a variety of organisms, however the majority of characterized AA10s have been derived from bacteria.^4,8^ Crystal structure data has shown that the active site copper coordinating “histidine-brace” is conserved across LPMOs,^9^ a copper-binding motif typically composed of two histidines and the amino terminus of His1.^10^ AA10s can be further subdivided based on the residue in the axial position to the copper; whether this is phenylalanine or tyrosine. Given that these residues have been implicated in the redox control of LPMOs,^11^ we have chosen to explore the chemistries of one LPMO from each of these sub-categories; *Cf*AA10 and *Cj*AA10BΔCBM (Figures 4A and S2), both of which have been proven active on cellulose in the presence of either chemical reducing agents or a small c-type cytochrome.^12^

Electron paramagnetic (EPR) spectroscopy has been used ubiquitously in the characterisation of the structure and mechanism of LPMOs, showing a type-II active site copper that transitions from an as-isolated, EPR-visible Cu^2+^ state to an EPR-silent Cu^1+^ state upon reaction with chemical reducing agents.^1,13–16^ Reductive activation to generate the Cu^1+^ state is necessary for LPMO catalysis of glycosidic bond cleavage.^2,13,16^ A substantive body of literature has comprehensively established that post reduction to generate the Cu^1+^ oxidation state, in vitro carbohydrate degradation assays require H_2_O_2_ or O_2_ as a co-substrate.^17–20^

It is difficult to determine the in vivo reaction mechanism of LPMOs because these are secreted proteins that operate in concert with other CAZymes to degrade crystalline substrates in extracellular environments such as leaf mulch. LPMOs were originally thought to act with O_2_ as their co-substrate with concomitant Cu^2+/1+^ oxidation state cycling being driven by an external electron donor.^16,21^ Putative LPMO electron donor partners have been identified in both fungal and bacterial systems,^22^ and hole-hopping pathways have been identified in LPMO structures which could underpin such redox reactivity.^23–25^ However, since their discovery, it has been shown that LPMOs can operate with H_2_O_2_ in place of O_2_.^17^ Determining if LPMOs have evolved to operate with H_2_O_2_ as their optimized co-substrate, or if the H_2_O_2_ is a “catalytic shunt” that is only used in vitro (from an oxidation state perspective, H_2_O_2_ is equivalent to O_2_ + 2H^+^ + 2e^–^) is further complicated because mixing together chemical reducing agents and O_2_ can sometimes generate H_2_O_2_ in situ. Therefore, controversy still surrounds the true nature of the in vivo, co-substrate.

Mass spectrometry assays can be used to study glycosidic bond cleavage in polymeric carbohydrate substrates, however, these assays require incubation of the enzymes and substrates for hours, making it challenging to achieve a constant supply of reducing equivalents.^26–33^ Several in vitro dye assays have also been developed to address the mechanistic uncertainties. The colorimetric oxidation of 2,6-dimethoxyphenol (2,6-DMP) with simultaneous LPMO-catalysed reduction of H_2_O_2_ to H_2_O is a useful tool for rapid comparative screening of LPMOs and variants.^26^ There is, however, no well-established method in the literature for looking directly at the O_2_ reduction activity of an LPMO. Amplex Red hydrogen peroxide reductase assays provide an indirect measurement of the rate of O_2_-reduction by LPMOs, but these assays actually monitor H_2_O_2_ being turned over by a second enzyme and thus provide no direct insight into the chemistries of the LPMO in the presence of O_2_.^34,35^

Outside of mass spectrometry and dye assays, electrochemistry has been used as a tool to investigate the copper chemistry of LPMOs. The Karantonis group used a Nafion membrane to stabilise AA9 enzymes on the electrode surface,^36–38^ allowing midpoint potentials and kinetic rate constants for AA9 Cu^2+/1+^ transitions to be determined (Table 1); however, the lack of permeability of Nafion to O_2_ prevents the measurement of any catalytic electron transfer.^39^ More recently, Moura and co-workers showed that direct immobilisation of AA10 LPMO samples on a graphite electrode removed the requirement for a Nafion membrane.^11^ Using this method, reduction potentials and the kinetics of copper electron transfer for both wild-type and active-site variant LPMOs were determined, and it was shown that the mutation of active site neighbouring residues, particularly the F219A amino acid exchange, tunes both the reduction potential and the internal electron transfer rate of the enzyme.^11^ Cytochrome p450s represent another family of monooxygenases that have been widely and successfully studied electrochemically, despite facing similar difficulties in productive immobilization and complications from their requirement of oxygen as a co-substrate.^40,41^

**Table 1:** Comparison of all Electrochemical LPMO Characterisations from the literature^11,36,38^ and the Results Reported in This Study

LPMO	family	pH	temperature/ °C	reduction potential/mV vs SHE	Cu^2+/1+^ rate constant/s^–1^	technique of measurement
*Mt*LPMO9 ^34^	AA9	5	30	∼321	4.6	FTACV
*Fo*LPMO9 ^34^	AA9	5	29	∼276	-	FTACV
*Pc*LPMO9D^36^	AA9	5	50	∼351	-	FTACV
*Nc*LPMO9C^36^	AA9	5	50	∼412	-	FTACV
*Sc*LPMO10C-WT^37^	AA10	7	30	∼190	0.48	DCV
*Sc*LPMO10C-A142G^37^	AA10	7	30	∼186	0.17	DCV
*Sc*LPMO10C–F219Y^37^	AA10	7	30	∼201	0.47	DCV
*Sc*LPMO10C–F219A^37^	AA10	7	30	∼202	1.06	DCV
*Cj*AA10BΔCBM	AA10	5	35	240 ± 15	0.35 ± 0.2	SWV
*Cf*AA10	AA10	5	35	290 ± 25	0.52 ± 0.3	SWV

Our work presented here builds on previous electrochemical and biological dye assays to provide a toolkit for the investigation of not only the redox chemistry of the copper centre of two AA10 LPMOs but also a new method for assaying the electroactivity of these enzymes in the presence of H_2_O_2_/O_2_, contrasted with the equivalent biological dye assays in Figure 2. By immobilising the LPMOs onto a rotating disk working electrode we are able to “wire” the enzyme to the electrode, allowing for direct measurements of the redox activity in both the presence and absence of substrate. The rotation of the electrode allows us to control the hydrodynamic flux of the buffer solution and ensure that the measured catalytic rates reflect the inherent maximum turnover of the enzyme, rather than being limited by soluble substrate diffusion.^42^ The protein-film configuration of our experiments also makes it relatively trivial to vary the environmental conditions, allowing us to examine the effect of pH on LPMO reactivity. We find that increasing the pH above 6.0 causes a distinct, reversible loss of non-catalytic Cu^2+/1+^ electron-transfer current, yet the same reduction in activity was not observed for either O_2_ or H_2_O_2_ electroreduction. We combine these electrochemical insights with complementary X-ray crystallography data and EPR experiments to demonstrate how electrocatalysis can be integrated with other techniques as part of a wider LPMO biochemical toolkit.

**Figure 2:**
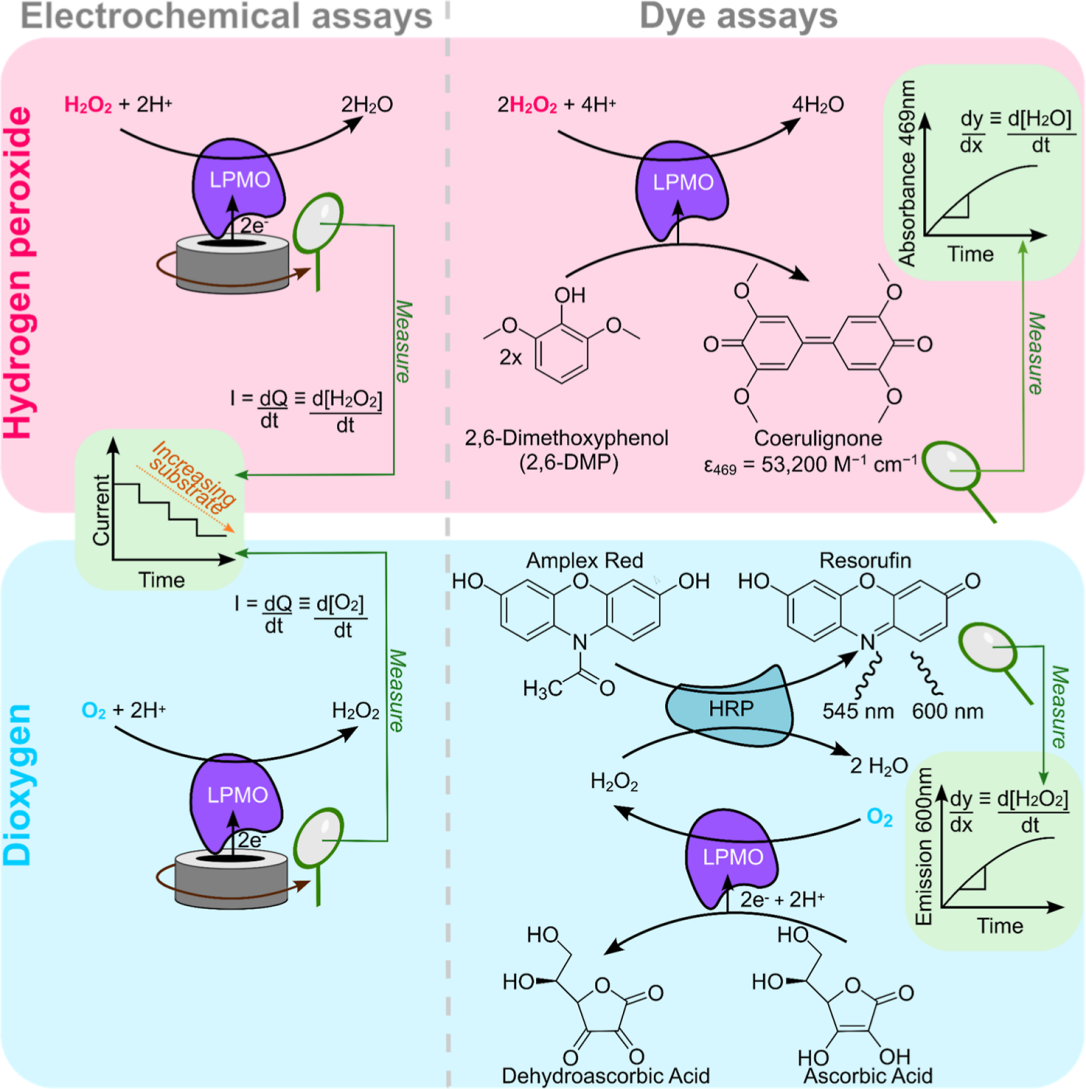
Summary of electrochemical assays contrasted with dye assays. (A) Electrochemical LPMO H_2_O_2_ reduction assay. (B) Solution 2,6-DMP LPMO peroxygenase assay. (C) Electrochemical LPMO O_2_ reduction assay, (D) Solution Amplex Red LPMO H_2_O_2_ production assay.

## Experimental Section

### Protein Production and Biochemical Assays

Lytic polysaccharide monooxygenase protein production and biochemical assay methods were carried out using minor modifications of previously published procedures. Full details are provided in the Supporting Information.

### X-ray Crystallography of CjAA10B

Purified *Cj*AA10BΔCBM_C‑His_ was concentrated to 10 mg/mL and screened for crystallization using four commercially available crystallization screens: JCSG Core I–IV (QIAGEN). Crystals formed in 0.1 M Tris pH 8.5 and 1 M diammonium hydrogen phosphate. Crystals were harvested directly from this condition without further optimisation and cryo-protected in mother liquor supplemented with 20% (v/v) ethylene glycol for 30 s, before being cryocooled in liquid N_2_. Data was collected on beamline i04 at Diamond Light Source with a wavelength of 0.979 Å. Diffraction data were autoprocessed using the autoPROC software package.^43^ Autoprocessed data were input into CCP4i2 ^44^ and cut to 1.9 Å (Data were originally collected to 1.75 Å) using Aimless.^45^ The structure was determined by molecular replacement in Phaser^46^ using the structure of *Tt*AA10 (PDB ref 6RW7) as the search model. Following molecular replacement, a round of autobuilding using Buccaneer^47^ was performed. Subsequently, iterative rounds of manual model building, using COOT, and restrained refinement, using REFMAC, were performed.^48,49^ The copper ion and water molecules were added manually in COOT.^48^

### AlphaFold Prediction of CfAA10

The predicted structure of *Cf*AA10 was generated utilising the AlphaFold 3 Server.^50^ The full length amino acid sequence of *Cf*AA10 was input to be modelled with a copper ion resulting in the predicted model of *Cf*AA10.

### Electron Paramagnetic Resonance Spectroscopy

Continuous wave (cw) X-band EPR spectra were collected at 150 K for a frozen solution of the target protein in 20 mM sodium phosphate, 20 mM sodium acetate, 100 mM sodium sulfate buffer at pH 4.5–pH 8 for *Cf*AA10 and pH 5–pH 8 for *Cj*AA10BΔCBM_C‑Strep_. Data collection was performed using a Bruker micro EMX spectrometer using a frequency of ca. 9.30 GHz, with modulation amplitude of 4 G, modulation frequency of 100 kHz and a microwave power of 6.33 mW. The data was intensity-averaged over three scans. Simulations of the experimental data were performed using the Easyspin 5.2.28 open-source toolbox implemented by MATLAB R2020a software on a PC.^51^

### Electrochemistry Experimental Procedures

#### Electrochemical Set-Up

All electrochemical experiments were performed using a custom made gas-tight three--electrode cell using a pyrolytic graphite edge (PGE) working electrode fitted on an OrigaLys, OrigaTrod disk electrode rotator, a saturated calomel (SCE) reference electrode and a platinum wire counter electrode as shown in Figure 3A. Scanning electron microscopy (SEM) was performed to determine the roughness of the PGE working electrode. Figure 3B,C show the unit cell of the size of the unit cell of the *Cj*AA10BΔCBM crystal structure and how this compares to the surface of the electrode, in which 133 unit cells fit into the 1 μm scale bar.

**Figure 3:**
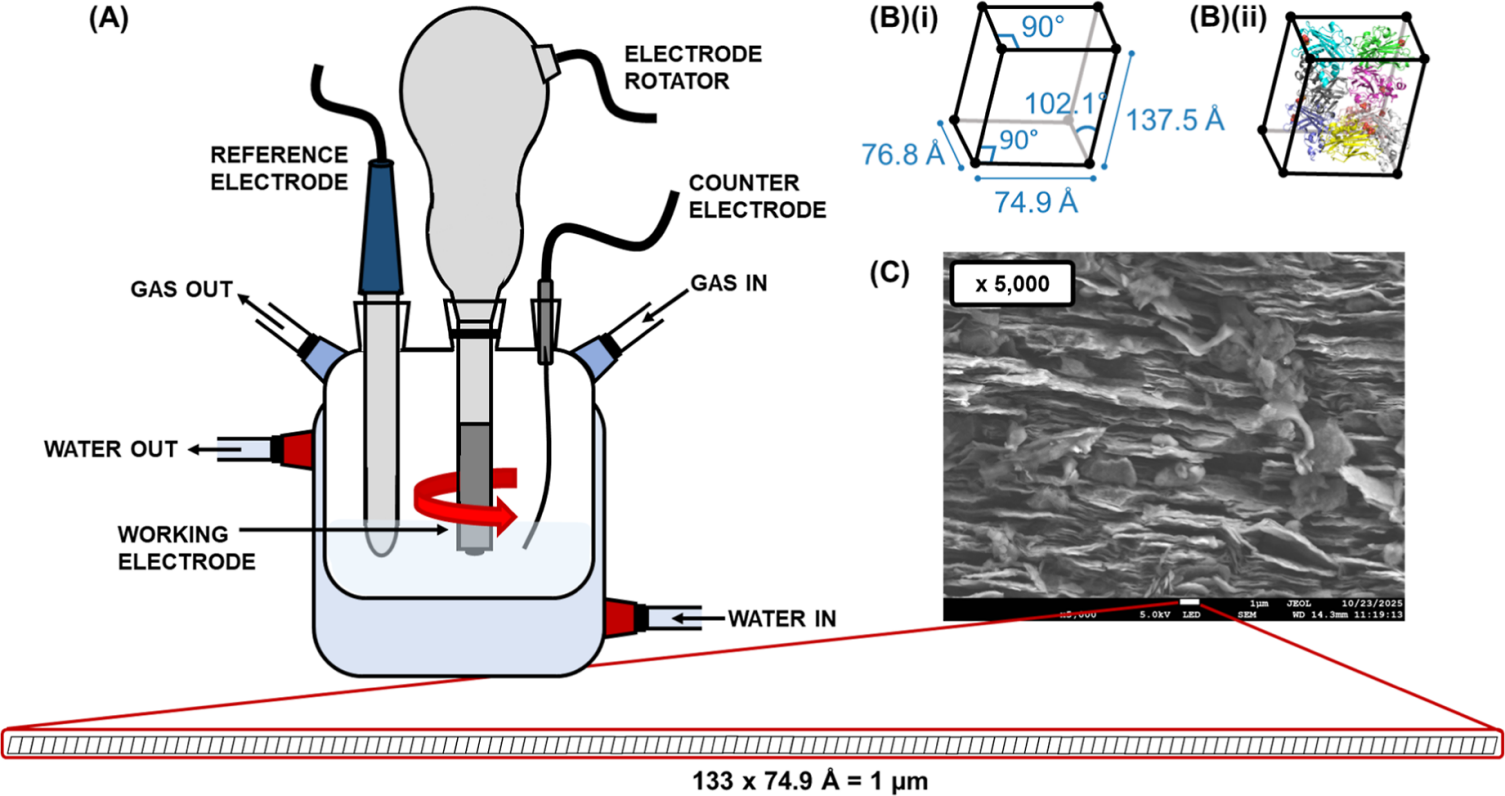
(A) Electrochemical cell set-up: three-electrode cell, with a pyrolytic graphite edge (PGE) working electrode at the end of an electrode rotator, saturated calomel reference electrode (SCE) and platinum wire counter electrode with a water jacket allowing for precise temperature control. (B)­(i) Unit cell taken from the crystal structure of *Cj*AA10BΔCBM and (B)­(ii) eight monomers of *Cj*AA10BΔCBM packed within the space group. (C) Scanning electron micrograph (5 kV accelerating voltage, 15 mm working distance, ×5000 magnification), showing that 133 unit cells fit into the space of the 1 μm scale bar.

All electrochemical experiments were conducted in buffer solution consisting of 20 mM sodium acetate, 20 mM sodium phosphate and 500 mM sodium sulfate. The pH of the electrochemical cell buffer solution was controlled by the addition of either sulfuric acid or sodium hydroxide and varies between experiments. The pH of each experiment is reported in figure captions. A water jacket surrounding the electrochemical cell allows for temperature control of the solution; unless stated otherwise experiments were carried out at 35 °C. All experiments were carried out in a MBRAUN glovebox under an inert nitrogen environment ([O_2_] < 50 ppm) with all buffer solutions being degassed under a flow of nitrogen gas before addition to the glovebox.

#### Electrode Polishing and Protein Film Formation

PGE electrode tips were produced in-house. Before they were used for PFV experiments they were first thoroughly polished with alumina slurry to ensure that the inherent capacitance of the electrode is low and to remove leftover impurities from the curing process. The PGE electrode was polished using 3 grades of alumina slurry (1 μm, 0.3 μm and 0.05 μm) in a figure of eight polishing motion in each direction before rinsing with Milli-Q water followed by sonication in acetonitrile for 5 min. This process takes place on the bench under ambient conditions. Polished electrodes were then ported into the glovebox - further polishing steps and protein film formation take place in the glovebox.

Before each experiment the PGE working electrode was polished using P1200 sandpaper. For protein-free experiments, the electrode was rinsed with Milli-Q water after polishing with sandpaper and was used as a “blank” control without further modification.

For LPMO experiments, the working electrode was also polished using P1200 sandpaper, protein samples were then drop-cast onto the freshly abraded surface of the working electrode and left to dry down to form a protein film (∼30 min). A protein film is typically formed of 10 μL of LPMO sample of 200–300 μM, however for less concentrated samples (<100 μM), a second 10 μL aliquot was drop-cast and allowed to dry down for a further 30 min to form a “double-film” of protein. Thus, modified, protein-coated electrodes were then transferred immediately to the electrochemical cell for testing.

### Electrochemical Methods

#### Direct Current Voltammetry

Direct current voltammetry (DCV) was carried out using an Ivium compactstat potentiostat with the corresponding software, Iviumsoft (version 4.1141). A direct current cyclic voltammetry experiment was performed to calibrate the saturated calomel reference electrode against the Standard Hydrogen Electrode (SHE) using a solution of 10 mM, pH 7.0 potassium ferricyanide in 200 mM sodium phosphate buffer, resulting in a value of *E*_ref_ = +243 mV vs SHE. Unless stated otherwise, experiments were carried out at 35 °C using a scan rate of 10 mV s^–1^ across a potential range of −160 to 640 mV vs SHE (−400 to 400 mV vs Ref).

#### Square Wave Voltammetry

Square wave voltammetry experiments were also carried out using an Ivium potentiostat and the Iviumsoft software. SWV experiments were recorded across a potential range from −570 to 540 mV vs SHE (−300 to 300 mV vs Ref.), using a 10 mV pulse amplitude, a 2 mV E_step_ and a 2 Hz frequency. As with DCV experiments SWV experiments were conducted at 35 °C with the pH of buffer solution reported in figure captions.

#### Chronoamperometry

Chronoamperometric experiments were performed using an Ivium potentiostat and the Iviumsoft software. All chronoamperometric experiments were recorded at a held potential of 75 mV vs SHE (−170 mV vs Ref.) with an interval time of 0.2 s at 35 °C with an electrode rotation rate of 2000 rpm. Direct current cyclic voltammograms were recorded directly before each chronoamperometry experiment.

### Electrochemical Assays

#### Hydrogen (H_2_O_2_) Peroxide Assay, Figure 7, Electrode Preparation & DCV

The ability of LPMOs to reduce H_2_O_2_ was investigated using a chronoamperometric assay in which the current response for an LPMO-functionalized electrode was recorded as H_2_O_2_ concentration was increased. Figure 7 depicts the results of one of these assays. A sample of LPMO was drop-cast onto the electrode surface and allowed to dry down to form a film. A DCV was carried out to determine the surface coverage of protein on the electrode via continuous cycling from −0.16 → 0.64 V vs SHE at 10 mV s^–1^ until a stable signal was achieved (approximately 3 to 9 cycles). The coverage is then calculated as described in Figure S8. The initial DCV is always carried out in pH 5.0 buffer solution to account for changes in the LPMO signal magnitude with pH; after collecting a DCV, the electrode can be rinsed, and the buffer solution can be exchanged for a solution of a different pH. All experiments are carried out at 35 °C, found to be the optimum temperature for LPMO electroactivity.

#### Hydrogen (H_2_O_2_) Peroxide Assay, Chronoamperometry

Chronoamperometry was performed at 75 mV vs SHE (−170 mV vs Ref.), a potential chosen to ensure that the enzyme was fully reduced, with the working electrode rotating at a rate of 2000 rpm. Upon application of the working potential, the system was left to equilibrate for ∼5 min or until the current remained constant. Once the current response had equalised, the first 100 μL aliquot of H_2_O_2_ solution was injected into the electrochemical cell using a pipette. The addition of H_2_O_2_ resulted in a spike of negative current corresponding to the electrocatalytic action of the LPMO reducing the H_2_O_2_. The current was left to equilibrate for 30 s before the injection of another 100 μL aliquot of H_2_O_2_ solution. This process was repeated until a total of 10 aliquots of H_2_O_2_ had been injected into the cell, resulting in a final concentration of ∼10 mM H_2_O_2_ in the bulk solution. The concentration of the aliquots of H_2_O_2_ solution depended on the volume of the electrochemical cell. Experiments were originally performed in an electrochemical cell requiring 50 mL of buffer solution, before a new cell was made in-house that required only 15 mL of buffer solution; this allowed the concentration of the aliquots of H_2_O_2_ solution to be decreased from 500 mM to 150 mM. The same experiment was repeated using a bare working electrode, allowing for the experiment to be corrected for the electrode’s ability to reduce H_2_O_2_ on its own. The extracted current from the control experiment was subtracted from that of the LPMO experiment and this data was analysed as described in the main text and Supporting Information. All assays were carried out under an atmosphere of N_2_ using a buffer composed of 20 mM sodium acetate, 20 mM sodium phosphate and 500 mM sodium sulfate.

#### Dioxygen (O_2_) Assay, Figure 8 , Electrode Preparation & DCV

A second chronoamperometric assay was developed to complement the H_2_O_2_ assay, allowing for the investigation of the ability of LPMOs to reduce O_2_. The results of one of these assays are shown in Figure 8. As with the H_2_O_2_ assay, the LPMO is adsorbed onto the electrode and a DCV is performed at pH 5.0 to determine the surface coverage of the LPMO on the electrode surface following the same protocol as described above.

#### Dioxygen (O_2_) Assay, Chronoamperometry

As above, chronoamperometry was performed at 75 mV vs SHE (−170 mV vs Ref.) with an electrode rotation rate of 2000 rpm at 35 °C. The concentration of O_2_ in the cell was controlled using Aalborg GFC17 mass flow controllers (MFCs), varying the relative rate of flow of N_2_ and compressed air with an overall flow rate of 100 mL min^–1^ maintained throughout the experiment with the system venting to atmospheric pressure. It was assumed that compressed air consisted of 20% O_2_ with the rest of the gas being made up of nitrogen and argon, both inert gases assumed to have no catalytic reactivity with LPMOs. The concentration of O_2_ in the cell was determined with relation to the partial pressure of O_2_ in the system using Henry’s Law. Assuming that when the gas flowing in the sealed electrochemical cell is 100% O_2_, the concentration of O_2_ in the buffer solution will be 1.3 mM. Upon application of the working potential, the current was allowed to stabilise under a flow of 100 mL min^–1^ N_2_ (100% N_2_). Once the current had levelled off, the flow of N_2‑_ into the system was reduced to 90 mL min^–1^ (90% N_2_) and a flow of compressed air was introduced at 10 mL min^–1^, thus increasing the concentration of O-_2_ in the cell to 2%. The current was allowed to stabilise before further increase in O_2_ concentration. The O_2_ concentration was increased in 2% increments by increasing the flow of compressed air until a final concentration of 10% O_2_ was achieved, equivalent to 130 μM O_2_. As the flow of compressed air was increased, the equivalent flow of N_2_ was decreased to ensure a constant overall flow rate of 100 mL min^–1^ into the cell. As above, the experiment was repeated using a bare electrode with no LPMO adsorbed to allow for correction for the electrode’s ability to reduce O_2_ on its own. The current from the control experiment was subtracted from that of the LPMO experiment and this data was analysed as described in the main text and Supporting Information. All assays were carried out using a buffer composed of 20 mM sodium acetate, 20 mM sodium phosphate and 500 mM sodium sulfate.

## Results and Discussion

### Protein Production and Structural Analysis

*Cellulomonas fimi* AA10 enzyme (*Cf*AA10) was produced complete with its native carbohydrate binding module (CBM), *Cellvibrio japonicus* AA10B was expressed with its naturally occurring C-terminal CBM and linkers removed (*Cj*AA10BΔCBM). In our previous work, the *Cf*AA10 and *Cj*AA10BΔCBM proteins were purified via C-terminal His tags.^12^ In order to confirm that these tags do not form secondary copper binding sites we generated new constructs in which the C-terminal His tag (*Cf*AA10_C‑His_ and *Cj*AA10BΔCBM_C‑His_) was replaced with a Strep-tag (*Cf*AA10_C‑Strep_ and *Cj*AA10BΔCBM_C‑Strep_). SDS-PAGE gels (Figure S3) and enzyme activity assays (Figures S4,S5 and Table 2) both showed that the change in purification tag did not negatively impact either the purity or activity of the final enzyme product and so the *Cf*AA10_C‑Strep_ enzyme was used for all subsequent work and will simply be referred to as “*Cf*AA10” for the remainder of this paper. Because we have crystal structure data for *Cj*AA10BΔCBM_C‑His_, we conducted our experiments on both the His- and Strep-tagged *Cj*AA10BΔCBM constructs, and saw no difference between them.

**Table 2:** Summary of Results From Both Electrochemical and Dye Assays^a^

		*Cj*AA10BΔCBM				*Cf*AA10			
		*i*_max_ (μA)	*K*_M_ (mM)	*k*_cat_ (s^–1^)	*k*_cat_/*K*_M_ (s^–1^ mM^–1^)	*i*_max_ (μA)	*K*_M_ (mM)	*k*_cat_ (s^–1^)	*k*_cat_/*K*_M_ (s^–1^ mM^–1^)
H_2_O_2_	electrochemistry*	1.6 ± 0.2	3.8 ± 1.1	0.87 ± 0.2	0.23 ± 0.08	4.7 ± 1.0	5.7 ± 1.4	1.4 ± 0.4	0.24 ± 0.09
	2,6-DMP assay^†^	-	0.028	0.015	0.54	-	0.070	0.11	1.57
O_2_	electrochemistry*	0.090 ± 0.01	0.14 ± 0.02	0.035 ± 0.004	0.24 ± 0.05	0.19 ± 0.05	0.070 ± 0.01	0.048 ± 0.01	0.69 ± 0.2
	amplex red^‡^	-	-	0.0012	-	-	-	0.0027	-

a See Figures 7 and 8 for electrochemical measurements. For dye assay data see Figures S4 and S5. Assay conditions are denoted as follows: *pH 5.0, 35C; ^†^pH 7.5, 30 °C; ^‡^pH 6.0, RT. For both *k*_cat_ and *K*_M_ values the quoted errors reflect the standard error calculated from experiments repeated in triplicate. These errors were propagated to generate those shown for the specificity constants as described in eq S4.

We determined the structure of *Cj*AA10BΔCBM_C‑His_ to 1.9 Å resolution by X-ray crystallography (Table S1 and Figure 4). Eight molecules were present in the asymmetric unit in our crystals. The complete polypeptide chain could be traced for all chains to the beginning of the C-terminal His tag. B-factor analysis suggested that Chain F represented the best ordered chain and so our structural analysis and description focuses on this chain only. The structure (Figure 4B) exhibits an immunoglobulin-like fold typical of AA10 LPMOs, with a flat binding face containing the histidine brace active site. Electron density attributed to the catalytic copper ion was observed, coordinated by the N-terminal and side chain amine groups of the histidine-1 (H1) residue, along with the side chain amine of a second histidine-109 (H109), as shown in Figure 4C. The coordination sphere around the copper is completed by a phosphate ion from the crystallization medium. A phenylalanine (F197) sits in an axial position relative to the copper, as has been observed in many members of AA10 LPMO family.^52–55^ In the opposite axial position, glycine is observed as opposed to the more common alanine that can be found in this position.^54^

**Figure 4:**
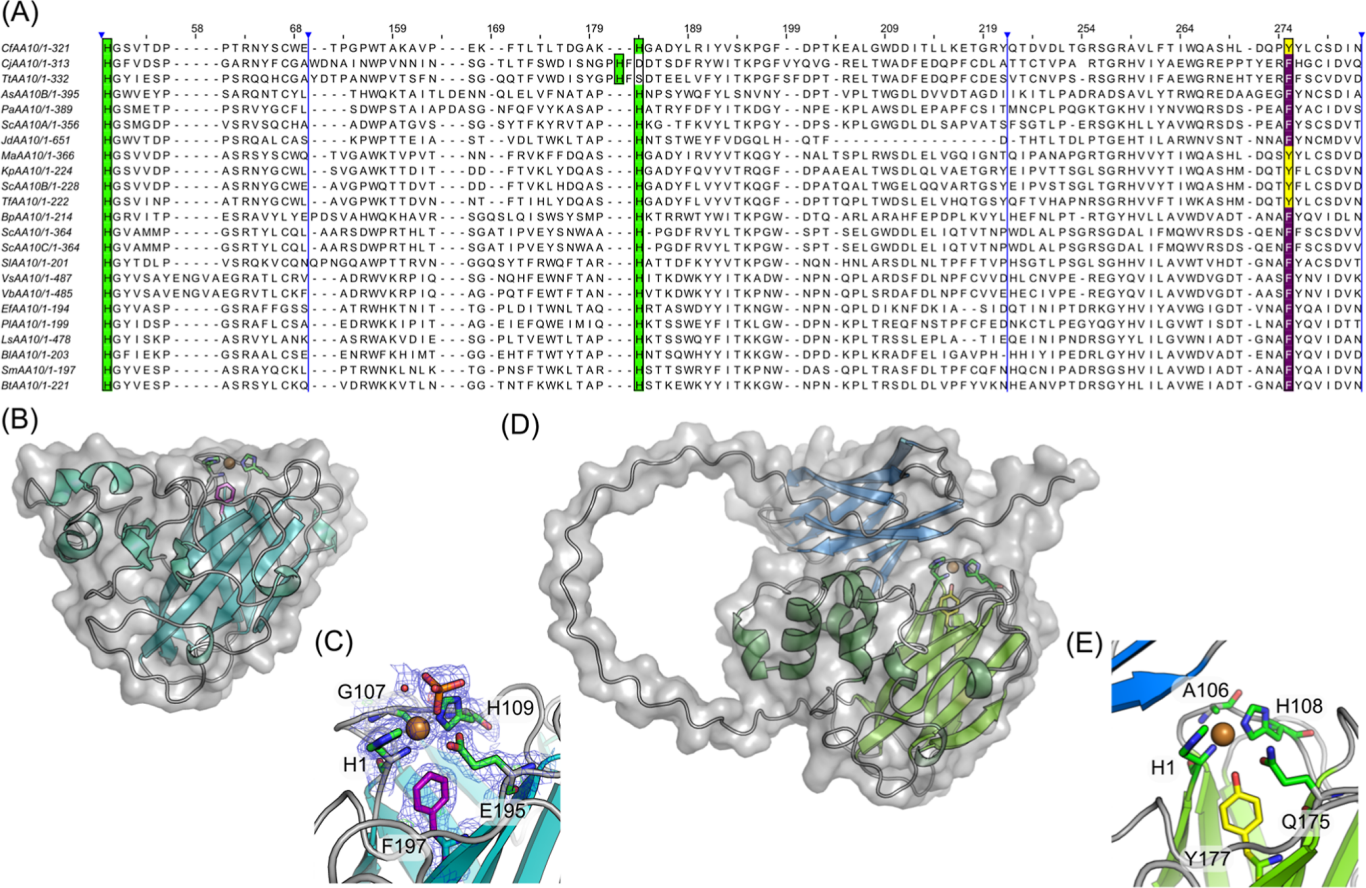
Sequence and structural analysis of AA10 LPMOs. (A) Multiple sequence alignment of *Cf*AA10 and *Cj*AA10BΔCBM with other structurally characterized AA10s listed in the CAZY database (http://www.cazy.org).^8^ The axial residues are highlighted in yellow or purple for tyrosine or phenylalanine, respectively. The active site histidine residues are shown in green. Numbering refers to the position within the multiple sequence alignment, with blue vertical lines indicating where sections of residues have been hidden. (B) Crystal structure of *Cj*AA10BΔCBM (PDB REF). The histidine brace is shown as sticks with carbon atoms coloured green, the active site copper is shown as an orange sphere and the phenylalanine located in the axial position is coloured purple. (C) Close up view of the active site of *Cj*AA10BΔCBM. The 2F_obs_-*F*_calc_ map is shown in blue contoured at 1σ and F_obs_-*F*_calc_ map shown at 3σ with positive density coloured green and negative density shown in red. All residues that have an atom within 4 Å of the active site copper are shown as sticks with the maps shown at a radius of 2 Å around these for clarity. Coordinating bonds to the active site copper in the equatorial positions are shown as dashed lines in black. (D) Predicted structure of *Cf*AA10 generated using the AlphaFold Server.^50^ The catalytic domain is shown with secondary structure elements coloured green and the CBM is shown with secondary structure elements coloured blue. The active site copper is shown as an orange sphere. Key active site residues are shown as sticks with the his brace shown with green carbon atoms and the tyrosine expected to be positioned axial to the active site copper shown with yellow carbon atoms. (E) Close up of the predicted active site for *Cf*AA10 showing the equivalent residues to those shown in panel C.

We were unable to determine experimentally a structure for *Cf*AA10, and so we predicted its structure using the AlphaFold Server instead.^50^ The resultant model, shown in Figure 4D,E, includes both the catalytic domain and its associated CBM. The predicted Local Distance Difference Test (pLDDT) values indicated high confidence in the structured regions of the LPMO domain and CBM (shown in blue, Figure S6A) and lower confidence in the likely disordered linker (shown in red, Figure S6A). Importantly, the global structure of the predicted model showed high similarity to the crystal structure for *Cj*AA10B, as demonstrated by the overlay in Figure S6C. Closer inspection of the predicted model confirms that tyrosine (Y177) is present at the axial position in this enzyme instead of the phenylalanine that was observed in our *Cj*AA10BΔCBM_C‑His_ structure. The presence of such a tyrosine is typical in the fungal AA9 LPMOs,^56–58^ but less common in AA10s, although not unprecedented in structurally categorised enzymes, as illustrated in the sequence alignment in Figure 4A.^52,59^

### pH 5 Electrochemistry

#### DCV Electrochemical Analysis of Cu^2+/1+^ Chemistry

Electrochemical experiments to investigate the copper redox chemistry of the two LPMOs were carried out using *Cj*AA10BΔCBM_C‑His_ and *Cf*AA10 adsorbed onto a pyrolytic graphite edge (PGE) working electrode. Figure 5 shows direct current voltammetry experiments, comparing enzyme-free “blank” electrodes to enzyme-functionalized electrodes. The blank experiments correspond to PGE electrode tips that have been polished with sandpaper and rinsed with MIlliQ water. After performing a DCV of the blank electrode (Figure 5 - grey lines), an LPMO sample is then drop-cast onto the electrode surface and allowed to dry down to a film before carrying out a second DCV (Figure 5 - purple/green lines). It is clear that peak-like features centred at approximately 0.3 V vs SHE can be attributed to the LPMO. The observed potential window is consistent with the range of LPMO Cu^2+/1+^ reduction potentials described in the literature,^36–38^ including the only other study of an AA10 LPMO by direct electrochemical investigation.^11^ Subsequent experiments provide more accurate midpoint potential quantification, see Table 1.

**Figure 5:**
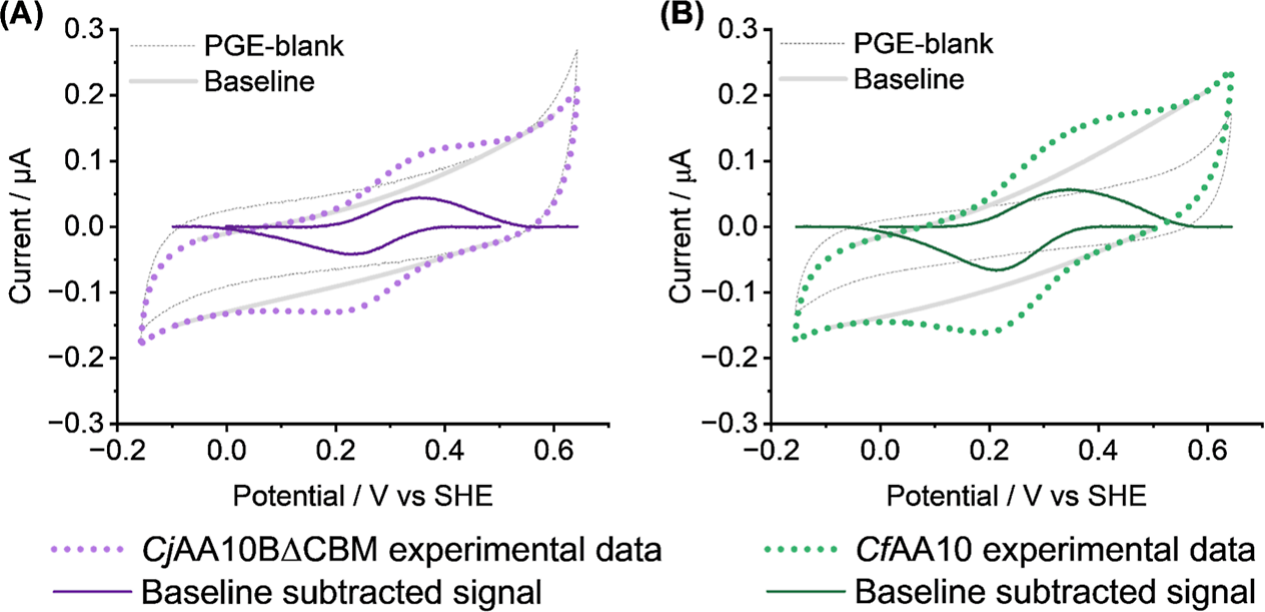
DCV experiments of (A) *Cj*AA10BΔCBM and (B) *Cf*AA10 adsorbed on the surface of a pyrolytic graphite edge (PGE) electrode. Scans were recorded across a potential range from −0.16 to 0.64 V vs SHE at 10 mV s^–1^ in a pH 5.0 buffer solution of 20 mM sodium acetate, 20 mM sodium phosphate, 500 mM sodium sulfate at 35 °C. In both cases, the sixth cycle is shown. “Blank”, protein-free experiments are shown with a grey dashed line and equivalent protein experiments are shown with a thick dotted purple (*Cj*AA10BΔCBM) or dotted green (*Cf*AA10) line. Computed non-Faradaic baseline projections are shown by a thick grey line, and the baseline subtracted enzyme signals are shown with a solid dark purple (*Cj*AA10BΔCBM) or dark green line (*Cf*AA10).

Figure 5 shows the sixth cyclic voltammogram measured on the protein films, and the nature of these signals is in line with that observed previously in protein voltammetry.^42^ Earlier scans of freshly adsorbed enzyme-films exhibit larger peaks, but continuous scanning, particularly while rotating the electrode, results in a rapid drop to a more stable peak current with greater equivalence between the oxidative and reductive peak heights (Figure S7). We cannot simply subtract the “blank” data from the enzyme experiments because the application of a protein film to an electrode changes the capacitance, so we therefore perform a manual baseline subtraction to remove a projected non-Faradaic current “background”, and isolate the Faradaic-only peak signals (Figure S8). The average area under the baseline subtracted peaks shown in Figure 5A equates to 8 × 10^–7^ C of charge, so assuming one electron transfer per LPMO, this is equivalent to 8 pmol of enzyme.

Taking the planar, geometric surface area of the electrode into account (circular diameter of 2 mm = area of 0.031 cm^2^), means that an unrealistic surface density of 0.26 nmol cm^–2^ LPMO coverage is calculated from the data in Figure 5. To further probe this, a control experiment in which flavin adenine dinucleotide (FAD), a redox active coenzyme, is adsorbed onto the surface of the working electrode, is shown in Figure S9. A value of 29 pmol was obtained for the number of moles of FAD adsorbed on the electrode surface; this value validates the assertion that 8 pmol of LPMO can form an adsorbed monolayer on our working electrodes and suggests that our electrodes have a very high roughness factor. Scanning electron microscopy (SEM) measurements (Figures 3 and S10) further explore this, our results correspond with work from Blanford and Armstrong in showing that the protein-accessible surface area of the edge plane of pyrolytic graphite can be orders of magnitude larger than the geometric surface area.^60^ This is illustrated in Figure 3 which compares one of our SEM images to the size of the unit cell of *Cj*AA10BΔCBM, validating that a far larger enzyme coverage is possible than may be expected based on the geometric surface area alone.

We are confident that the oxidative and reductive processes are attributable to active site copper because both structural and EPR studies (vide infra) confirm the presence of a single, standard histidine brace active site centre in each protein monomer. Additionally, as shown later in this paper, we see electrocatalytic enzyme activity which correlates with that seen in dye assays on LPMO in solution. Furthermore, control experiments conducted on copper-free “apo” protein do not show these signals, and the redox peaks from copper-loaded “holo” protein can also be reversibly recapitulated and silenced by stepwise in situ copper-loading and subsequent EDTA treatment of a film of apo-*Cj*AA10BΔCBM_C‑Strep_ (Figure S11A–C). When performing the same experiment with a blank electrode (Figure S11D) the results are not reproduced and this, along with the differing midpoint potentials for the two LPMOs, suggests that these signals must originate from enzyme-bound copper. Further control experiments are shown in Figures S12 and S13 in which the bare electrode is cycled in the presence of CuSO_4_, showing no LPMO-like signals forming when DCV experiments are carried out in either dilute or concentrated CuSO_4_ solution. Additionally, we also performed DCV measurements on our *Cj*AA10BΔCBM_C‑Strep_ protein to confirm that the identity of the purification tag had no effect on the copper signal (Figure S14) leading us to conclude that it is active site structural changes that are responsible for the differences between *Cf*AA10 and *Cj*AA10BΔCBM. It should be noted that the non-catalytic experiments were performed at 35 °C as this was discovered to be an optimal temperature for observing maximum signal intensity, as illustrated in Figure S15. We discuss the implications of signal-growth as a function of temperature in the next section.

#### SWV and DCV Quantification of Electron Transfer Rates of the Cu^2+/1+^ Transition

Square wave voltammetry (SWV) is an electrochemical technique that allows for enhancement of the current from electron transfer relative to the capacitive, charge-transfer background current that arises from double-layer rearrangements. Commonly, square wave analysis is conducted by first performing a background current subtraction and then analysing the residual current, assuming this arises purely from Faradaic electron-transfer processes.^61^ In order to extract kinetic information from this data, we fitted the total square wave current using a sum of a Butler–Volmer model of Faradaic current and a cubic polynomial to account for the non-Faradaic current contributions, described by eqs S1 and S2. The best-fit mathematical modelling of LPMO SWV measurements conducted at pH 5.0 (Figure 6) yielded similar electron transfer rate constants (*k*_et_) of approximately 0.5 ± 0.3 and 0.4 ± 0.2 s^–1^ for the Cu^2+/1+^ transition for *Cf*AA10 and *Cj*AA10BΔCBM_C‑His_ respectively. However, we acknowledge that non-idealities in the DCV data (reductive peak position may be insensitive to scan rate) may mean that a more complex electron transfer model might be justified. We return to this concept in later experiments which demonstrate the important impact that pH has on LPMO non-catalytic signals.

**Figure 6:**
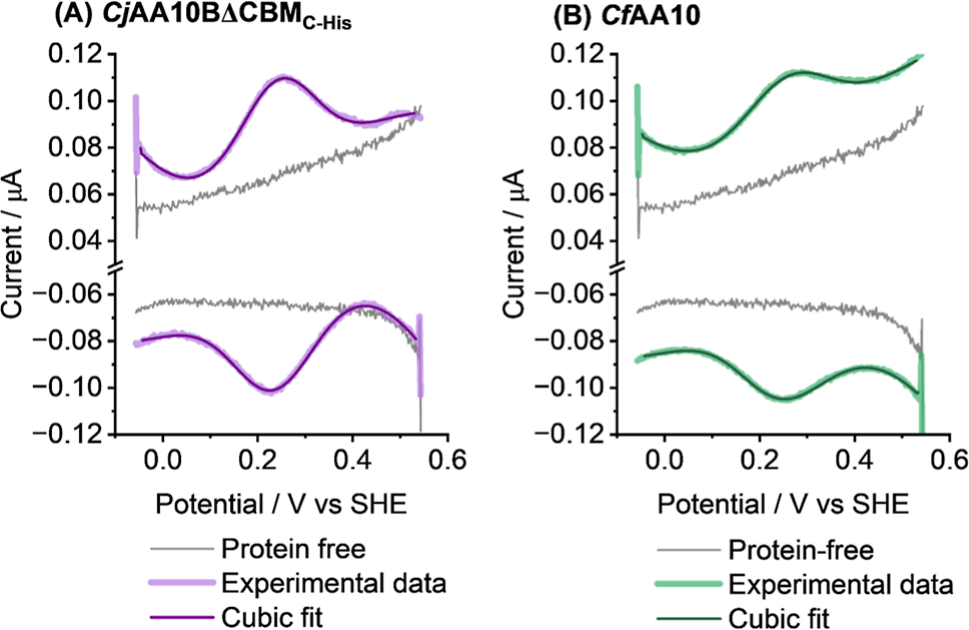
Square wave voltammetry plots of (A) *Cj*AA10BΔCBM_C‑Strep_, pH 5.0 (thick, light purple line); (B) *Cf*AA10, pH 5.0 (thick, light green line). Data from enzyme-free control experiments is shown by grey lines, and lines of best fit are shown using thin, dark purple or dark green lines. To generate the best-fit, a simulation is used in which the Butler–Volmer equation is used to account for Faradaic (electron-transfer) contributions to the current, and a cubic polynomial equation is used to account for capacitive background current contributions. All experiments were carried out in a pH 5.0 buffer solution of 20 mM sodium acetate, 20 mM sodium phosphate, 500 mM sodium sulfate at 35 °C with a stationary electrode under an atmosphere of N_2_ and measured across a potential range from −0.06 to 0.54 V vs SHE using a 10 mV pulse amplitude, a 2 mV E_step_ and a 2 Hz frequency.

As shown in Table 1, the rates of LPMO electron transfer obtained for *Cf*AA10 and *Cj*AA10BΔCBM_C‑His_ from SWV are consistent with values for other LPMOs acquired via electrochemical methods by Cordas et al.^11^ and Zouraris et al.^37^ Whilst copper proteins such as azurin are often associated with much faster electron transfer rates,^62–65^ the LPMO rates are not anomalous relative to other type-II copper proteins. For example, an electrochemical study of galactose oxidase (a type-II copper enzyme which catalyses the oxidation of d-galactose within fungi) reported that the Cu^2+/1+^ signal seen in DCV experiments quickly diminishes as the scan rate increases, consistent with slow *k*_et_ and similar to what we observe with the AA10 LPMOs (see Figure S16).^66^ A slow rate of conversion between the different copper oxidation states suggests a high reorganisation energy associated with the electron transfer process.^14^ This correlates with the increase in DCV and SWV Cu^2+/1+^ peak size as a function of temperature (Figure S15).^67–69^ The growth in signal intensity as the temperature is increased from 5 °C to 20 °C, and then further raised to 35 °C at pH 5.0, is consistent with the electron transfer rate increasing.

### pH 5 Electrocatalysis

We have developed electrochemical assays to investigate the ability of *Cf*AA10 and *Cj*AA10B to reduce H_2_O_2_ and O_2_, as detailed in Figure 2. The electrode potential is held at a constant value of 75 mV vs SHE (∼200 mV more negative than the *E*(Cu^2+/1+^) values determined above) as the substrate concentration is increased. To measure the ability of the enzyme to reduce H_2_O_2_, stepwise addition of aliquots of H_2_O_2_ solution were injected into the cell. For the equivalent O_2_ experiment, the concentration of substrate was increased by using mass flow controllers (MFCs) to vary the relative flow of N_2_ to air through the sealed electrochemical cell. The working electrode upon which the LPMO is adsorbed was rotated at a rate of 2000 rpm to ensure rapid hydrodynamic flux through the experiment, and we therefore assume that the rate of mass transport of the substrate to the LPMO was non-limiting. We also note that by rotating the electrode we will have removed any weakly bound enzyme from the surface, and assume minimal contributions from solution enzyme electrochemistry due to the low maximum bulk solution concentration of enzyme (if all enzyme applied to the electrode dissolved into the bulk 15 mL electrochemical cell volume, the final concentration would be approx. 150 nM).

Figure 7 shows the data extracted from the chronoamperometric H_2_O_2_ assay. Figure 7A shows the current response seen for *Cj*AA10BΔCBM_C‑His_ over a concentration range from 0 mM to ∼10 mM H_2_O_2_. The same information is displayed for *Cf*AA10 in Figure 7B. Enzyme free data is depicted by the grey lines. For comparison, Figure S17 shows LSV experiments which also compare enzyme activity to current from EDTA-treated, Cu-free, redox inactive, so-called “apo-LPMO” in the presence (5 mM and 10 mM) and absence of H_2_O_2_. Importantly, these experiments show that although some H_2_O_2_ reduction is catalysed when the electrode is coated in apo-LPMO, the amount of electrocatalysis is substantially decreased relative to H_2_O_2_ reduction by both *Cj*AA10BΔCBM_C‑Strep_ and *Cf*AA10. The onset potential overlaps with the voltage window at which Cu^2+^ to Cu^1+^ reduction is observed in LPMO experiments in the absence of substrate. These data show that we can utilise electrochemistry as a direct measure of the electrocatalytic reduction of H_2_O_2_ by an LPMO.

**Figure 7:**
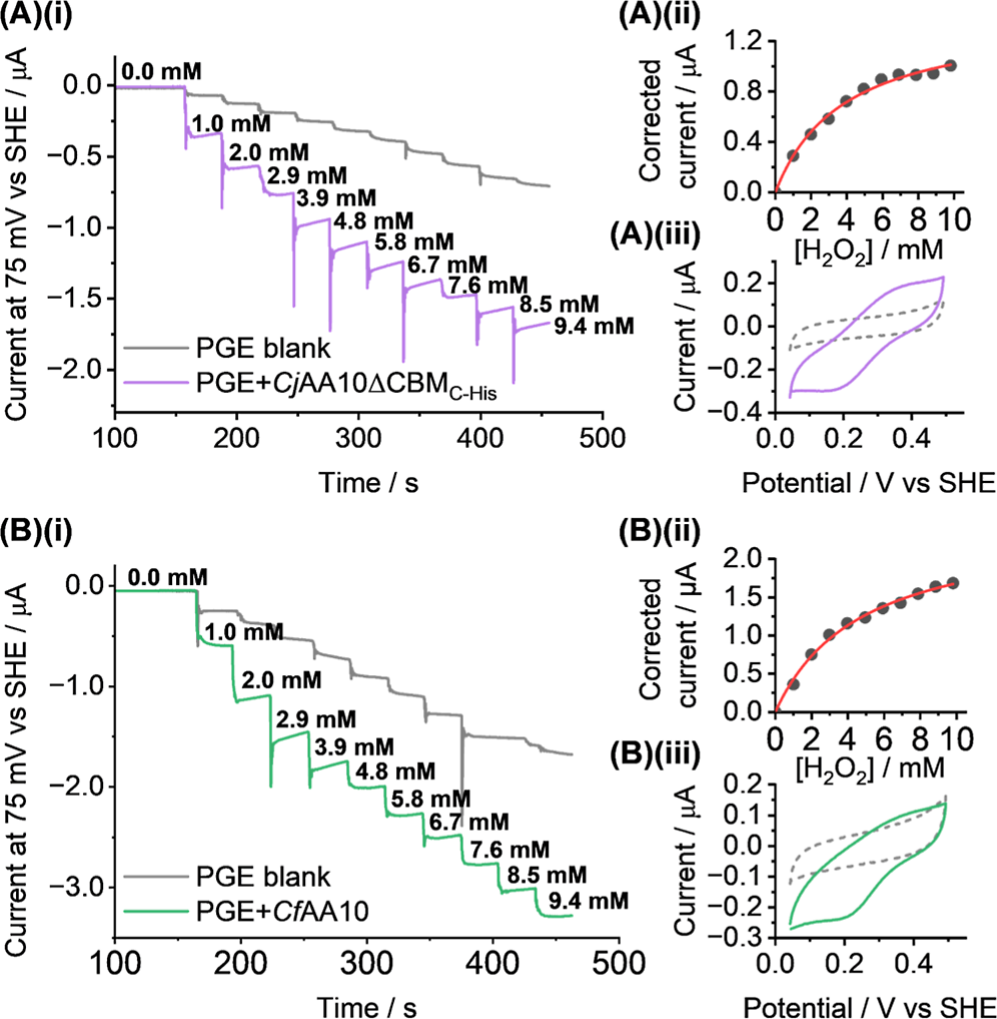
Hydrogen peroxide activity assay. (A)­(i) Chronoamperometric current response of *Cj*AA10BΔCBM_C‑His_ (purple line) across a range of 0 mM to ∼9.4 mM H_2_O_2_ at a constant potential of 75 mV vs SHE and the equivalent data for an LPMO-free control experiment (grey line). (A)­(ii) Michaelis–Menten plot of the enzyme (blank electrode corrected) current response vs concentration of H_2_O_2_, black points are experimental data and the red line is a best-fit. (A)­(iii) DCV of *Cj*AA10BΔCBM_C‑His_ (solid line) before the addition of the first aliquot of H_2_O_2_ across a range of −0.16 to 0.64 V vs SHE at 10 mV s^–1^ and an equivalent enzyme-free measurement (dotted line). (B)­(i-iii) equivalent experimental data for the CfAA10 enzyme (green lines). All experiments were carried out in a pH 5.0 buffer solution of 20 mM sodium acetate, 20 mM sodium phosphate, 500 mM sodium sulfate at 35 °C with a rotating electrode (2000 rpm) under an atmosphere of N_2_.

Figure 8 shows the complementary O_2_ assay, measured over a concentration range from 0 μM to ∼130 μM O_2_, with Figure 8A showing the current response for *Cj*AA10BΔCBM_C‑His_ and Figure 8B showing equivalent data for *Cf*AA10. In both cases, the LPMO measurements are compared to enzyme-free “blank” control data. For comparison, LSV experiments were also conducted in the presence and absence of O_2_ (Figure S18). These confirm that 75 mV vs SHE is a sensible voltage at which to conduct electrocatalytic chronoamperometry since inactive “apo” control measurements show less reductive current than those conducted on active-site containing “holo” enzymes at this potential.

**Figure 8:**
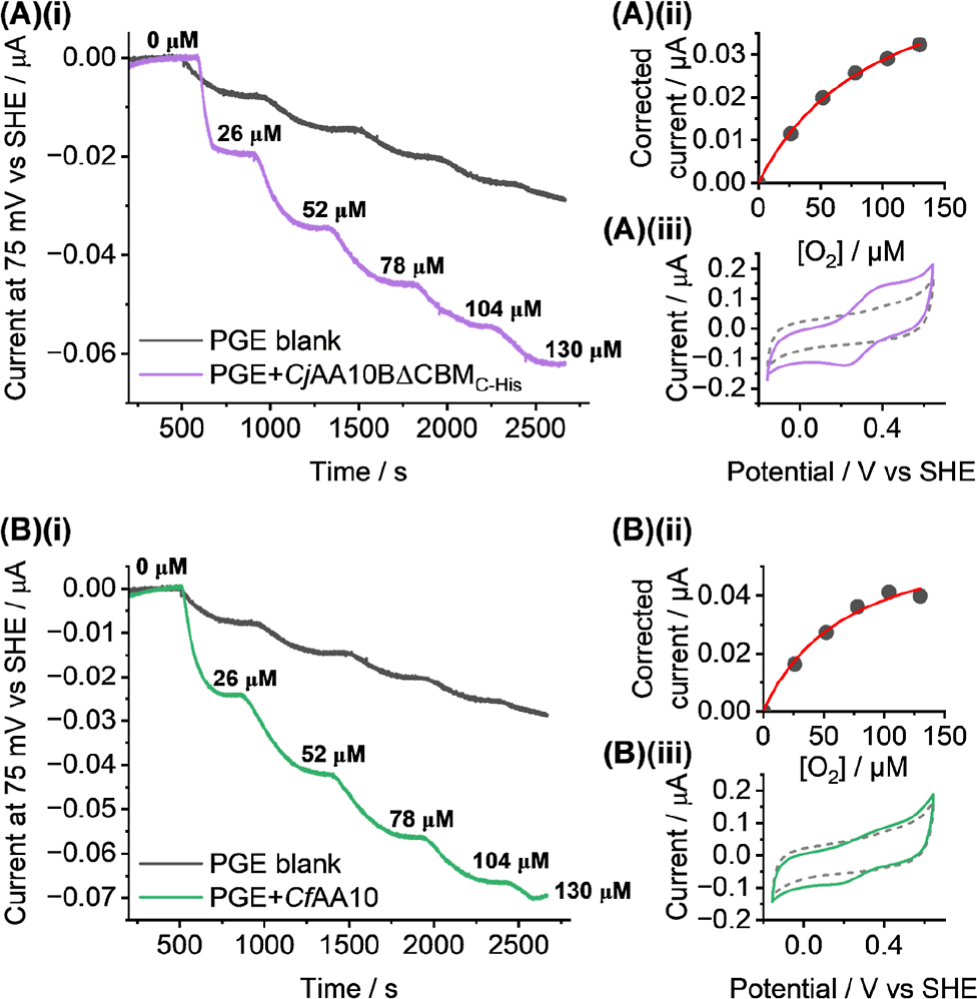
Dioxygen activity assay. (A)­(i) Chronoamperometric current response of *Cj*AA10BΔCBM_C‑His_ (purple line) across a range of 0 μM to 130 μM O_2_ at a constant potential of 75 mV vs SHE and the equivalent data for an LPMO-free control experiment (grey line). (A)­(ii) Michaelis–Menten plot of the enzyme (blank electrode corrected) current response vs concentration of O_2_, black points are experimental data and red line is best-fit. (A)­(iii) DCV of *Cj*AA10BΔCBM_C‑His_ (solid line) before the increase of O_2_ concentration across a range of −160 to 640 mV vs SHE at 10 mV s^–1^. Experiments were carried out at pH 5.02 in 25 mL of buffer solution at 35 °C. (B)­(i–iii) Equivalent experimental data for the *Cf*AA10 enzyme (green lines). All experiments were carried out in 20 mM sodium acetate, 20 mM sodium phosphate, 500 mM sodium sulfate buffer solution at 35 °C with a rotating electrode (2000 rpm) under a controlled atmosphere of different air and N_2_ mixtures of total gas flow rate 100 mL min^–1^.

To ensure that the observed current response correlates to oxygen reduction and not to the enzyme reducing H_2_O_2_ produced by the bare electrode two different analyses were conducted, see Figure S19. Firstly, a series of calculations was carried out to estimate the concentration of H_2_O_2_ that would theoretically be produced by a bare electrode exhibiting the current response shown in Figures 8A­(i)/B­(i) and (S19A). Secondly, the rotation rate of an LPMO-modified electrode was pulsed between 0 and 2000 rpm to confirm that under stationary conditions the current dropped in a manner attributable to substrate depletion, i.e. when O_2_ flux drops the reductive current is attenuated despite such conditions facilitating an increase in the local concentration of H_2_O_2_ at the electrode surface, see Figure S19B.

In order to measure the constant-potential data displayed in Figures 7 and 8, the non-enzymatic reduction current is subtracted from the LPMO current at each substrate concentration, as shown for H_2_O_2_ in Figure S20. Since this enzyme-only/“corrected” electrical current provides a direct measure of the rate of electron transfer catalysed by the LPMO, it is directly proportional to the enzymatic reaction velocity, *v*, defined in the Michaelis–Menten equation,^70^ accordingly, we re-state the equation as eq 1. The red lines in Figure 7 and Figure 8 show the best-fit between eq 1 and the corrected, enzyme-only data, permitting extraction of the Michaelis constant, *K*_M_, for both enzymes. We also demonstrate the suitability of this form of analysis by displaying the Lineweaver–Burk plots, shown in Figures S21 and S22. The straight-line nature of these plots and extrapolation of similar *K*_M_ and *i*_max_ values (Tables S2 and S3) further confirms the suitability of the Michaelis–Menten model for describing the LPMO electrocatalysis of H_2_O_2_/O_2_ reduction.

1 $$i=\frac{i_{\max}\left[S\right]}{K_{M}+\left[S\right]}$$

The maximum catalytic current, *i*_max_, is equated to the enzymatic rate of catalysis, *k*_cat_, using eq 2: in which the number of electrons, *n*, is 2; *F* is Faraday’s constant; Γ is the coverage of enzyme molecules per unit area; and *A* is the area of the electrode. Integrating the Cu^2+/1+^ Faradaic current peaks from the substrate-free DCV experiments measured prior to the chronoamperometry (peak integration shown in Figure S8) provide a measure of the total amount of LPMO attached to the electrode (i.e. gives a value for Γ × *A*). Carrying out this analysis enables calculation of the *k*_cat_ values reported in Table 2.

2 $$k_{cat}=\frac{i_{\max}}{nF\Gamma A}$$

The values reported below fall within the ranges expected from the literature, with Bissaro et al. reporting values between 0.017 and 0.17 s^–1^, and Hangasky et al. reporting a *k*_cat_ value of 0.28 s^–1^ for the reactivity of an AA9 with O_2_.^18,71^ Similarly, values for H_2_O_2_-reduction can be found in the range of 0.0020 s^–1^ to 2.95 s^–1^,^26,72–77^ indicating that results that we observe are within an expected range.

For comparison to values of *k*_cat_ and *K*_M_ extracted electrochemically, we also performed a series of dye assays in order to extract the same kinetic constants using established methods. The 2,6-dimethoxyphenol (2,6-DMP) assay was used to monitor the H_2_O_2_-reduction activity at pH 7.5 and 30 °C yielding *K*_M_ and *k*_cat_ values shown in Table 2, see Figure S4.^26^ There are no directly comparable literature values for the AA10 enzymes studied here, and reported values for the 2,6-DMP assay of different LPMOs, shown in Table S4, vary quite substantially. The most comparable data for *Ba*LPMO10A exhibits a *k*_cat_ value of 0.042 s^–1^.^72^ The higher *k*_cat_ and *K*_M_ values for the electrocatalytic H_2_O_2_ assay in comparison to the 2,6-DMP assay are notable. Whereas the enzyme is directly “wired” to the source of electrons (the electrode) in our electrochemical assay, the 2,6-DMP must diffuse to the enzyme in the dye assay. A higher *k*_cat_ value is therefore expected, and the corresponding increase in K_M_ means that the specificity constant of the two assay types are consistent for *Cj*AA10BΔCBM.

It is also notable that the electrochemistry and dye assay measurement of H_2_O_2_ reduction are carried out at different pH and temperature. Indeed, a substantive limitation of the 2,6-DMP assay is that the dye molecule redox potential varies with pH, as shown in Figure S23.^26^ When compared to the midpoint potential measured for the LPMOs studied here, it is suggestive that at pH < 6.0 there will no longer be a negative Gibbs Free Energy for electron transfer from the dye molecule to the LPMO. Corroborating this, Breslmayr et al.^26^ report enhanced assay sensitivity at pH 7.5 compared to pH 6.0. When we attempted to carry out the 2,6-DMP H_2_O_2_-reduction assay at pH 5.0 we were unable to detect any difference between *Cf*AA10 experiments and an enzyme-free control (Figure S4). This highlights the strength of utilising an electrochemical assay; because the potential of the electrode can be freely adjusted, there is the ability to explore the relationship between pH and enzymatic activity, as described later.

For comparison with the O_2_ reduction electrocatalysis, the widely utilised horseradish peroxidase (HRP) Amplex Red assay was also used to monitor the production of H_2_O_2_ from O_2_ by the AA10 LPMOs.^34,78,79^ Using ascorbate as a reductant, *Cj*AA10BΔCBM_C‑His_ and *Cf*AA10 were assayed at pH 6.0, resulting in *k*_cat_ values of 0.0012 ± 0.000025 s^–1^ and 0.0027 ± 0.00014 s^–1^, respectively (Table 2). Previous studies on the enzymes report *i*_cat_ values for *Cj*AA10BΔCBM_C‑His_ and *Cf*AA10_C‑His_ of 0.0004 s^–1^ and 0.0009 s^–1^ respectively^12^ and we attribute our higher values to variability in the oxygen sensitive reductants and reporters utilised within the assay. The use of an electrode in our chronoamperometric assay removes this concern. Attempts to carry out the Amplex Red assay at pH 5 were unsuccessful (Figure S5), likely because resorufin exhibits poor fluorescence below its p*K*_a_ of ∼6.^80,81^

As mentioned above, the electrochemically extracted *k*_cat_ and *K*_M_ values are higher when compared to those obtained using dye assays; this has been observed in previous studies comparing catalytic rates extracted from PFV experiments to those extracted from solution-based assays.^11,19,82,83^ Figure 2 illustrates the critical differences between the methods of assaying substrate reduction; highlighting that the electrochemistry directly reports on the electron transfer without intermediaries, the rotation of the electrode actively drives diffusion of substrate to the enzyme, and the film configuration of the enzyme immobilised onto the electrode provides instantaneous access to an electron supply. This is an important advantage of the electrochemical methods, in comparison both dye assays require passive diffusion of substrate and reducing agents to the LPMO, and, in the case of the Amplex Red assay, product H_2_O_2_ and the reporter molecule (Amplex Red) are also required to diffuse to the horseradish peroxidase. A further advantage of our electrochemical toolkit is the ability to measure *K*_M_ for O_2_-reduction, a value that we are unable to extract from the Amplex Red assay.

As seen in Table 2, The substrate has a major effect on the rate of catalysis for both enzymes. The *k*_cat_ values measured via electrochemistry for both AA10s are at least an order of magnitude higher for H_2_O_2_ reduction compared to O_2_ reduction. This corresponds with a substantive number of studies.^11,19,82,83^ In experiments which are comparable to the carbohydrate-free conditions of our electrochemistry, Bissaro et al. report single reoxidation measurements of AA10 LPMO Cu^I^ being converted to Cu^II^, with a 2000-fold second order rate increase with H_2_O_2_ over O_2_.^84^ In carbohydrate containing measurements, Jones et al.^25^ used stopped-flow and rapid freeze-quench EPR spectroscopy to probe an AA9 LPMO from *Hypocrea jecorina*, showing that the turnover rates increased by over 2 orders of magnitude when using H_2_O_2_ as the catalytic co-substrate instead of O_2_.

As shown in Figure 4, *Cj*AA10BΔCBM_C‑His_ and *Cf*AA10 differ in the secondary coordination sphere of their active sites. Our experiments consistently show that the Tyr containing *Cf*AA10 has a higher *k*_cat_ than the Phe containing *Cj*AA10BΔCBM_C‑His_. This aligns with what is observed by Cordas et al.,^11^ when the natural phenylalanine of an AA10 (*Sc*LPMO10C) was substituted for a tyrosine residue they also observed an increase in activity. We also note a consistent, small difference in the *E*(Cu^2+/1+^) midpoint potential of *Cf*AA10 vs *Cj*AA10BΔCBM_C‑His_ which may also indicate subtle active site tuning by changes in the axial positions around the active site copper ion.

### pH Dependence of Cu^2+/1+^ Signal

#### Notable Growth of Cu^2+/1+^ Signal upon Decreasing pH from 6 to 4

Figure 9 shows a series of DCV experiments carried out at different pH values to determine how proton concentration tunes the non-catalytic copper redox chemistry of the LPMO enzymes. Instead of the simple, Nernstian −59 mV pH^–1^ horizontal peak-position shift that would indicate one proton per electron proton-coupled electron-transfer,^85^ Figure 9 shows that for films of either *Cf*AA10 or *Cj*AA10BΔCBM_C‑His_, steadily decreasing the pH of the experiment (exchanging the buffer solution in the electrochemical cell while retaining the same protein coated working electrode) causes a growth in the peak current.

**Figure 9:**
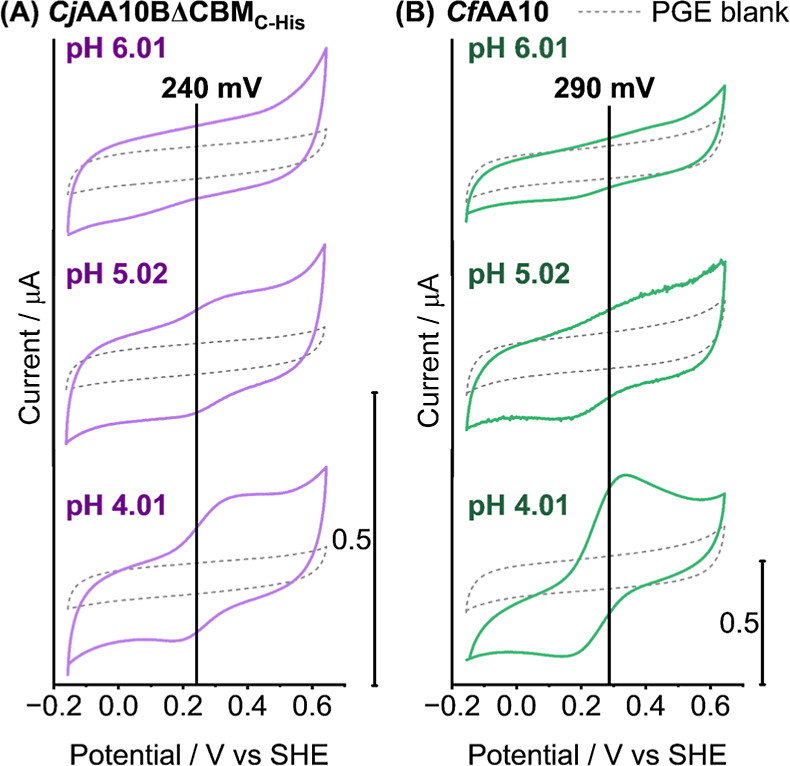
Direct current voltammetry (DCV) analysis of the Cu^2+/1+^ redox signal of both (A) *Cj*AA10BΔCBM_C‑His_ (solid purple lines) and (B) *Cf*AA10 (solid green lines). LPMO samples were drop-cast onto the surface of a freshly abraded PGE working electrode before being added to the electrochemical cell containing pH 6.0 buffer solution. DCV experiments were then measured from −0.16 to 0.64 V vs SHE at 10 mV s^–1^. The LPMO-coated electrode was then removed from the electrochemical cell and the buffer was replaced with pH 5.0 solution, the DCV experiment was then repeated on the same protein film before a final buffer exchange to pH 4.0 and a further DCV experiment. Enzyme-free experimental data is included for comparison (dotted grey lines). All experiments were carried out in 20 mM sodium acetate, 20 mM sodium phosphate, 500 mM sodium sulfate buffer solution at 35 °C with a stationary electrode under an atmosphere of N_2_. Vertical black lines plot the midpoint potential determined from the SWV shown in Figure 6.

Further experiments were carried out to confirm that the effect of pH shown in Figure 9 is not a result of gradual enzyme activation as a function of time. The same trend of larger signals under more acidic conditions is still observed when experiments are commenced at pH 4.0 before buffer exchange to raise the pH. The reversibility of this process is also proven via cycling the pH up and down and showing the same reversible increase in signal with decreasing pH, shown with both DCV and SWV in Figures S24 and S25.

To quantify the change in peak size as a function of pH the SWV measurements shown in Figure S26A,B were performed. Starting at pH 4.0, an anodic sweep was recorded, before the buffer solution was exchanged to pH 4.5 and another anodic sweep was performed. This buffer exchange followed by SWV measurement was repeated in increasing increments of 0.5 pH units up to pH 6.0. In order to account for gradual “film-loss” (a steady drop in signal as a function of time, relating to enzyme desorption or deactivation over time–these processes cannot be distinguished from one another experimentally and are therefore referred to under the catchall term of “film-loss”),^86–88^ a pH 4.0 control experiment was conducted, with SWV scans taken at corresponding timepoints to the pH-change experiment. As shown in Figure S26, The decrease in signal intensity as a function of increasing pH is more extensive than the rate of signal loss as a function of time. The film-loss corrected change in peak area with pH is shown in Figure S26C. The SWV measurements taken at pH 4.0, pH 4.5 and pH 5.0 were computationally analysed as described previously. The best fit values for the Cu^2+/1+^ electron transfer rates show no significant change with pH, instead it is the coverage of electroactive species on the electrode (quantified as Γ) which increases with decreasing pH (Table 3). This suggests that the redox-peak size decreases with increasing pH because a deprotonated species is formed which is not capable of rapid electron transfer to/from the electrode.

**Table 3:** Electron Transfer Rate and Electroactive Coverage Quantities Extracted from Fitting *Cf*AA10 Non-catalytic Square Wave Voltammetry Data Measured at Different pH (Figure S27)

	*k*_*ET*_^*RED*^ / *s* ^–1^	Γ/mol cm^–2^
pH 4.0	0.1(±0.001)	3.63 × 10^–9^ (±4 × 10^–11^)^a^
pH 4.5	0.1(± 0.001)	1.80 × 10^–9^ (±2 × 10^–11^)^a^
pH 5.0	0.1(± 0.001)	6.23 × 10^–10^ (±6 × 10^–12^)^a^

a Reported errors refer to the uncertainty in the simulation fit - MCMC analysis shown in Figure S27.

One possible explanation for the pH-induced change in the electrochemistry is that the solution pH may influence the surface electrostatic charge of the enzymes in a manner that results in reorganization/reorientation on the electrode surface. If the orientation at lower pH had a shorter electrode-to-copper distance, this could result in the larger overall Faradaic current response observed at decreasing pHs. First, protparam (https://web.expasy.org/protparam)^89^ was utilised to estimate the pI of *Cf*AA10 and *Cj*AA10BΔCBM_C‑Strep_ as 5.81 and 5.75, respectively.^89^ These values are higher than the pH range over which the significant changes in electrochemical peak size are observed, and therefore a change in the net charge of the protein does not explain the electrochemistry. We therefore also looked at the surface charge maps for the protein structures. This indicated minimal charge on the active site-containing face of *Cf*AA10 (Figure S28), and a negatively charged region on the opposite face. Conversely, *Cj*AA10BΔCBM_C‑Strep_ exhibits a negatively charged patch next to the active site (Figure S29). As both LPMOs exhibit opposing regions of charge, it seems unlikely that shifts in orientation due to pH-induced surface charge change are responsible for the very similar pH-dependent change in current response seen in both enzymes. An alternative explanation for this change with pH is the possibility of structural changes within or surrounding the active site that result in a new configuration that is more conducive to electrochemical characterisation.

#### EPR to Probe Structural Active Site Changes as a Function of pH

To investigate if changes in electrochemical signals with pH arise from structural changes in the active site, we employed EPR spectroscopy. We hypothesize that a protonated form of the active site facilitates more rapid electron transfer to/from the electrode in the absence of H_2_O_2_/O_2_. To probe the pH-structure relationship, we conducted a CW-EPR X-band spectra pH-titration of both *Cj*AA10ΔCBM_C‑Strep_ and *Cf*AA10, as shown in Figure 10. Both enzymes were studied up to pH 8.0 as this was the buffer pH at which the crystal structure of *Cj*AA10BΔCBM_C‑Strep_ was obtained. Unfortunately, at pH < 5.0, *Cj*AA10BΔCBM_C‑Strep_ precipitated out of solution. However, we were able to prepare a pH 4.5 EPR sample of *Cf*AA10. Carrying out pH cycling with *Cf*AA10, starting at pH 6.0, followed by buffer exchange to pH 5.0 and then finally to pH 7.0, also allowed us to ensure that any changes we observed were reversible and not related to denaturation of the enzyme.

**Figure 10:**
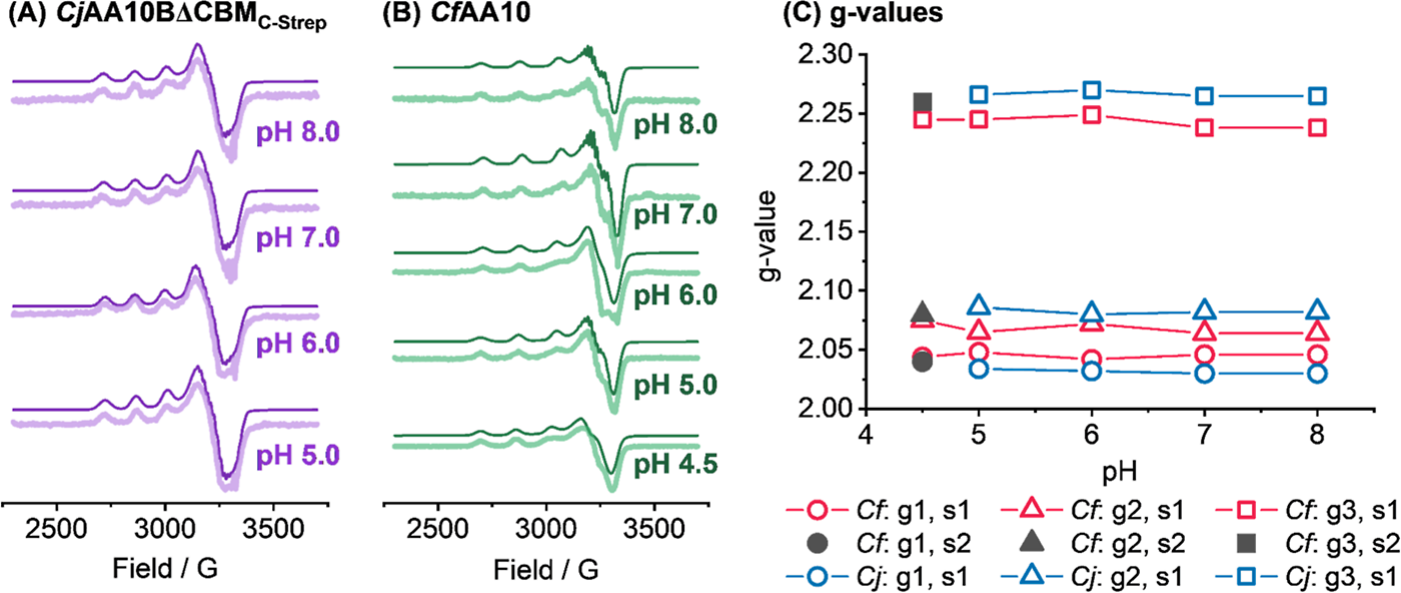
(A) X-band EPR spectra of *Cj*AA10ΔCBM_C‑Strep_ from pH 5.0 to pH 8.0 showing both experimental (light purple line) and simulated (dark purple line) data. (B) Equivalent CW EPR spectra for *Cf*AA10 from pH 4.5 to pH 8.0; in this case experimental data is shown in light green with the simulated data overlaid in dark green. All simulated data is vertically offset from the experimental data-set. (C) *g*-values for the simulated spectra; a single species (s1) is modelled in the pH 5.0–pH 8.0 range but a second species (s2) is included in the simulation of the pH 4.5 *Cf*AA10 data.

The EPR spectra and their simulations are shown in Figures 10 and S30. For both *Cj*AA10BΔCBM_C‑Strep_ and *Cf*AA10, only one species (one set of three *g*-values) was required to account for the data at pH 5.0, 6.0, 7.0, and 8.0. The simulations included hyperfine splitting values for three nitrogen ligands, two sp^2^ N-histidine ligands and one sp^3^ N-terminus, in accord with the crystallography data (Table S5 and Figure S31). The consistency in all the simulation parameters across this pH range show that these samples all arise from the same active site structure which we refer to as “Species 1”. Conversely, for the pH 4.5 *Cf*AA10 data, when a scaled simulation of species 1 was subtracted from the experimental trace, the substantial residual, see Figure S30, indicated the presence of a second species. A best fit to the experiment was obtained when the total EPR simulation comprised a mixture of 44% of Species 1 and 56% of a new species we designate “Species 2”. As shown in Figure 10, the biggest difference between the *g*-values of *Cf*AA10 Species 1 and Species 2 is the g3 value. Structural data in the literature has indicated that under low pH conditions histidine-protonation may cause a loss of metal–nitrogen binding.^90^

If we assume that Species 2 contains a more protonated form of the active site than the state which dominates in higher pH experiments, then according to the Henderson–Hasselbalch equation the 44% to 56% ratio of protonated-to-deprotonated species at pH 4.5 equates to a p*K*_a_ value of 4.6. Although EPR data support a p*K*_a_ around 4.6, quantifying this from electrochemistry alone is difficult, since the proportion of deprotonated species cannot be directly measured. If we assume a p*K*_a_ of 4.6 and that only the protonated active site state gives rise to SWV peaks, then we can find the maximum SWV peak areas at low pH via fitting to the Henderson–Hasselbalch equation, and thus generate the plot shown in Figure 11. These data confirm that the p*K*_a_ extracted from the EPR experiments can be used to generate a good fit to the electrochemical data. This analysis makes it unlikely that protonation of the non-terminal copper-binding histidine residue of the active site histidine-brace is responsible for the pH-induced changes in the non-catalytic electrochemistry and EPR. In the low pH structural study of *Ls*AA9,^90^ a much lower p*K*_a_ value of 3.5 was estimated for the formation of a flipped-histidine active site conformation. A wider structural analysis of the protonation state of active site histidines in a broader range of AA9 enzymes further confirms that p*K*_a_ < 3.7 is expected for the imidazole ring nitrogen of the non-terminal histidine.^91^

**Figure 11:**
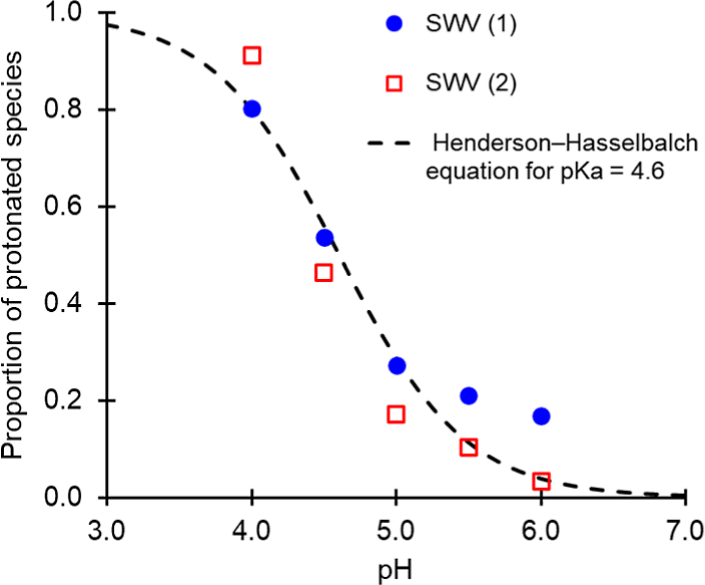
Overlay of proportion of protonated species versus pH from Henderson–Hasselbalch equation with p*K*_a_ = 4.6 (black dashed line), and separately analysed SWV (1) and SWV (2) datasets (blue solid circles and red open squares, respectively) which have been normalised using maximum peak area values calculated from fitting to the Henderson–Hasselbalch equation assuming both p*K*_a_ = 4.6, and only the protonated form of the active site is visible in non-catalytic SWV experiments.

As shown in Figure 12, comparison of the AlphaFold predicted active site structure of *Cf*AA10 to literature data for *Nc*LPMO9C suggests that both a *Cf*AA10 secondary coordination sphere glutamic acid residue, Glu54, and a copper adjacent histidine residue, His172, are potential candidates for residues that could change protonation state over the correct pH range to match our estimated p*K*_a_ of 4.6.^91–93^ However, *Cj*AA10 does not contain a third His close to the active site, leading us to speculate that protonation of a glutamate is the most likely candidate for the change in non-catalytic electrochemical response at low pH. However, further experiments, beyond the scope of this paper, are required to test this hypothesis.

**Figure 12:**
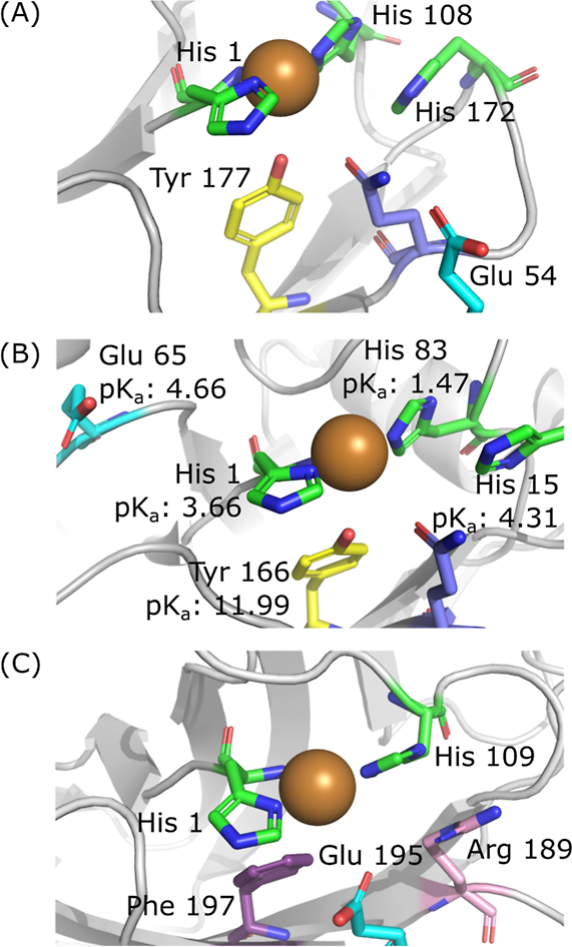
(A) Predicted active site structure of *Cf*AA10 with colour code: histidine = green; tyrosine = yellow; glutamic acid = light blue; glutamine = lilac. (B) Active site from crystal structure of *Nc*AA9C (PDB: 4D7U)^93^ with residues colour coded as in (A) and p*K*_a_ value labels from calculations by Zhou et al.^92^ (C) Active site from the crystal structure of *Cj*AA10B shown in Figure 4. Residues are coloured as in (A) with arginine = pink.

### pH Dependence of Electrocatalysis

Given the relationship established between pH and the Cu^2+/1+^ chemistry of the LPMOs, we wanted to see if pH would also tune the LPMO electron-transfer chemistry in the presence of substrate. Both H_2_O_2_ and O_2_ reduction were investigated for *Cj*AA10ΔCBM_C‑Strep_ and *Cf*AA10 at pH 6.0 and pH 7.0 using the chronoamperometric methods described above. Figures S32–S34 and Tables S6–S8 show that there are no consistent trends in the *i*_max_ or K_M_ values as a function of pH across the range pH 5.0 to pH 7.0. We also calculate *k*_cat_ values by accounting for non-catalytic peak signals measured at pH 5.0 prior to the chronoamperometry (Tables S6 and S7). We note that this introduces some unquantified error as although we wait for the protein film to stabilise before proceeding with the chronoamperometry measurements, we are not able to correct for the unknown film loss which will occur through the duration of the chronoamperometry measurements. However, when a Student *t*-test was performed to compare the variation in *k*_cat_ and *K*_M_ between pH 5.0 and pH 6.0, and pH 5.0 and pH 7.0, both *Cj*AA10BΔCBM and *Cf*AA10 show no statistical significance (*p* > 0.05) except in the difference between the *K*_M_ values for *Cf*AA10 between pH 5.0 and pH 6.0 (Table S8 and Figure S34). Given the lack of any significant relationship between catalytic current response and pH, we therefore assume that substrate-binding negates the effect of protonation on the electroactivity of the Cu^2+/1+^ oxidation state cycling, suggesting that the pH dependence of the electroactive surface coverage is not present in the catalytic regime. To account for variation in coverage between experiments and allow for conversion of *i*_max_ to *k*_cat_, we precede each chronoamperometric experiment with a substrate-free DCV that measures the protein coverage at pH 5.0. This allows for consistent normalisation of the catalytic current to enzyme coverage in a manner that is not affected by the fact that less of the enzyme performs non-catalytic electron transfer at higher pH.

The ability of LPMOs to perform O_2_- and H_2_O_2_-reductive activation chemistry of the copper active site over a range of pH is not unexpected. Previous studies have shown that LPMOs exhibit structural stability and catalytic activity over a wide range pH range,^94^ from as low as pH 3 to as high as pH 10,^95^ with different LPMOs exhibiting different pH optima;^73,96^ indeed, Li et al.^97^ report catalytic activity of *Cf*AA10 between pH 4.5 and 10.5. Notably, other enzymes expressed by *C. fimi* associated with cellulose degradation exhibit a range of pH optima, with examples at pH 5,^98^ pH 7 ^99^ and pH 8.5.^100^ As *Cf*AA10 is the only known LPMO from *C. fimi*, the flexibility regarding pH exhibited by *Cf*AA10 may allow for effective utilisation of cellulose in a wide range of environments.

Table 4 compares the average *k*_cat_ and *K*_M_ values obtained from repeat experiments, see Tables S6–S8 for individual experimental data. The non-catalytic Cu^2+/1+^ electron transfer rates (Table 1) are consistently lower than the *k*_cat_ for H_2_O_2_, indicating that the non-catalytic redox process is not the rate-determining step in catalysis. We interpret this as indicative of a change in the geometry of the active site upon H_2_O_2_-binding that decreases the activation energy required for oxidation state cycling of the copper centre, i.e. H_2_O_2_-binding increases the rate of redox cycling in the LPMO active site. Given that the substantial relationship between electron transfer rate and pH is also absent from the catalytic dataset we further speculate that H_2_O_2_ binding also disrupts the protonation process which dominates the non-catalytic data (putatively assigned to a glutamate residue in our above analysis).

**Table 4:** Summary of the Average Values for the Catalytic Rate Constant (*k*_cat_) and the Michaelis–Menten Constant (*K*_M_) Extracted for Both *Cj*AA10ΔCBM and *Cf*AA10 Across a Range of pHs, From pH 5.0 to pH 7.0^a^

H_2_O_2_					
		*Cj*AA10BΔCBM		*Cf*AA10	
*k*_cat_/s^–1^	pH 5.0	0.87 ± 0.2	(*n*_exp_ = 3)	1.4 ± 0.4	(*n*_exp_ = 3)
	pH 6.0	0.58 ± 0.04	(*n*_exp_ = 3)	2.4 ± 0.5	(*n*_exp_ = 2)
	pH 7.0	0.81 ± 0.09	(*n*_exp_ = 3)	0.61 ± 0.004	(*n*_exp_ = 2)
*K*_M_/mM	pH 5.0	3.80 ± 1.1	(*n*_exp_ = 3)	5.7 ± 1.4	(*n*_exp_ = 3)
	pH 6.0	4.8 ± 0.4	(*n*_exp_ = 3)	11 ± 0.5	(*n*_exp_ = 2)
	pH 7.0	5.7 ± 0.6	(*n*_exp_ = 3)	6.0 ± 0.5	(*n*_exp_ = 2)
$\frac{k_{cat}}{K_{M}}$ /s^–1^mM^–1^	pH 5.0	0.23 ± 0.08	(*n*_exp_ = 3)	0.24 ± 0.09	(*n*_exp_ = 3)
	pH 6.0	0.12 ± 0.01	(*n*_exp_ = 3)	0.22 ± 0.05	(*n*_exp_ = 2)
	pH 7.0	0.14 ± 0.02	(*n*_exp_ = 3)	0.10 ± 0.009	(*n*_exp_ = 2)
O_2_					
*k*_cat_/s^–1^	pH 5.0	0.035 ± 0.004	(*n*_exp_ = 3)	0.048 ± 0.01	(*n*_exp_ = 3)
	pH 6.0	0.063 ± 0.01	(*n*_exp_ = 2)	0.15 ± 0.06	(*n*_exp_ = 2)
	pH 7.0	0.031 ± 0.004	(*n*_exp_ = 2)	0.14 ± 0.03	(*n*_exp_ = 2)
*K*_M_/mM	pH 5.0	0.14 ± 0.02	(*n*_exp_ = 3)	0.070 ± 0.01	(*n*_exp_ = 3)
	pH 6.0	0.10 ± 0.0003	(*n*_exp_ = 2)	0.31 ± 0.003	(*n*_exp_ = 2)
	pH 7.0	0.07 ± 0.007	(*n*_exp_ = 2)	0.90 ± 0.2	(*n*_exp_ = 2)
$\frac{k_{cat}}{K_{M}}$ /s^–1^mM^–1^	pH 5.0	0.24 ± 0.05	(*n*_exp_ = 3)	0.69 ± 0.2	(*n*_exp_ = 3)
	pH 6.0	0.62 ± 0.1	(*n*_exp_ = 2)	0.47 ± 0.2	(*n*_exp_ = 2)
	pH 7.0	0.42 ± 0.07	(*n*_exp_ = 2)	0.15 ± 0.04	(*n*_exp_ = 2)

a The *n*_exp_ value reports the number of experimental repeats. The specificity constant, *k*_cat_/*K*_M_, is calculated by dividing the average *k*_cat_ value by the average *K*_M_ value at each pH. The individual experimental data from which averages and errors have been calculated is displayed in Tables S6–S8. For both *k*_cat_ and *K*_M_, the quoted errors reflect the standard error calculated from the repeat experiments (*n*_exp_ refers to the number of experimental repeats). These errors were propagated (as described in Supporting Information) to generate those shown for the specificity constants.

Both AA10 enzymes continue to display much higher *k*_cat_ values for H_2_O_2_ reduction compared to O_2_ reduction across the wider pH range, as summarised in Table 4. However, because the *K*_M_ values for O_2_ are lower this results in specificity constants that are comparable between H_2_O_2_ and O_2_, see Figure 13. Therefore, our data point towards LPMO enzymes being capable of effectively utilising both H_2_O_2_ and O_2_ as a co-substrate during in vivo carbohydrate degradation.

**Figure 13:**
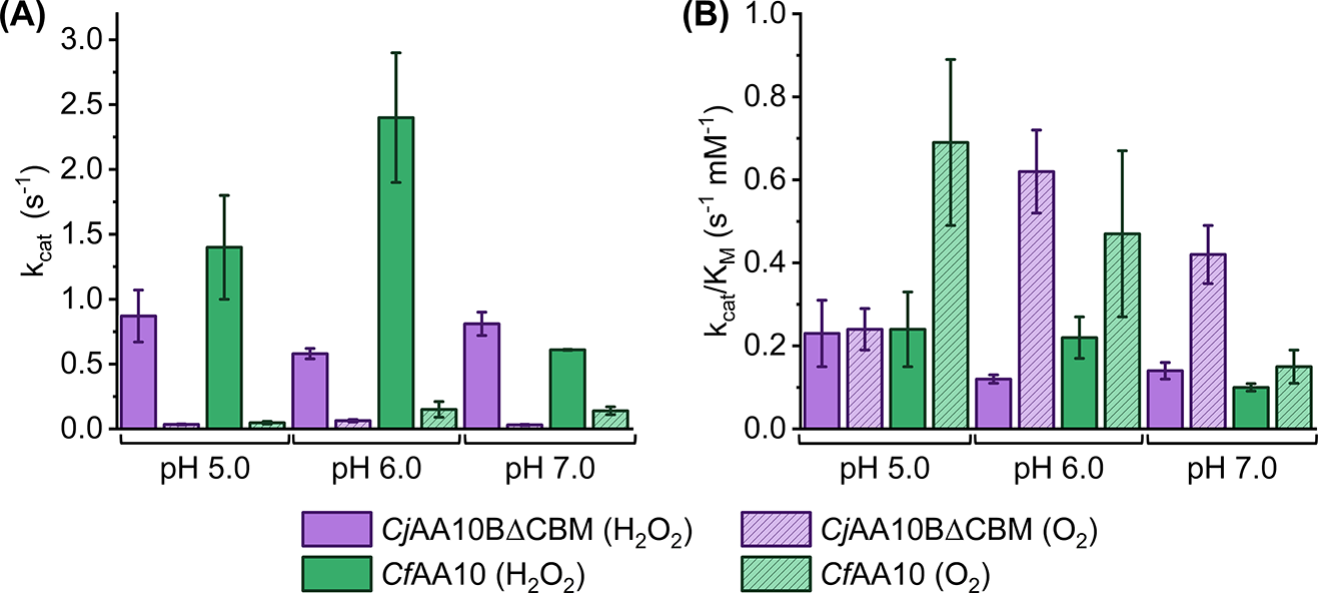
Bar charts showing a comparison of (A) *k*_cat_ vs (B) specificity constant over a pH range of pH 5.0 to pH 7.0 for *Cj*AA10BΔBCM (purple) and *Cf*AA10 (green).

## Conclusions

We have described a new electrochemical approach to probing the H_2_O_2_- and O_2_- reducing activity of LPMOs (summarised in Figure 2). Our method allows us to directly “wire” the enzyme to the surface of the electrode which removes the need for intermediaries and reporter molecules, resulting in a more direct insight into LPMO activity. The rotation of the electrode also removes limitations surrounding the diffusion of the substrate to the LPMO by ensuring that the solution is well mixed at all times. This method therefore provides a means of studying LPMO electrocatalytic activity over a broad range of conditions.

Chronoamperometric assays have allowed for the extraction of values for *k*_cat_ and *K*_M_ for the catalytic reduction of *Cf*AA10 and *Cj*AA10BΔBCM with H_2_O_2_ and O_2_ at pH 5.0, 6.0, and 7.0. Comparison of these kinetic constants shows that H_2_O_2_ appears to be the favorable substrate for both AA10s, an observation that is consistent with what has previously been reported in the literature. However, it should be noted that when considering the affinity-coupled specificity constant, there is less of a difference between LPMO activity with H_2_O_2_ vs O_2‑_, suggesting that whilst H_2_O_2_ is kinetically favorable, the enzymes are capable of turning over both substrates in the absence of carbohydrates.

Beyond substrate specificity, we also report a strong correlation between pH and the non-catalytic redox chemistry of both LPMOs. In the absence of H_2_O_2_ or O_2_ a protonation event with a p*K*_a_ of ∼4.6 results in the conversion of the enzyme into a state that can carry out facile electron transfer to and from the electrode. As pH increases, the electrochemical signal decreases, indicating that the deprotonated form of the enzyme is unable to perform Cu^2+/1+^ oxidation state cycling on a time scale that can be detected by electrochemistry. We assign the protonation state change to an active site process based on complementary EPR studies. Notably, when H_2_O_2_ or O_2‑_ are present, the catalytic activity does not substantially change across the pH range studied. This suggests that the binding of the substrate perturbs the active site in a manner that facilitates electron transfer to the copper. This flexibility is likely beneficial as *C. fimi* only expresses one known LPMO, and therefore a sustained performance across a variety of pH values may allow for growth in a wider range of environmental conditions. It should also be noted that *Cf*AA10 consistently outperforms *Cj*AA10BΔBCM over the range of conditions studied, indicating that the residue in the axial position to the copper is influencing the activity of the enzyme. These observations suggest that the secondary coordination sphere of the LPMO plays a key role in tuning the electroactivity of LPMOs; future experiments, beyond the scope of this work, should provide insight into the influence of different residues on substrate activity.

We hope that this newly described method of assaying the reductive ability of LPMOs provides a starting point to inform future experiments and broadens the number of enzyme classes to which electrochemical studies can be usefully applied. In particular, the fact that current directly represents catalytic velocity makes enzyme electrochemistry a very powerful enzyme assay tool. We aim to tune LPMO active site residues to further investigate the structure–function relationship of these enzymes, in hopes that further understanding the role of the active site architecture in tuning the chemistry will allow us to enhance their activity with a potentially major impact on the way we handle biomass. We also hope to unpick the intriguing relationship between pH and non-catalytic electron transfer, utilising a combination of mutagenesis and electrochemical assays to probe the mechanism of this phenomenon. Future work will also focus on further developing these assays through the introduction of carbohydrates to facilitate the investigation of LPMOs in an environment more closely resembling how they would be found in vivo.

## Supplementary Material



## Notes

Potential LPMO mechanisms; full sequence alignment data; biochemical characterization data; additional electrochemical data; EPR simulation details (PDF)
