# Supplementary material for: Electrochemical Insight into the Copper Redox Chemistry and H2O2 and O2 Reducing Capability of Two AA10 Lytic Polysaccharide Monooxygenases
Source: ACS Electrochem. 2026 Jan 15;2(2):239–57. doi: 10.1021/acselectrochem.5c00266 (PMC12884475; doi:10.1021/acselectrochem.5c00266)
Supplement: Supplementary file 1 [file ec5c00266_si_001.pdf]

Supporting Information for: Electrochemical Insight into the Copper Redox Chemistry and H_2O_2 and O_2 Reducing Capability of Two AA10 Lytic Polysaccharide Monooxygenases

Ella K. Reid,[$a] Connor G. Miles,[$a] Henry O. Lloyd-Laney,[b] Alison K. Nairn,[a] Jessie Branch,[c] Nicholas Garland,[a] Nicholas D. J. Yates,[a] Alex Ascham,[a] Paul H. Walton,[a] Glyn Hemsworth,[*c] and Alison Parkin[*a]

[a]Department of Chemistry, University of York, Heslington, York, YO10 5DD, United Kingdom

[b]Department of Computer Science, University of Oxford, OX1 3QG, United Kingdom

[c]Astbury Centre for Structural Molecular Biology and School of Molecular and Cellular Biology, Faculty of Biological Sciences, University of Leeds, Leeds, LS2 9JT, United Kingdom

[$]These two authors contributed equally

*Corresponding authors' email addresses: alison.parkin@york.ac.uk, g.r.hemsworth@leeds.ac.uk

Contents of SI

Contents of SI 1
Potential LPMO mechanisms - H2O2 vs O2 3
Further Experimental Methods for Protein Production and Biochemical Assays 4
Plasmid Generation 4
Plasmid Amplification 4
His-tagged AA10 Protein Purification 5
Strep®II-tagged AA10 Protein Expression 5
Protein Concentration Assays 6
Biochemical Assays 6
2,6-Dimethoxyphenol Dye Assay 6
Amplex RedTM Assay 7
Full Sequence Alignment Data 8
Non-Electrochemical Characterization Data 9
SDS-PAGE 9
Solution Assay Data 10
2,6-Dimethoxyphenol Assay Data 10
Amplex RedTM Assay Data 11
Alphafold 3 Structural Prediction of CfAA10 13
Supplementary Electrochemical Data Analysing Reversible Copper Redox Chemistry at pH 5.0 14
Control Experiments Exploring the Stability of LPMO Films 14
Control Experiments on Free Cu and Apo-LPMO 15
Control Experiments Showing that the Substrate-free Voltammetric response of CjAA10BΔCBM is Unaffected by the Identity of the Purification Tag 16
Analysis of Square Wave Voltammetry Data 17

Supplementary Electrocatalysis Data **21**
Hydrogen Peroxide DCV at pH 5.0 21
Dioxygen DCV at pH 5.0 22
Accounting for H2O2 production at the bare electrode 23
Electrocatalytic Assay Analysis 23
Current extraction 23
Michaelis Menten and Lineweaver-Burk Analysis - H2O2 24
Michaelis Menten and Lineweaver-Burk Analysis - O2 26
Peak Integration of Cyclic Voltammograms 26
Literature Values for the 2,6-Dimethoxyphenol Assay 28
Solution Voltammetry of 2,6-dimethoxyphenol at Different pH 29
Impact of pH on Non-Catalytic Copper Redox Activity **30**
LPMO Surface Charge 33
EPR Simulation Details **35**
Electrocatalytic Data at Different pHs **39**
References **43**

Potential LPMO mechanisms - H_2O_2 vs O_2

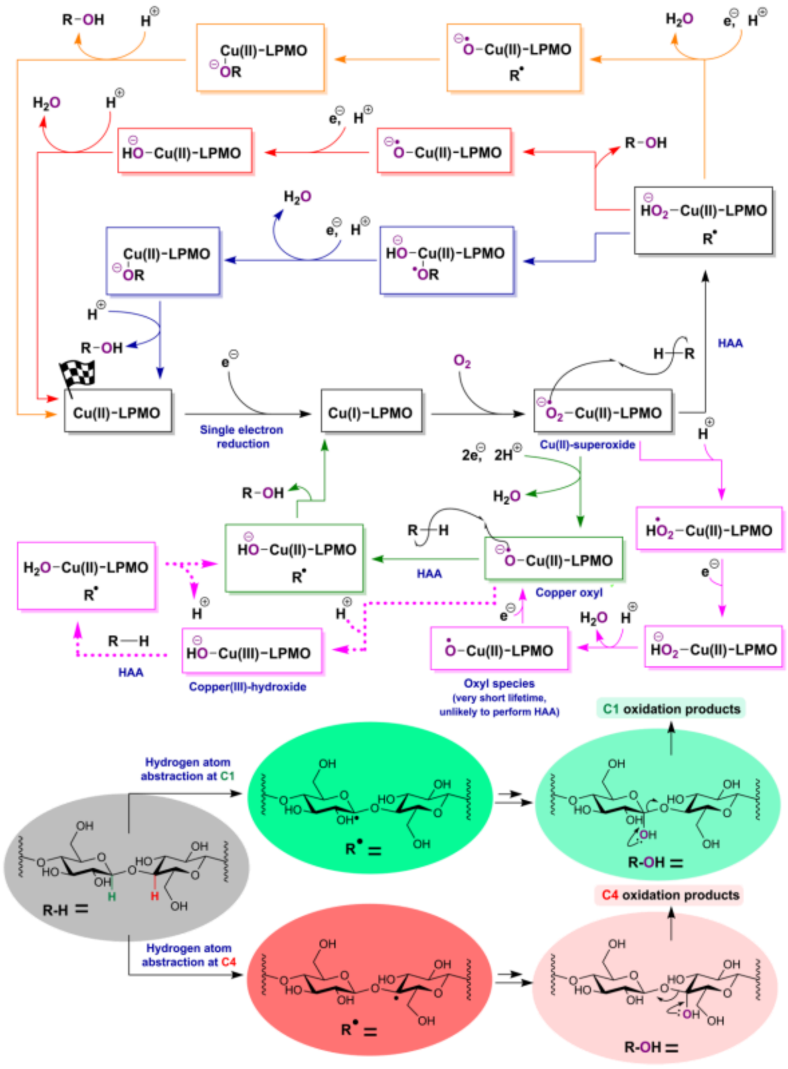

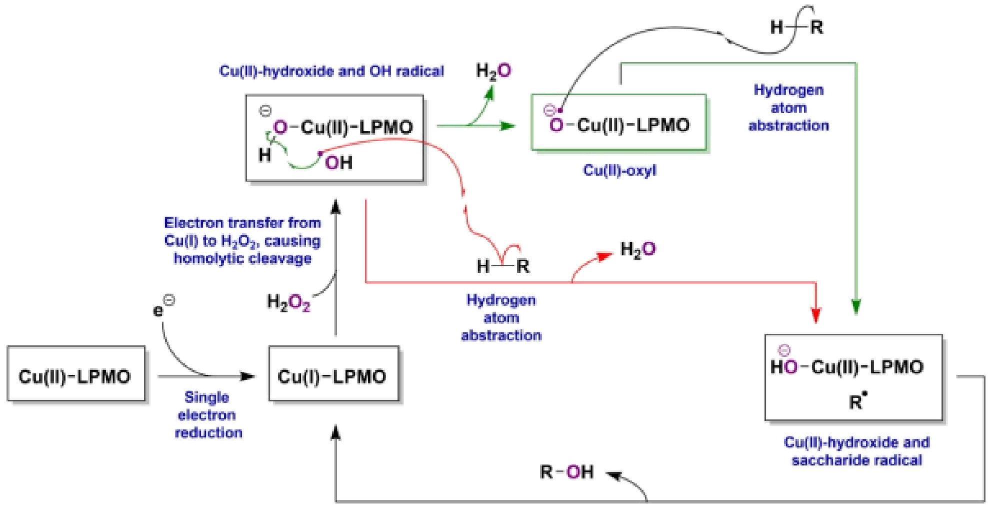

Figure S1 Putative mechanistic pathways for LPMO catalysed C-H bond oxidation via either (left) an oxygen co-substrate mediated pathway, or (right) a hydrogen peroxide co-substrate mediated pathway.

Further Experimental Methods for Protein Production and Biochemical Assays

Plasmid Generation

Plasmid Amplification

Samples of 6xHis tagged pET22-CjAA10BΔCBM and pET22-CfAA10 plasmid were amplified by transformation into 20 µL XL10-gold ultracompetent cells (Agilent) via heat shock, before plating on LB-Agar plates supplemented with ampicillin. A 5 mL LB culture was grown overnight for each plasmid before purification utilising a QIAprep Spin Miniprep Kit (Qiagen). The resultant plasmids were sequenced (Eurofins Genomics LightRun Tube Service) to confirm the presence of full-length inserts.

Strep®II-tagged AA10 Construct Generation

Strep®II-tagged AA10 inserts, produced by commercial gene synthesis, were purchased from GenScript. Primers were synthesised by Merck. The Polymerase Incomplete Primer Extension (PIPE) reactions was utilised to insert these genes into the pET22b vector replacing the respective his-tagged AA10. This was carried out utilising a modified protocol based on that outlined by Klock and Lesley,[1] with Q5 DNA polymerase and its respective reaction buffer (New England Biolabs) used in place of *Pfu* DNA polymerase. Heat shock, culture and minipreps were all carried out as described for the amplification of the AA10$_{C\text{-}His}$ plasmids, above.

AA10 Gene Expression

E. coli BL21(DE3) (New England Biolabs) was transformed by *Cj*AA10BΔCBM$_{C\text{-}His}$, *Cj*AA10BΔCBM$_{C\text{-}Strep}$ and *Cf*AA10 encoding plasmids via heat shock, before gene expression as described by Branch et al.[2] Cells were grown for 24 hours at 25 °C in 6-12 1 L cultures using 2xYT

media and 2L non-baffled flasks. Induction was achieved via addition of 1 mL of IPTG before incubation for a further 24 hours at 16 °C. Cells were then collected via centrifugation at 7000 xg for 30 mins using a Sorvall Lynx 4000 centrifuge (Thermo Scientific). The resulting pellets were frozen until required at -20°C.

His-tagged AA10 Protein Purification

His-Tagged *Cj*AA10BΔCBM was extracted via periplasmic lysis as outlined by Branch et al.[2] Purification of the resultant lysate was carried out with a three-step purification comprised of nickel affinity chromatography, anion exchange chromatography and gel filtration using AKTA protein purification systems. Nickel affinity chromatography was carried out with a 5 mL Histrap FF Crude column (Cytiva) as described by Branch et al.[2] Fractions containing AA10 were combined then spin concentrated to 1 mL utilising 10 kDa molecular weight (MW) cut off concentrators. The sample was subsequently diluted to 20 mL in anion exchange buffer A (50 mM Tris-HCl pH 8, 50 mM sodium chloride) and loaded onto a 1 mL HiTrap Q HP equilibrated in buffer A. The column was then washed with 5 column volumes (CV) of anion exchange buffer A and the LPMO was collected in the combined flow through and wash(confirmed via SDS-PAGE). The flow through and wash from anion exchange chromatography was concentrated via spin concentration (10 kDA molecular weight cutoff) to 1 mL before dilution to 20 mL with copper loading buffer (50 mM sodium phosphate pH 6, 150 mM sodium chloride). Aqueous $CuSO_4$ was added to the AA10 to yield a solution of 5:1 Molar ratio of copper:LPMO before spin concentration back to 1 mL. Size exclusion chromatography was then carried out with a S75 HILOAD 16/600 sephadex column (GE Healthcare). The resultant pure *Cj*AA10BΔCBM was then aliquoted, and flash frozen in liquid nitrogen for storage at -70°C.

Strep®II-tagged AA10 Protein Expression

Expression and periplasmic lysis of the strep tagged proteins was carried out as described for his-tagged variants. StrepII-tag affinity chromatography was carried out utilising an AKTA Pure protein purification system along with a StrepTrap HP 5 mL column (Cytiva). The periplasmic extract was loaded onto the column equilibrated in Strep Buffer A (100 mM Tris-HCl, 150 mM NaCl

pH 7.5 buffer). A 10 CV wash with buffer A was carried out before elution with 6 CVs of Strep Buffer B (100 mM Tris-HCl, 150 mM NaCl pH 7.5, 50 mM Biotin). The resulting fractions were analysed via SDS-PAGE to ensure purity. The resultant LPMO was concentrated to 1 mL before copper loading as for the His-tagged protein. The copper loaded protein was again concentrated to 1 mL and diluted to 10 mL with copper loading buffer. Following this wash the protein was concentrated to between 100 µM - 200 µM and flash frozen in aliquots before storage at -70°C.

Protein Concentration Assays

Protein concentration was monitored throughout the purification via microvolume absorbance at 280 nm with a Denovix DS-11 FX+ spectrophotometer. Final protein concentrations were confirmed post-purification using a Pierce™ Bradford Protein Assay Kit (Thermo Fisher) according to manufacturer's specifications. Absorbance at 595 nm was measured using a CLARIOstar Plus Plate reader (BMG Labtech) with a 96 well U bottom clear plate (Griener)

Biochemical Assays

2,6-Dimethoxyphenol Dye Assay

The ability of the LPMOs to reduce H_2O_2 as a substrate in solution was assayed by monitoring the concomitant oxidation of 2,6-dimethoxyphenol (2,6-DMP) to the dye molecule coerulignone. Assays at pH 7.5 were carried out as described by Breslmayr et al.[3] except for a scaledown in volume permitted by the use of a plate reader instead of a cuvette. Stock solutions containing both 1 mM 2,6-DMP, in 100 mM Tris pH 7.5 buffer, and either 0 or 200 µM H_2O_2 were produced and 98 µL of the 200 µM H_2O_2 was added to the first well of a row. 98 µL of the H_2O_2 containing and 98 µL H_2O_2 free stock were added to the next well. 98 µl of the resultant mixture was then combined with 98 µL of the H_2O_2 free stock in the next well. Subsequent 1:1 stepwise dilutions were repeated across the plate to yield a final range of H_2O_2 concentrations of 200 µM, 100 µM, 50 µM, 25 µM, 12.5 µM and 6.25 µM with a final H_2O_2 free control. Assays were initiated by addition of 2 µL 25 µM LPMO to each well, resulting in a final enzyme concentration of 0.5 µM LPMO. Absorbance was

monitored at 469 nm using a CLARIOstar Plus Plate reader (BMG Labtech). Reactions were carried out at 30°C in a 96 well U bottom plate (Greiner) and monitored continuously over 360 sec.

Amplex Red™ Assay

The ability of the LPMOs to reduce O_2 to H_2O_2 was monitored using an Amplex Red™ assay. Assay conditions were as described by Branch et al.[2] All assays were carried out in Amplex Red™ assay buffer (50 mM sodium phosphate pH 6). 50 µM Amplex Red™ reagent and 2 U mL^{-1} horseradish peroxidase type VI (HRP) (Sigma) were used in all experiments. The rate of resorufin formation was monitored in a black flat bottomed 96 well plate using a CLARIOstar Plus plate reader (BMG Labtech) with an excitation wavelength of 545 nm and an emission of 600 nm. LPMOs were assayed at a concentration of 2 µM and the concentration of the reductant ascorbic acid was adjusted to obtain Michaelis-Menten curves. Concentrations were calculated using a calibration curve of 1 - 5 µM H_2O_2, 50 µM Amplex Red™ reagent and 2 U mL^{-1} horseradish peroxidase.

Scanning Electron Microscopy

Micrographs of the electrode surface were obtained using a JEOL 7800F Prime Scanning Electron Microscope fitted with an off-axis Everhart-Thornley detector (Lower electron detector). Micrographs were obtained at 5 kV accelerating voltage and at 15 mm working distance with a range of magnifications.

Full Sequence Alignment Data

Presented in Figure S2 is the non-truncated version of the cutdown sequence alignment of AA10 LPMOs shown in Figure 1 of the main paper.

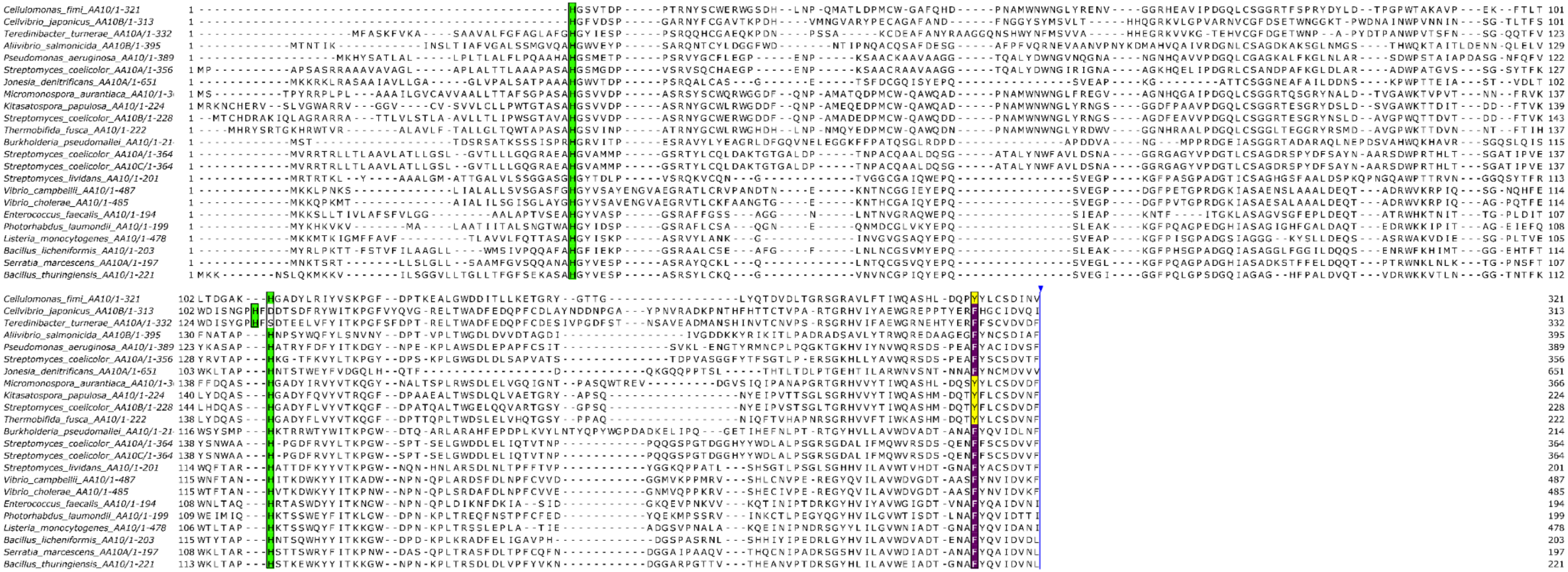

Figure S2 Full multiple sequence alignment of the LPMO domains of various structurally characterised AA10 LPMOs listed within the CAZY database (http://www.cazy.org/AA10_structure.html). Sequences were obtained from the Genbank entries (https://www.ncbi.nlm.nih.gov/) associated with the CAZY database entries. The secondary coordinating aromatic residues are highlighted in yellow or purple for tyrosine or phenylalanine, respectively. The active site histidine residues are shown in green. Numbering refers to the position within the multiple sequence alignment. The blue vertical line indicates where sections of residues corresponding to linker and carbohydrate binding domains have been hidden.

Non-Electrochemical Characterization Data

SDS-PAGE

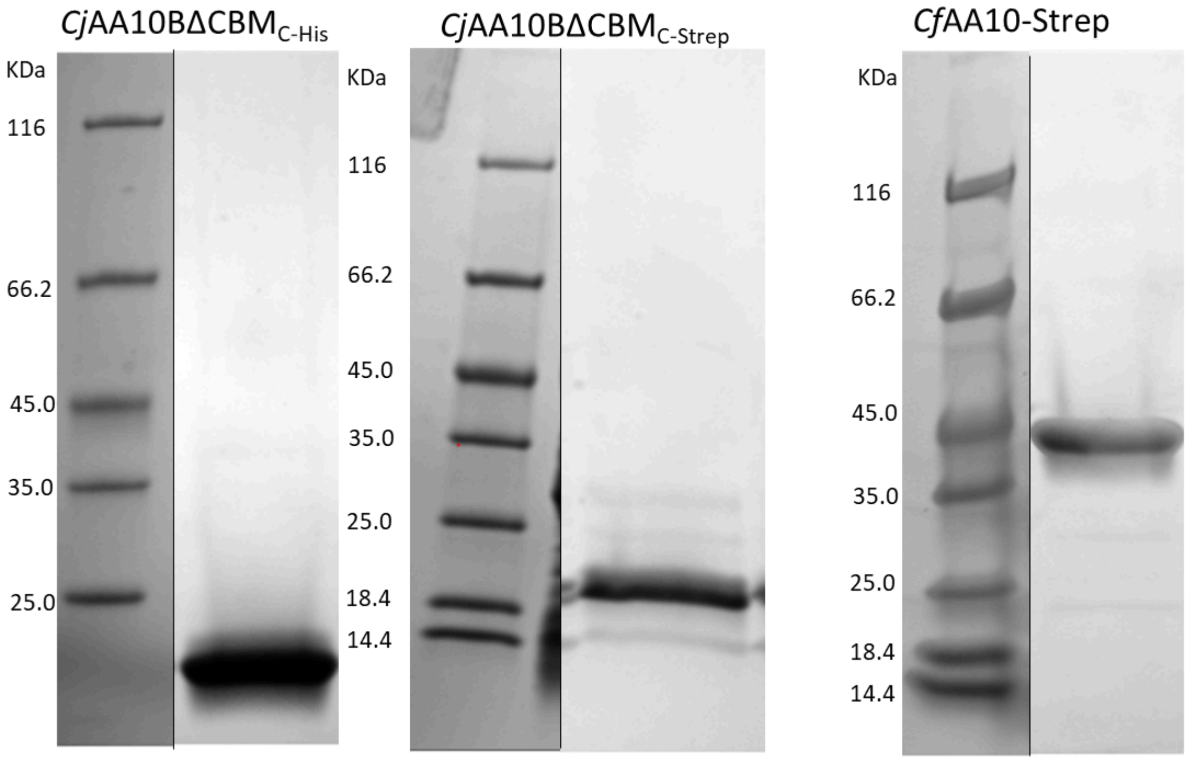

Figure S3 SDS-PAGE gels of concentrated AA10 samples used for experiments within the paper.

Solution Assay Data

Datasets for the 2,6-dimethoxyphenol (2,6-DMP) and Amplex Red^{TM} assays were obtained as described in the Materials and Methods section and analysed by fitting to the Michaelis-Menten equation using Origin. The red lines in Figure S4 and S5 depict the Michaelis-Menten fit of the obtained assay data (experimental data points shown by black squares). The Michaelis constant (K_M) and turnover rate (k_{cat}) parameters are reported in Table 2 of the main paper.

2,6-Dimethoxyphenol Assay Data

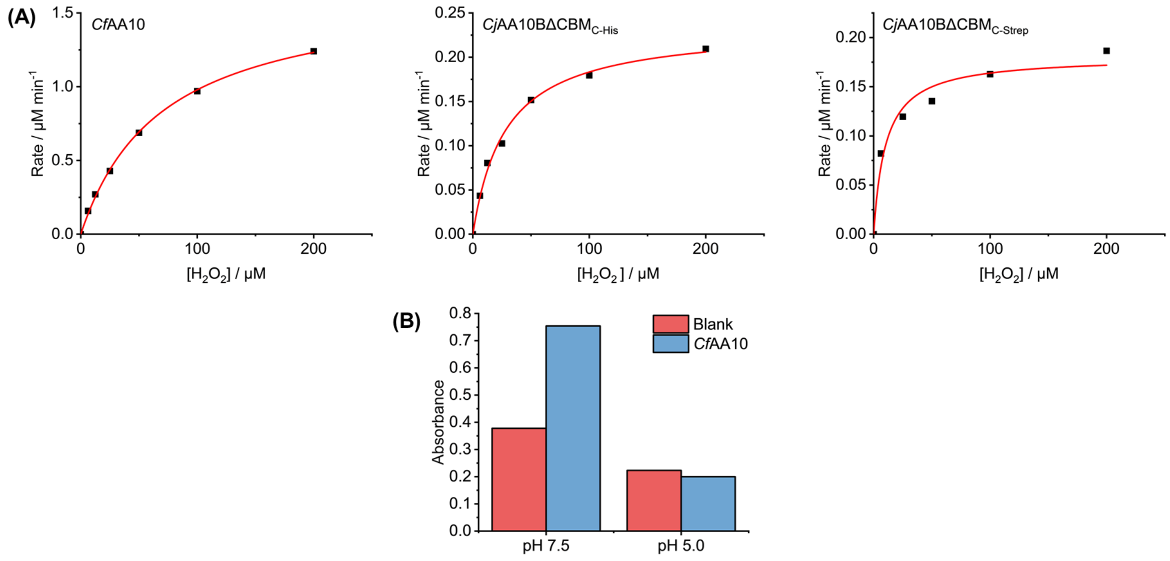

Figure S4 (A) 2,6-DMP peroxygenase assay of AA10 LPMOs. Michaelis-Menten curves comparing rate of reaction at various concentrations of H_2O_2 at pH 7.5, 30°C. (B) 2,6-DMP Dye assay pH controls. Endpoint 469 nm absorbance values after 25 mins for both enzyme free controls and *Cf*AA10 at pH 7.5 and pH 5.0

For comparison with the electrocatalytic H_2O_2 assays carried out at a range of pHs, the 2,6-DMP assay was repeated using *Cf*AA10, but enzymatic activity was not detected at pH 5, 35°C. This is summarised in Figure S4B which compares the endpoint absorbance after 25 mins of an LPMO-containing experiment at 200 μM H_2O_2, 100 mM sodium acetate pH 5.0 buffer at 35°C to an enzyme-free control. Comparable "endpoint" 200 μM H_2O_2 measurements made under pH 7.5 "standard assay" conditions (described in the Methods section of the main paper) validate that the experiments were conducted on an active enzyme sample.

Amplex Red™ Assay Data

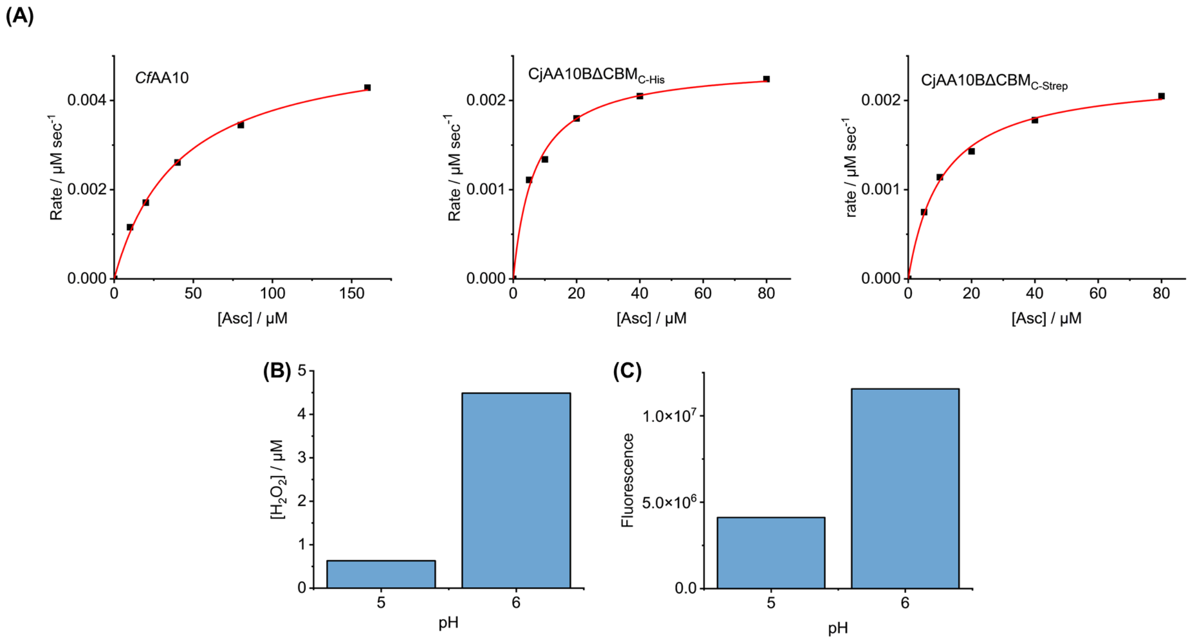

Figure S5 (A) Amplex red™ H_2O_2 production assay of AA10 LPMOs. Michaelis-Menten curve comparing the rate of H_2O_2 production compared to the concentration of the reductant ascorbic acid at pH 6 and room temperature. (B) Amplex Red™ H_2O_2 production assay pH control. Endpoint H_2O_2 concentration produced after 30 minutes at both pH 5 and pH 6 35°C by *Cf*AA10. Concentration values normalised against an equivalent enzyme free control reaction. (C) Amplex Red™ Horseradish peroxidase control. Fluorescence values obtained after incubating 1 µM H_2O_2 with 2 U mL^{-1} and 50 µM Amplex Red™ reagent in 50 mM pH 5 sodium acetate buffer and 50 mM pH 6 sodium phosphate buffer.

The Amplex Red™ H_2O_2 production assay was also repeated at pH 5.0 35 °C using the methodology outlined in the materials and methods section but substituting 50 mM sodium phosphate pH 6 buffer with 50 mM sodium acetate pH 5 buffer. Concentration values were calculated using a calibration curve produced using the aforementioned pH 6 buffer. As Figure S5B indicates, the Amplex Red™ assay indicates a substantial decrease in apparent concentration of H_2O_2 produced by CfAA10 after 30 minutes at pH 5 compared to pH 6. However, this was likely a consequence of changes in the fluorophore resorufin, which is known to perform poorly as a fluorophore below its pKa of pH ~6 as corroborated in Figure S5C.[4]

Table S1. *Cj*AA10BΔCBM$_{C\text{-}His}$ X-ray data collection and refinement statistics

**Data collection**	
Space group	$P2_1$
Cell dimensions	
a, *b*, *c* (Å)	76.8 74.9 137.5
α, β, γ (°)	90.0, 102.1, 90.0
Resolution (Å)	75.12 - 1.90 (1.93 - 1.90)
R_{merge}	0.077 (0.832)
R_{pim}	0.040 (0.452)
CC(1/2)	1.00 (0.95)
$I / \sigma I$	11.0 (1.4)
Completeness (%)	99.5 (98.5)
Multiplicity	4.6 (4.4)
**Refinement**	
Resolution (Å)	75.12-1.90
No. reflections (all/free)	118691/5863
R_{work} / R_{free}	0.230 / 0.276
B-factors (Å^2)	
Protein	27.6
Ion/solvent	35.2
Water	29.7
R.m.s. deviations	
Bond lengths (Å)	0.005
Bond angles (°)	1.262
PDB ID	9I1U

*Values in parentheses are for the highest-resolution shell.

Alphafold 3 Structural Prediction of Cf*AA10*

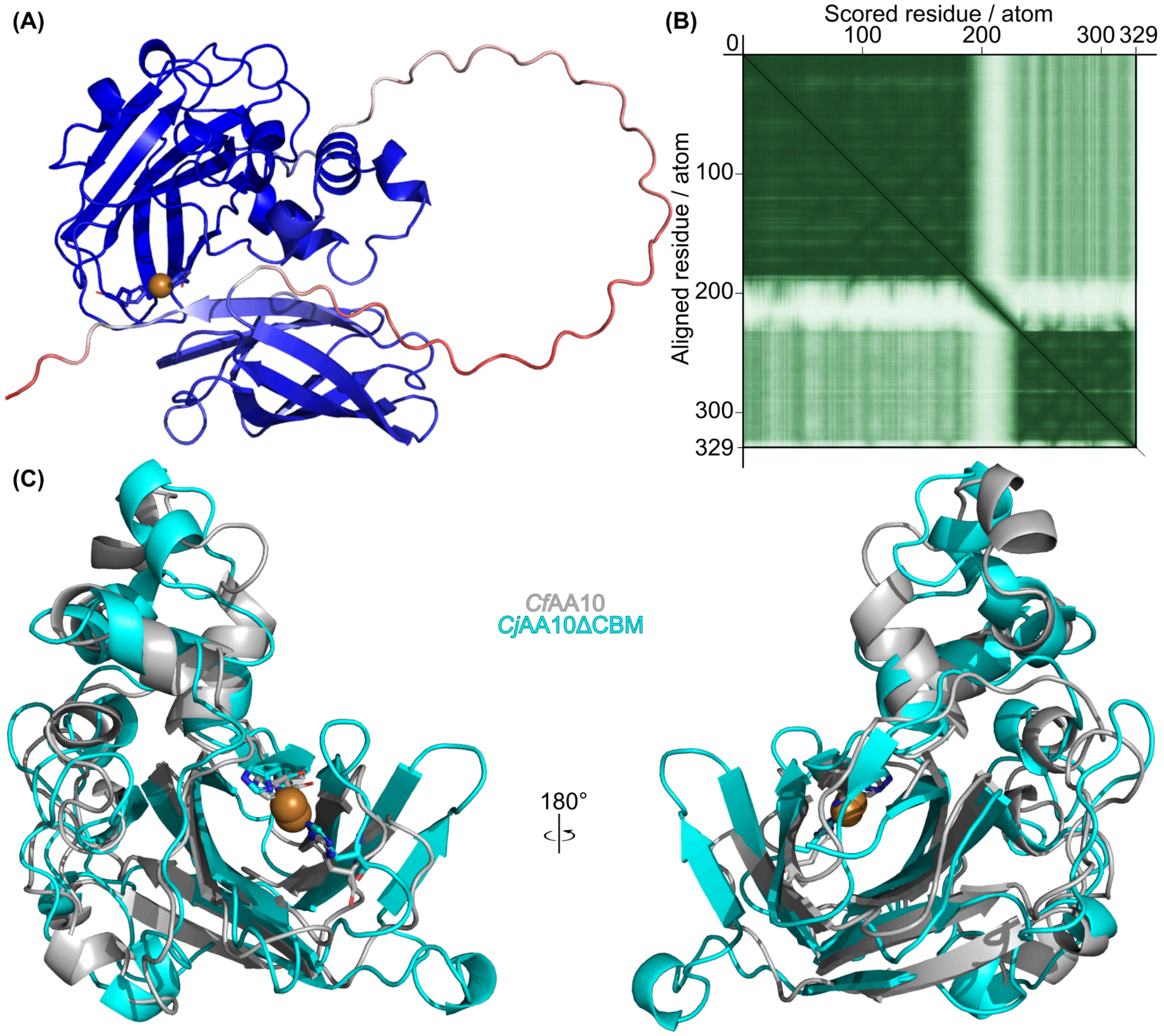

Figure S6 (A) Predicted structure of CfAA10 generated with Alphafold 3 using the AlphaFold Server, coloured by predicted Local Distance Difference Test (plDDT). Blue indicates high plDDT values, white middling values and red low values. (B) Predicted aligned error (PAE) matrix. Darker green indicates lower expected position error. (C) Structural overlay of the predicted structure of CfAA10 with the obtained crystal structure for CjAA10ΔCBM

Supplementary Electrochemical Data Analysing Reversible Copper Redox Chemistry at pH 5.0

Control Experiments Exploring the Stability of LPMO Films

To explore the stability of LPMO films on the PGE electrodes, two separate films of *Cj*AA10BΔCBM$_{\text{C-Strep}}$ were subjected to 6 DCV scans on either a stationary electrode, Figure S7A or rotating at 2000 rpm, Figure S7C. The maximum current values in the oxidative peaks, for both rotating and stationary electrodes, were extracted via baseline subtraction and plotted in Figure S7D. This experiment shows that the peak magnitude stabilises for both a stationary and rotating electrode, with the rotation of the electrode resulting in a more rapid loss of "multi-layer" characteristics.

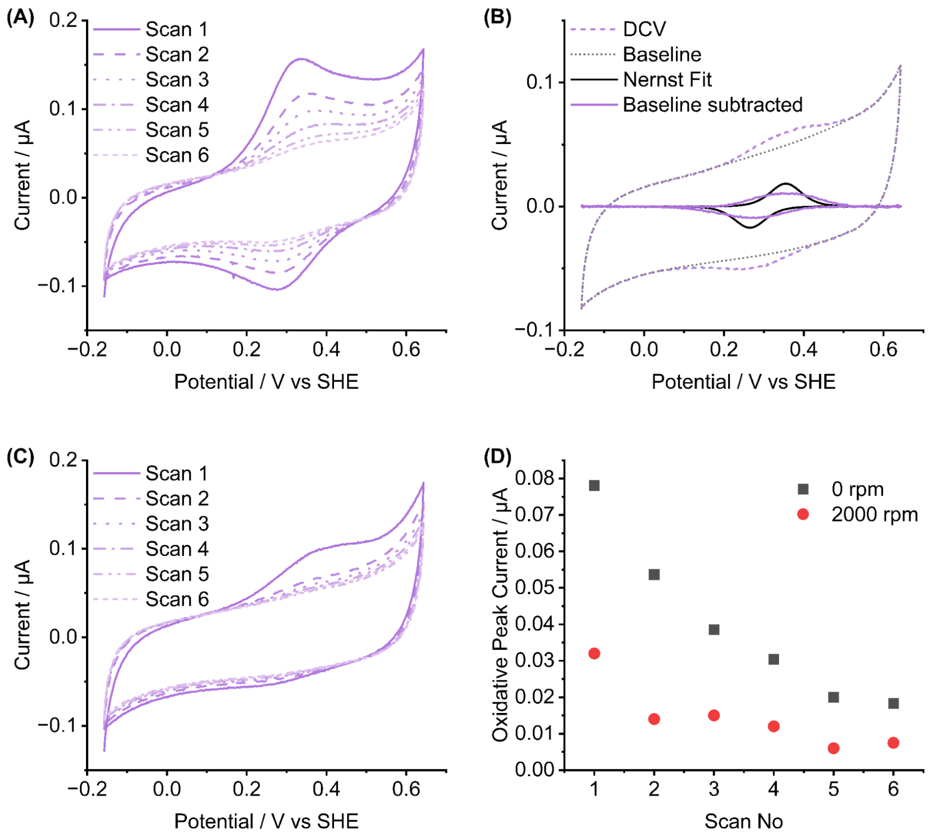

Figure S7 Comparison of direct current voltammetric measurements for *Cj*AA10BΔCBM$_{\text{C-Strep}}$ across a potential range from -160 mV to 640 vs SHE at 10 mV s^{-1} over 6 scans with (A) a stationary electrode, (C) a rotating electrode at 2000 rpm.(B) Comparison of extracted oxidative and reductive peaks, after baseline subtraction, to ideal peaks calculated from the Nernst equation for a single electron transfer. (D) Extracted maximum current for the oxidative peak of both rotating and stationary electrodes after baseline subtraction. All experiments were conducted in pH 5.0 buffer solution at 35 °C under an environment of N_2.

The oxidative and reductive peaks extracted from an "equilibrated" film (scan 6 in panel A) were then further analysed by comparing to a Nernst one-electron transfer model using the equation described by Heering et. al; and the results are shown in B.[5] The Nernst fit is generated using E_{ox} = 0.355 V vs SHE, E_{red} = 0.265 V vs SHE and a coverage of 2.0 pmol protein on the electrode.

Peak Integration of Cyclic Voltammograms

As described in the main paper, in order to extract the rate of enzyme catalysis, k_{cat}, the amount of LPMO adsorbed onto the surface of the electrode needs to be calculated. In order to do this a baseline is fit to both the oxidative and reductive peaks of substrate-free DCV experiments using the Peak Analyser tool on Origin as shown in red in Figure S8. The baseline can then be subtracted from the Faradaic current response, resulting in the baseline subtracted peak shown by the blue lines in Figure S8. The area under the baseline subtracted peak (shaded in light blue in Figure S8) is then integrated, again using Origin. The area under the peak is in units of μAmp Volts; accounting for the scan rate gives a value in Coulombs. Dividing by the Faraday constant, F, then allows for the calculation of the amount of enzyme attached to the surface of the electrode in moles.

In the case of the example shown in Figure S8, the peak area under the oxidative peak is integrated as 0.00846 μAV which can be divided by the scan rate of 10 mV s^{-1} resulting in a charge of 8.46 x 10^{-7} C, which is finally divided by F (96485.33 C mol^{-1}), giving a surface coverage of 8.77 x 10^{-12} mol LPMO on the electrode. The same process was carried out for the reductive peak with the area under the peak being integrated as 0.00935 μAV which gives a surface coverage value of 9.69 x 10^{-12} mol. The average surface coverage (Γ x A in Equation 2 of the main paper) is then calculated as 9.23 x 10^{-12} mol.

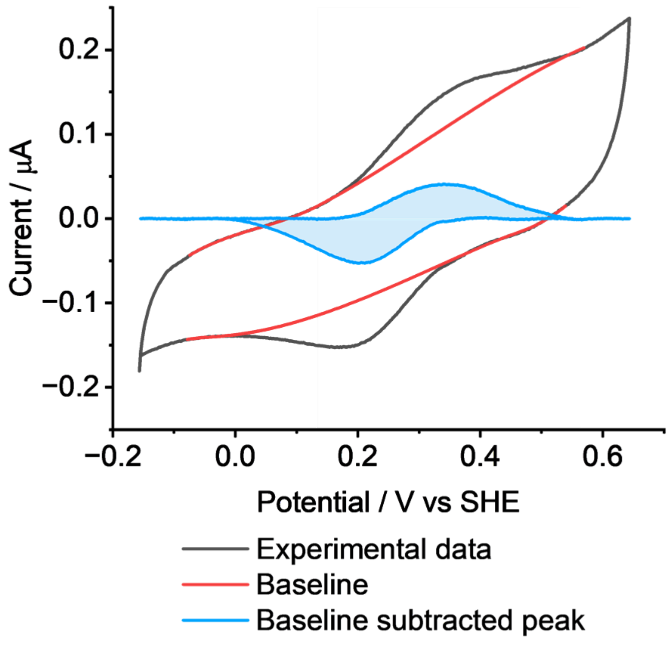

Figure S8 Example of peak analysis of a 10 mV s^{-1} CV taken of *Cf*AA10 across a potential range of -160 to 640 mV at pH 5.0 and 35 °C. The experimental data is shown in grey, with the baselines fitted below both peaks shown in red. The baseline subtracted peaks are shown in blue and the area below each peak is shown shaded in light blue.

Surface-bound Flavin Control

Another experiment to investigate the electrode surface is shown in Figure S9, in which 10 μL, 400 μM flavin adenine dinucleotide (FAD), a redox active coenzyme, is applied to the surface of the electrode and left to adsorb for approximately 30 min. The electrode tip was then rinsed with MilliQ water before the working electrode was placed into an electrochemical cell containing 15 mL of FAD-free buffer. We are certain that FAD formed a surface bound species as the signals remain stable despite rotation of 2000 rpm. A DCV experiment was performed at 10 mV s^{-1}, a slow scan

rate was chosen to ensure complete oxidation/reduction under the kinetics of the experiment. The areas under the oxidative and reductive peaks were integrated and averaged to determine the coverage of FAD on the electrode as 29 pmol, as shown in Figure S9 (FAD undergoes reversible two-electron redox chemistry).

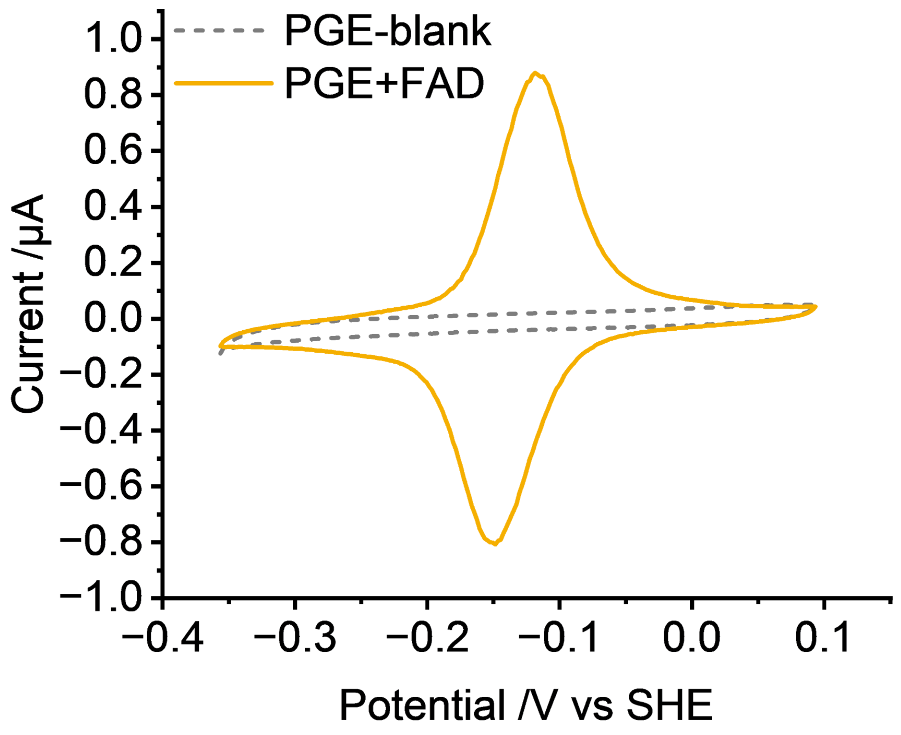

Figure S9: The 6th scan of a DCV experiment in which FAD was adsorbed onto the surface of the working electrode. Scans were taken from -340 to 90 mV vs SHE, 10 mV s^{-1}, pH 5.0, 2000 rpm.

Working Electrode Surface Characterisation

Scanning Electron Microscopy (SEM)

As mentioned in the experimental section, scanning electron microscopy (SEM) was performed to evaluate the surface of the working electrode. The micrographs show that the surface of the PGE electrode is extremely rough and uneven, corresponding with similar micrographs produced by Blanford and Armstrong in which nitrogen porosimetry experiments proved that the real surface area of edge pyrolytic graphite can be magnitudes larger than the geometric surface area.[6]

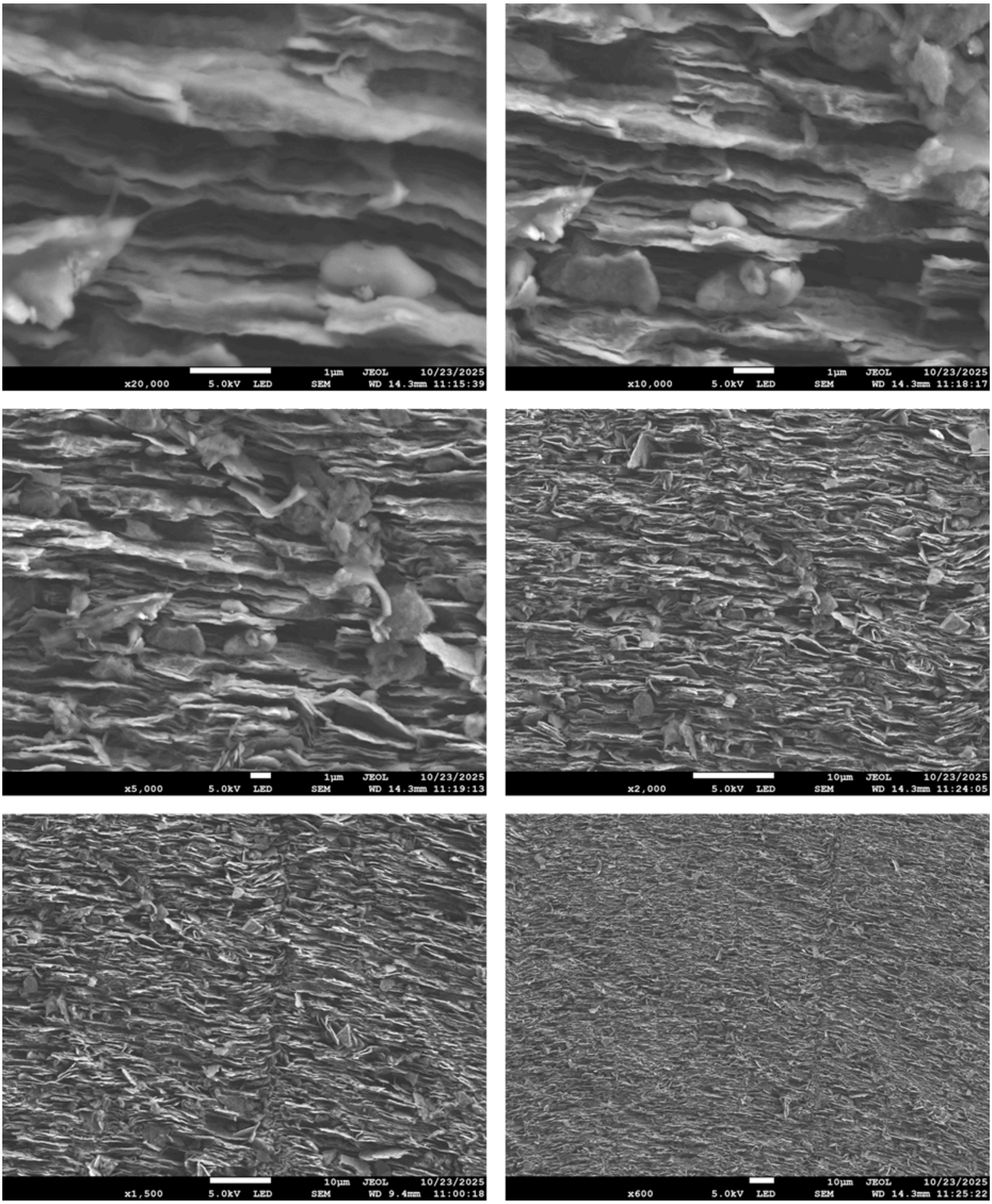

Figure S10: Scanning electron micrographs of the PGE surface of the working electrode after abrasion with P1200 sandpaper and rinsing with water at varying magnification (top left: x20,000; top right: x10,000; middle left: x5,000; middle right: x2,000; bottom left: x1,500; bottom right: x600).

Control Experiments on Free Cu and Apo-LPMO

To further confirm that the observed signals originate from copper-containing LPMO and not free copper, in-situ copper loading on the electrode was carried out. As-isolated *Cj*AA10BΔCBM$_{C\text{-}Strep}$ which had not been copper loaded was drop-cast onto the electrode and then the film was further

treated with 1 mM EDTA (adjusted to pH 7.0) for 5 min (electrode tip immersed in ~1.5 mL EDTA solution) to ensure complete metal removal. This so-called "Apo-*Cj*AA10BΔCBM$_{C\text{-}Strep}$" was then electrochemically interrogated as shown in Figure S11A, confirming a response that is indistinguishable from a protein free electrode. The Apo-*Cj*AA10BΔCBM$_{C\text{-}Strep}$ film was then copper loaded by submersion of the electrode in 200 μM aqueous $CuSO_4$ for 5 min to generate so-called "Holo-*Cj*AA10BΔCBM$_{C\text{-}Strep}$". The electrode tip was then rinsed thoroughly and the enzyme film was scanned until the peak magnitude stabilised (Figure S11B). Notably, the film equilibrates over time to give oxidative and reductive peaks of equivalent size, meaning that at this slow scan rate (10 mV s^{-1}) the $Cu^{2+/1+}$ oxidation state change appears fully reversible. Treatment of the Holo-*Cj*AA10BΔCBM$_{C\text{-}Strep}$ film with 1 mM EDTA for 5 min, after the electrochemical cell was cleaned and the buffer exchanged, was insufficient to completely remove the observed signal (Figure S11C). However, a further 5 min EDTA treatment removed the signal entirely, which could then be recapitulated with a second copper loading. Notably the signal did not diminish greatly despite several vigorous washing steps. Immersion of a blank PGE electrode with no LPMO film in $CuSO_4$ under the same "copper-loading" conditions did not result in a notable signal (Figure S11D)

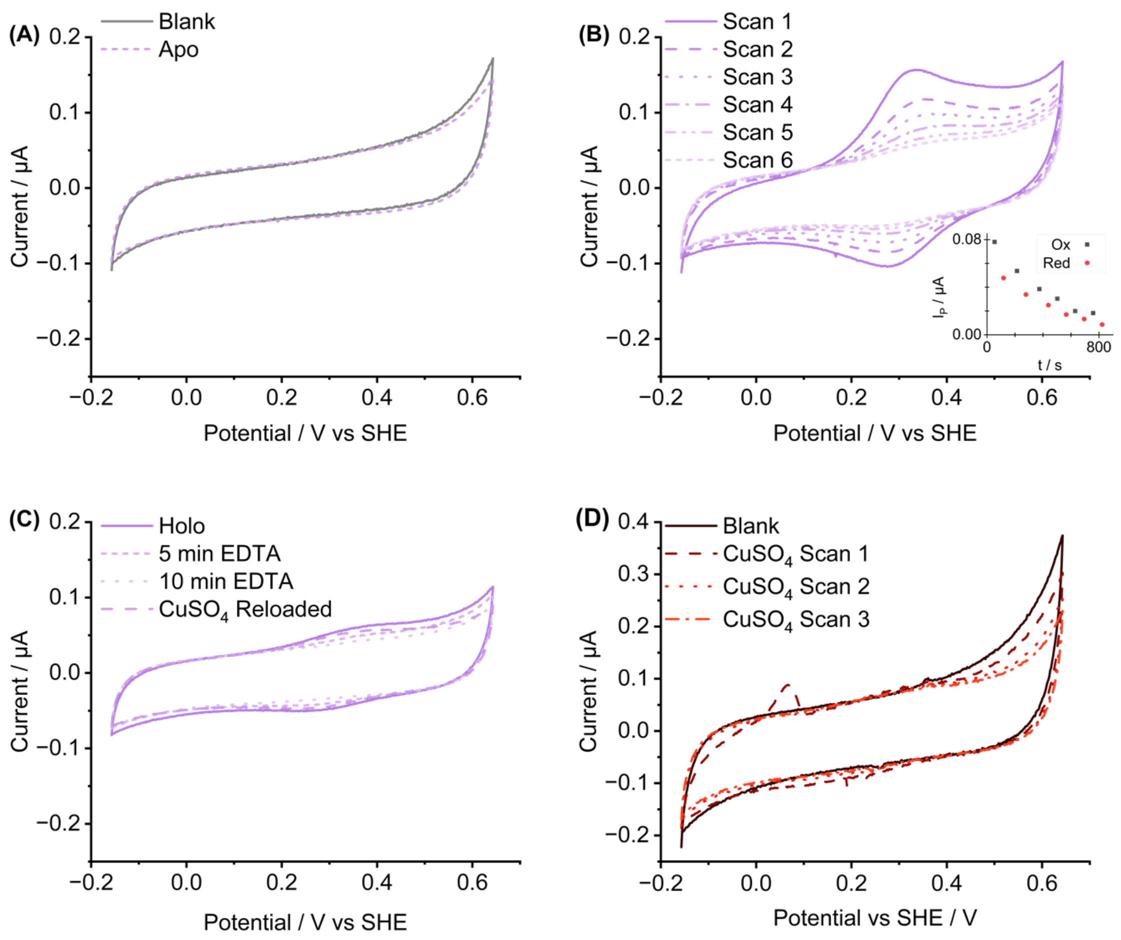

Figure S11 Direct current voltammetric measurements for *Cj*AA10BΔCBM$_{C\text{-}Strep}$ or $CuSO_4$ across a potential range from -160 mV to 640 mV vs SHE at 10 mV s^{-1}. (A) Comparison of a blank PGE electrode with a film of Apo-*Cj*AA10BΔCBM$_{C\text{-}Strep}$ after submerging the electrode in 1mM EDTA for 5 mins. (B) DCV measurements of the same film after submerging the electrode in 200 μM $CuSO_4$ for 5 minutes, scans were repeated until stable film was reached. The inset graph compares absolute peak magnitude (baseline subtracted) against time in seconds for both the oxidative and reductive peaks.(C) DCV measurements comparing the stable holo-LPMO film after submerging the electrode in 1mM EDTA for 5 and 10 minutes respectively. The resulting film was then submerged in 200 μM $CuSO_4$ for 5 minutes again as indicated by the $CuSO_4$ reloaded scan. (D) DCVs of a blank PGE Electrode before and after submersion in 200 μM $CuSO_4$ for 5 minutes.

A second control experiment was performed to investigate the effect of adding free copper to the system and whether this produces any electrocatalytic response. Figure S12A shows the CVs taken before and after the addition of 10 µL of 250 µM $CuSO_4$ solution, the amount of copper that would be released into solution if all of the active site copper from an LPMO film was freed from the enzyme structure. The resulting CVs show no large changes, with no peak-like features appearing upon the addition of $CuSO_4$. Figure S12B shows an experiment in which H_2O_2 is injected into the electrochemical cell resulting in a final bulk concentration of ~10 mM H_2O_2. This experiment is repeated for a bare electrode in the presence and absence of $CuSO_4$, showing that the current response remains consistent with and without $CuSO_4$.

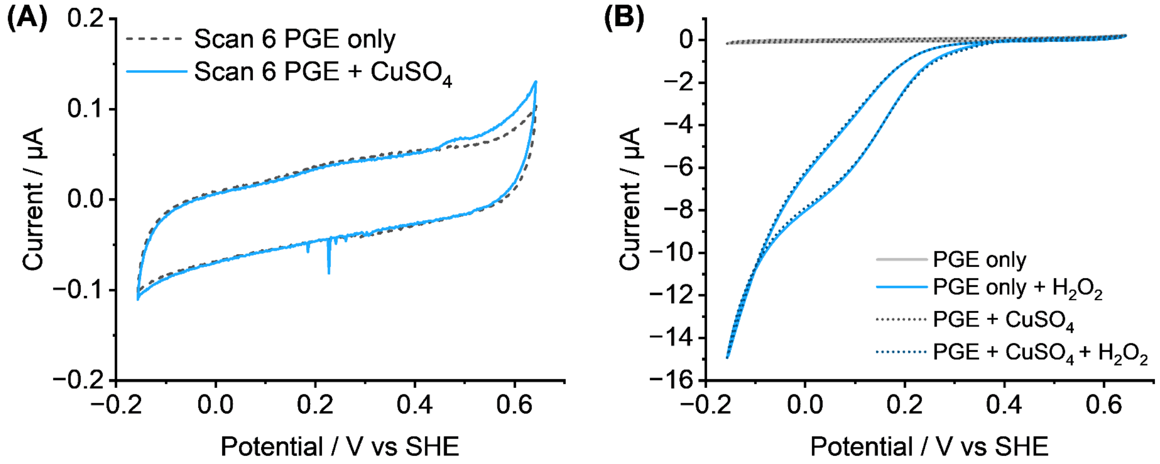

Figure S12: (A) The 6th cycle of a CV experiment for a bare electrode in the presence (solid blue line) and absence (dotted grey line) of 10 µL, 250 µM $CuSO_4$. Scans were taken from -160 to 640 mV vs SHE at 10 mV s^{-1}, pH 5.0, 35°C, 2000 rpm. (B) Continuous cycling CV experiment (scans 3 and 10 shown) in which H_2O_2 (10 µL, 250 µM) is injected after the third scan and the resulting current is recorded. Scans taken from -160 to 640 mV vs SHE at 10 mV s^{-1}, pH 5.0, 35°C, 2000 rpm.

Figure S13 shows a final copper control experiment in which a continuous cycling DCV experiment was performed with a bare PGE working electrode. Figure S13A shows the current response of six scans in a solution containing 250 µM $CuSO_4$ in buffer solution (20 mM Na acetate, 20 mM Na phosphate, 500 mM Na sulfate) at 100 mV s^{-1}. After cycling in $CuSO_4$ solution, the working electrode was rinsed with water and the solution was replaced with fresh buffer solution containing no $CuSO_4$, six DCV scans were then performed at 10 mV s^{-1}. Figure S13B shows the results of this experiment with a prominent sharp peak-like feature seen at ~150 mV vs SHE, along with a redox couple with large peak-to-peak separation. Figure S13C shows an overlay of the sixth scan of the experiment shown in Figure S13B with a CV taken of *Cf*AA10 LPMO adsorbed on the electrode. This overlay shows a sample of LPMO on the electrode surface gives a different response when compared to the bare electrode that has been cycled in $CuSO_4$, indicating that the LPMO signal relates to correctly folded protein adsorbed on the electrode surface and not free copper.

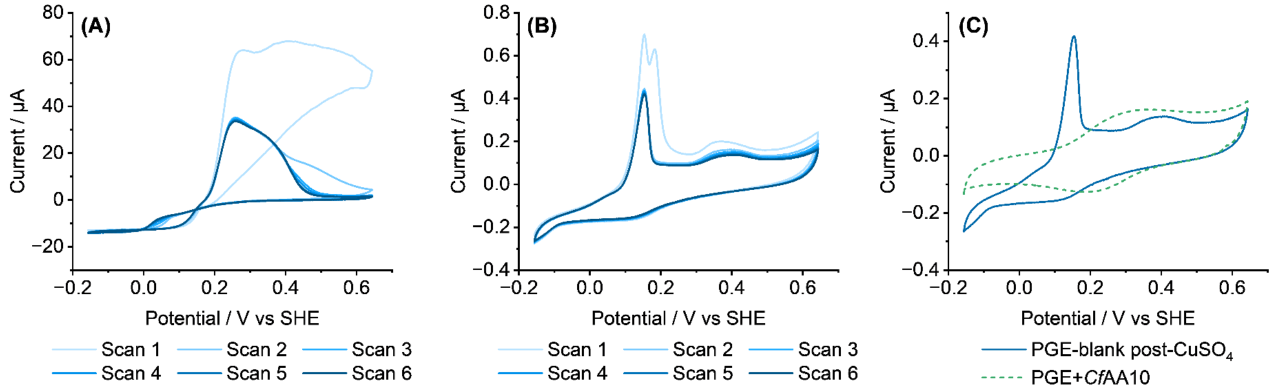

Figure S13: CVs in which of (A) bare PGE electrode in 250 µM $CuSO_4$ solution (100 mV s^{-1}) (B) PGE electrode in fresh buffer solution after six cycles in 250 µM $CuSO_4$ solution (10 mV s^{-1}) (C) overlay of the sixth scan from Figure S13B (solid dark blue line) with a DCV of *Cf*AA10 (dashed green line) (10 mV s^{-1}). All scans were performed from -160 to 640 mV vs SHE, pH 5.0, 35 °C, 2000 rpm.

Control Experiments Showing that the Substrate-free Voltammetric response of CjAA10BΔCBM is Unaffected by the Identity of the Purification Tag

Figure S14, shown below, demonstrates the lack of impact of purification strategy on the redox activity of the *Cj*AA10BΔCBM enzyme. Figure S14A shows an overlay of CVs taken of *Cj*AA10BΔCBM$_{C\text{-}His}$ and *Cj*AA10BΔCBM$_{C\text{-}Strep}$, in which the redox peaks appear at the same potentials for both constructs. Figures S14A and S14B show equivalent SWV experiments from which midpoint potentials were calculated as 238 mV and 231 mV vs SHE for the His- and Strep-constructs respectively.

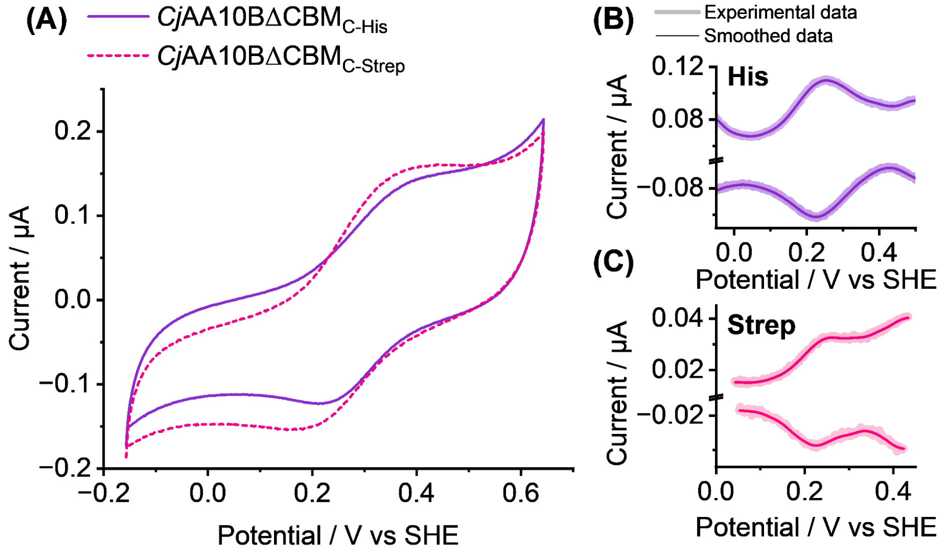

Figure S14 Comparison of (A) direct current voltammetric measurements for *Cj*AA10BΔCBM$_{C\text{-}His}$ (purple line) and *Cj*AA10BΔCBM$_{C\text{-}Strep}$ (pink line) constructs across a potential range from either -160 mV to 640 vs SHE at 10 mV s^{-1}. (B) SWV experiments for *Cj*AA10BΔCBM$_{C\text{-}His}$ taken across a potential range from -50 to 500 mV vs SHE with a 10 mV pulse amplitude and a 2 Hz frequency. (C) SWV experiments for *Cj*AA10BΔCBM$_{C\text{-}Strep}$ taken across a potential range from 50 to 420 mV vs SHE with a 10 mV pulse amplitude and a 2 Hz frequency. All experiments were conducted in pH 5.0 buffer solution at 35 °C under an environment of N_2.

experiments.

Temperature impact on non-catalytic electrochemistry

Electrochemical experiments showing the impact of temperature on signal intensity of the non-catalytic $Cu^{2+/1+}$ transition are shown in Figure S15. Both DCV and SWV scans were recorded at 5 °C, 20 °C and 35 °C for *Cf*AA10 in pH 5.0 buffer solution. All experiments were performed on the same electrode film, with the first scan taken at 5 °C. The temperature was then increased to 20 °C before a second set of DCV and SWV scans were performed. The temperature was again increased to 35 °C and the electrochemistry was repeated.

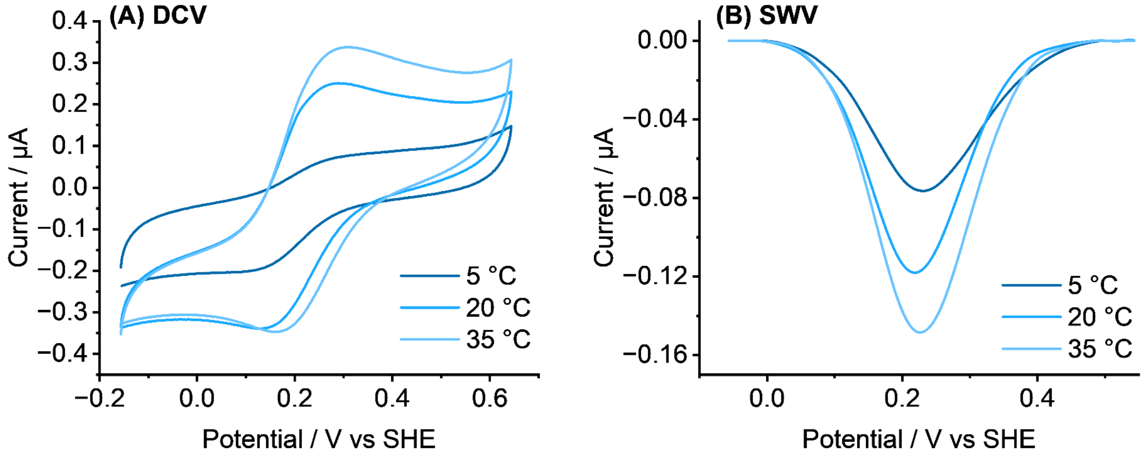

Figure S15 Voltammograms of *Cf*AA10, recorded using (A) DCV (cyclic scanning) from -160 → 640 mV vs SHE at 10 mV s^{-1}, (B) SWV (individual oxidative and reductive sweeps) from 540 → -60 mV vs SHE at 2 Hz, 10 mV pulse amplitude. Scans were performed on the same protein film of *Cf*AA10 LPMO. The first scan was performed at 5 °C before the temperature was increased to 20 °C and then finally 35 °C. All experiments were carried out in a pH 5.0 buffer solution of 20 mM sodium acetate, 20 mM sodium phosphate and 500 mM sodium sulfate under an atmosphere of N_2.

Analysis of Square Wave Voltammetry Data

Analysis of the pH 4 SWV data was performed for both AA10 LPMOs in order to extract the rate constant for the $Cu^{1+/2+}$ transition. The extraction of the "net" current was undertaken as is standard in the literature.[7] In all cases, the Faradaic dimensionless current Ψ at dimensionless time m is modelled using the recursive relationship in Equation S1, with parameters: $Cu^{2+/1+}$ reduction potential, E_{rev}; electron transfer rate, k_0; charge-transfer coefficient, α and f_s is the sampling frequency of the square wave. The current I is non-dimensionalised using the constant $1/(FA\Gamma\omega)$, where F is Faraday's constant, A is the surface area of the electrode, Γ is the LPMO surface coverage and \omega is the square wave frequency. The potential values (E_{app} and E_{rev}) are non-dimensionalised using F/RT, where T is the temperature and R is the ideal gas constant. The dimensionless electron transfer rate k_0 is obtained from k_{et} using the dimensional constant $1/\omega$.

Figure 6 of the main paper shows the results of using a cubic fit to account for the non-Faradaic "background" current. The formulae for the background polynomial are shown in Equation S2. with I_b, I_b^1, I_b^2 and I_b^3 all being nondimensional values. Estimates for the best-fit parameter values were obtained by using the CMAES algorithm to maximise a likelihood function comparing a single scan of square-wave current data, assuming independent and identically distributed noise. Whilst this approach accounts for the magnitude of the background contribution, it does not model the impact of Ohmic drop (i.e. iRu) on the Faradaic process. It should be noted that in other work using

differential equation models we have determined that these effects can have strong impacts on the final parameter estimates.

The analysis described above was performed to emphasise the sluggishness of the electron transfer at the copper centre in the absence of H_2O_2/O_2 and not to derive any specific conclusions in relation to the specific electron transfer mechanism between the copper centre and the electrode. Regardless of mechanism, under the experimental parameters described in the main manuscript the absolute upper bound for the kinetic rate constant to observe Faradaic signals is 20 s^{-1}, and thus even a more complex mechanism of electron transfer would still result in rates <20 s^{-1}.

Equation S1

$$\Psi'_m = \frac{k_0 e^{\alpha(E_{appm} - E_{rev})}\left(1 - \frac{1 + e^{-(E_{appm} - E_{rev})}}{f_s}\sum_{j=1}^{m-1}\Psi_j\right)}{1 + \frac{k_0 e^{\alpha(E_{appm} - E_{rev})}}{f_s}\left(1 + e^{-(E_{appm} - E_{rev})}\right)}$$

Equation S2

$$I_{b_{tot_m}} = I_b\left(1 + I_b^1 E_{appm} + I_b^2 E_{appm}^2 + I_b^3 E_{appm}^3\right)$$

For comparison with the SWV kinetic analysis of electron transfer, DCV experiments were performed at scan rates ranging from 1 mV s^{-1} to 100 mV s^{-1}. Figure S16A shows an overlay of a dataset obtained using *Cj*AA10BΔCBM$_{C\text{-}His}$. The current is normalised by dividing by the scan rate, *v*, of the experiment; as the capacitive current increases linearly with *v* this allows for the presentation of the data on the same scale. Equivalent data was recorded using a *Cf*AA10-coated working electrode. It should be noted that due to the long-term instability of the protein films, the DCV data was collected using multiple protein films and thus the magnitude of the signals at different scan rates cannot be quantitatively compared.

For an idealised one-electron redox system, the potential at which the oxidative and reductive peaks are recorded diverge away from the reversible potential E_{rev} once the scan rate is fast enough that the reaction is no longer in the equilibrium regime. However, in the data presented in Figure S16A, it can be seen that as the scan rate is increased, the LPMO signal becomes broader and less defined, before disappearing almost completely by 100 mV s^{-1}. So-called "trumpet plots", as shown in Figure S16B and 16C, plot peak potential against the logarithm of the scan rate. In the LPMO experimental data, the potential of the reductive peak (hollow points) does not decrease with scan rate. For this reason, it is unlikely that an estimate of k_{et} obtained from fitting to the trumpet plot data would be useful. Instead, we use the trumpet plot data as a sense check of the parameters obtained from the square wave analysis. Forward simulation of trumpet plots, using k_{et} values extracted from SWV analysis, is shown by the orange lines in Figure S16B, with the steepest and shallowest lines marking the expected change in peak potential with scan rate when using a maximum and minimum value for k_{et}, respectively, and E_{rev} and constant peak separation estimated from the DCV data. The experimental data for the oxidation peak potentials, shown as solid blue points, fits this analysis well, validating the SWV analysis.

Figure S16 shows how the redox signal changes with scan rate for DCV experiments. As mentioned above, the signal is quickly outpaced by scan rate, losing definition and resulting in broad peaks and atypical responses of the peaks with increasing scan rate, i.e. an unexpected lack of shift in peak position with scan rate.This is compounded by the use of PGE electrodes which are known to result in persistent peak-broadening.[8] The low ratio of Faradaic signal to the capacitance

may be responsible for limiting the accuracy when extracting information. As above, we therefore conclude that this method is unsuitable for analysis of the kinetics of electron transfer and thus we have instead utilised SWV.

Figure S16C shows a further set of trumpet plot simulations overlaid on the same experimental LPMO data. These confirm that altering the value of the uncompensated resistance (a key parameter in trumpet plot simulations which cannot be estimated from the squarewave analysis) while $k_{et,}$ is held constant at 0.41 and 0.23 s^{-1} for the Cj and Cf cases respectively, does not have a large impact on the sensitivity of the peak potentials to scan rate, even over several orders of magnitude.

(A)

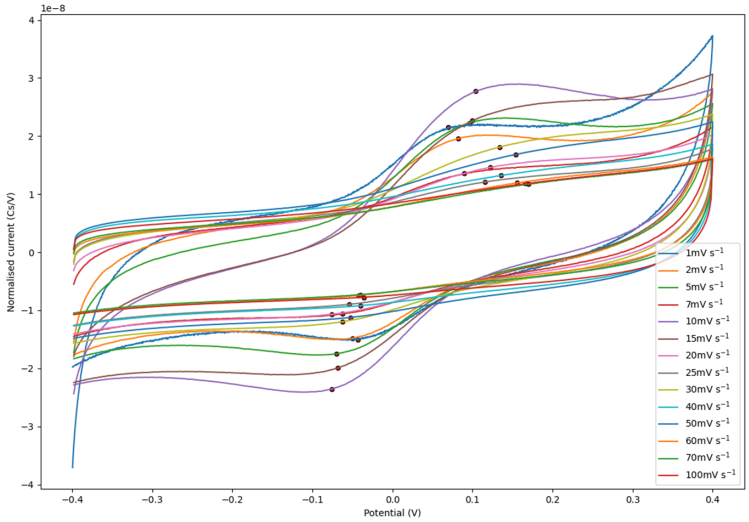

(B)

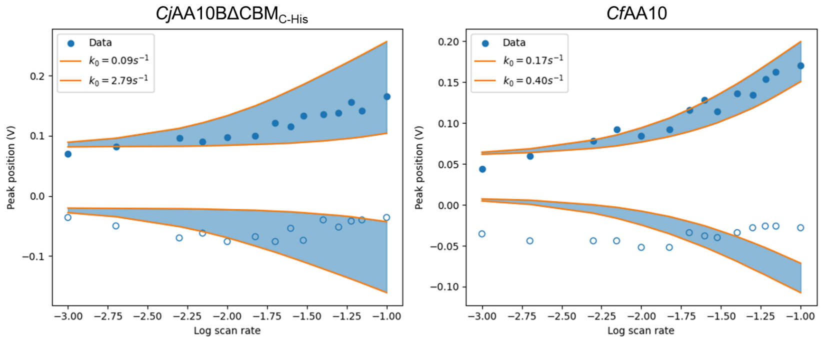

(C)

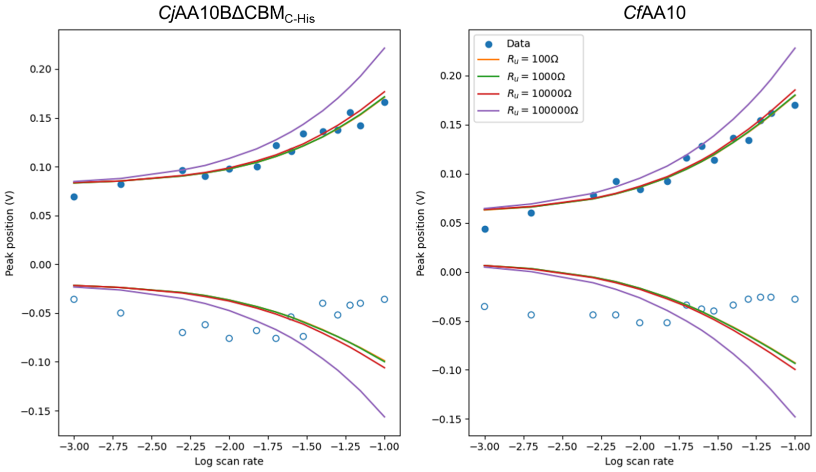

Figure S16 (A) Data from *Cj*AA10BΔCBM$_{\text{C-His}}$ dcV experiments at a range of scan rates, as indicated by the legend. The current is normalised by dividing by the scan rate of the experiment. Experimental conditions: pH 4.0 20 mM sodium acetate, 20 mM sodium phosphate, 500 mM sodium sulfate buffer solution at 35 °C. (B) Overlay of (left) *Cj*AA10BΔCBM$_{\text{C-His}}$ and (right) *Cf*AA10 pH 4.0 trumpet plot data points (solid blue circles indicate oxidative peak positions, hollow blue circles show reductive peak positions) and trumpet plot simulations generated using the maximum (steepest orange lines) and minimum (shallowest orange lines) values for k_{et} that were extracted from the SWV modelling. (C) Impact of resistance on the outcome of trumpet plots of *Cj*AA10BΔCBM$_{\text{C-His}}$ and *Cf*AA10.

Supplementary Electrocatalysis Data

Hydrogen Peroxide LSV at pH 5.0

Linear sweep voltammetry (LSV), see Figure S17, was employed alongside chronoamperometry to investigate the relationship between H_2O_2 concentration and enzyme activity. LSV measurements were taken for both holo-*Cf*AA10 and holo-*Cj*AA10BΔCBM in the absence of H_2O_2. H_2O_2 solution was then injected and LSV was repeated at 5 mM H_2O_2 and 10 mM H_2O_2. The enzyme was then incubated in EDTA to remove the active site copper atom, rendering the protein sample inactive. The same LSV experiments were then repeated on the apo-enzyme (both apo-*Cf*AA10 and apo-*Cj*AA10BΔCBM). Comparison between the apo and holo enzyme shows that the holo enzyme produces a larger reductive current response. This indicates that the active holo form of the LPMO catalyses H_2O_2 reduction more energetically efficiently than the inactive apo-enzyme electrode.

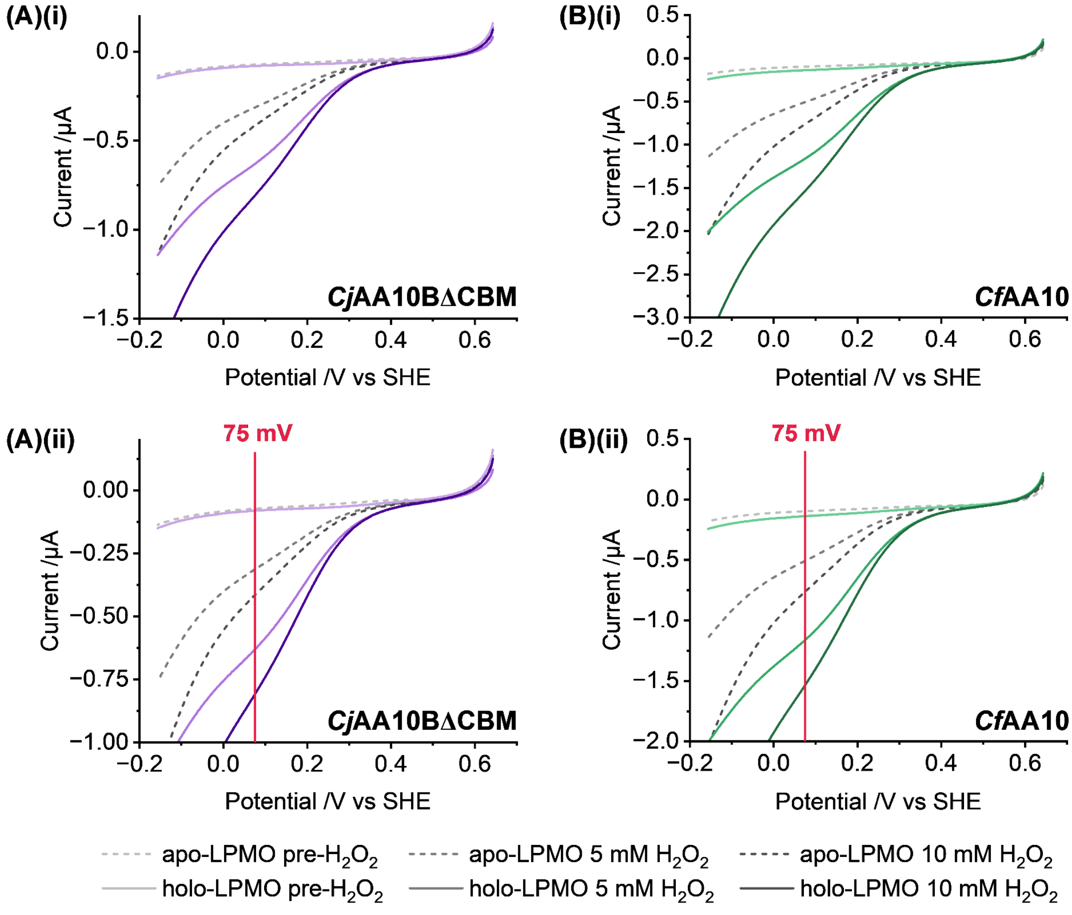

Figure S17 Voltammetric data taken of (A) *Cj*AA10BΔCBM$_{C\text{-}His}$ and (B) *Cf*AA10, pre (lighter lines) and post (darker lines) addition of H_2O_2. holo-LPMO are shown with solid lines whilst apo-LPMO controls are represented with dashed lines. Measurements were collected across a potential range of 640 to -160 mV vs SHE at 10 mV s^{-1} in pH 5.0 buffer solution at 35 °C with the electrode rotator set to 2000 rpm. The vertical red lines at 75 mV denote the constant voltage employed in the chronoamperometry experiments reported in the main paper. (A)(ii) and (B)(ii) are zoomed in versions of (A)(i) and (B)(i), allowing for clearer visualisation of the differences between apo- and holo-LPMO.

Dioxygen LSV at pH 5.0

Figure S18 shows LSV measurements taken in the presence of O_2. As with the H_2O_2 experiments shown above (Figure S12), experiments were performed using holo and then apo protein to allow clear differentiation between the electrocatalytic activity of the LPMO active site vs the underlying electrode surface.

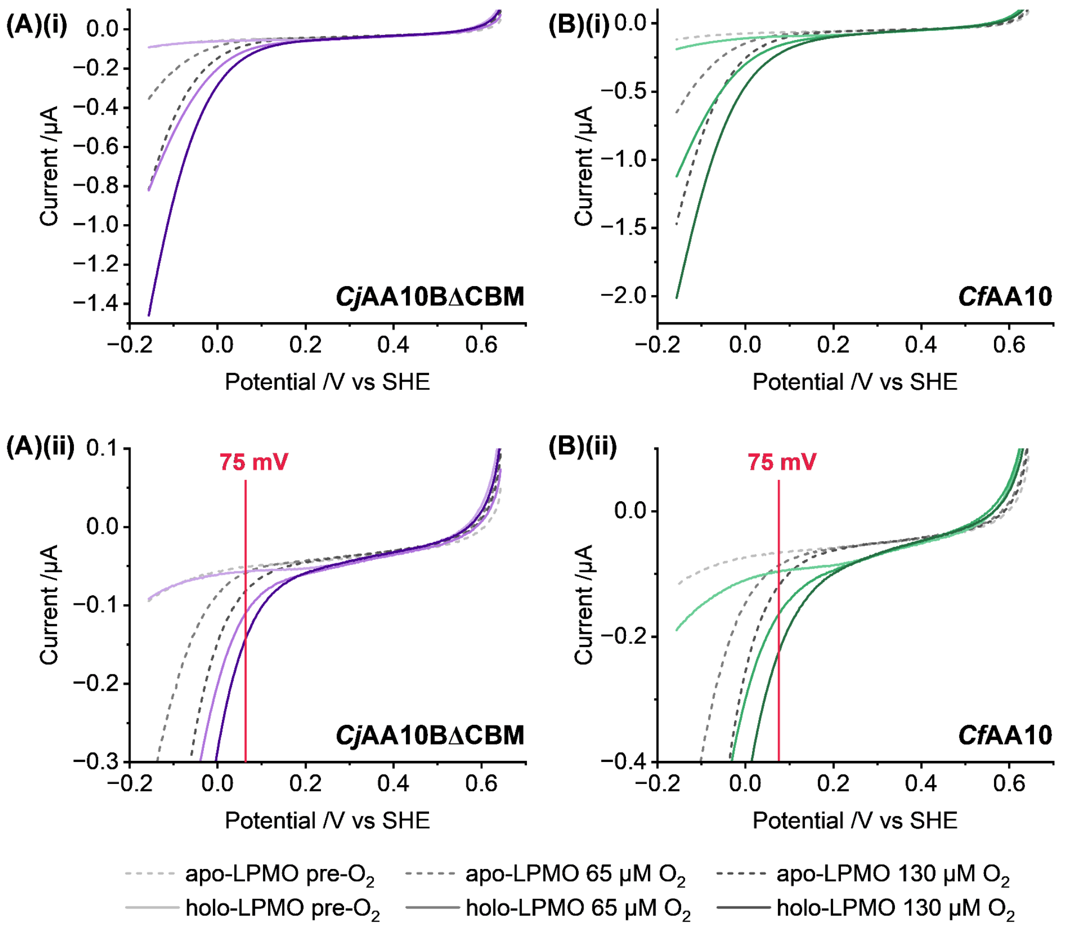

Figure S18 Voltammetric data taken of (A) *Cj*AA10BΔCBM$_{\text{C-His}}$ and (B) *Cf*AA10, pre (lighter lines) and post (darker lines) addition of O_2. Holo-LPMO are shown with solid lines whilst apo-LPMO controls are represented with dashed lines. Measurements were collected across a potential range of 640 to -160 mV vs SHE at 10 mV s^{-1} in pH 5.0 buffer solution at 35 °C with the electrode rotator set to 2000 rpm. The vertical red lines at 75 mV denote the constant voltage employed in the chronoamperometry experiments reported in the main paper. (A)(ii) and (B)(ii) are zoomed in versions of (A)(i) and (B)(i), allowing for clearer visualisation of the differences between Apo- and Holo-LPMO.

Accounting for H_2O_2 production at the bare electrode

The total charge passed over the course of the bare-electrode chronoamperometry experiment was calculated by integrating the area under the current-time trace (see Figure S19A); to give 0.0985 C. Dividing this number by F x 2 provides a maximum number of moles of H_2O_2 present. Finally, dividing through by the volume of the cell (25 mL) leaves us with a final concentration of 20 μM H_2O_2 present in the system at the end of the experiment. As calculated earlier, the K_M of the H_2O_2 reduction by an LPMO falls in the mM range and from this we can safely conclude that the amount of H_2O_2 generated *in situ* by the bare electrode is too small to elicit a detectable electrocatalytic response from the LPMO. To further control for the potential build-up of H_2O_2 at the electrode surface leading to catalytic turnover, the experiment in Figure S19B was performed. Stopping the rotation would correspond with the increase of the local concentration of H_2O_2 at the electrode and if this H_2O_2 was causing the bulk current response it could be expected to increase when the electrode is stationary. Instead, current drops when the electrode stops rotating, suggesting that it is in fact the O_2 flux that is responsible for the current response.

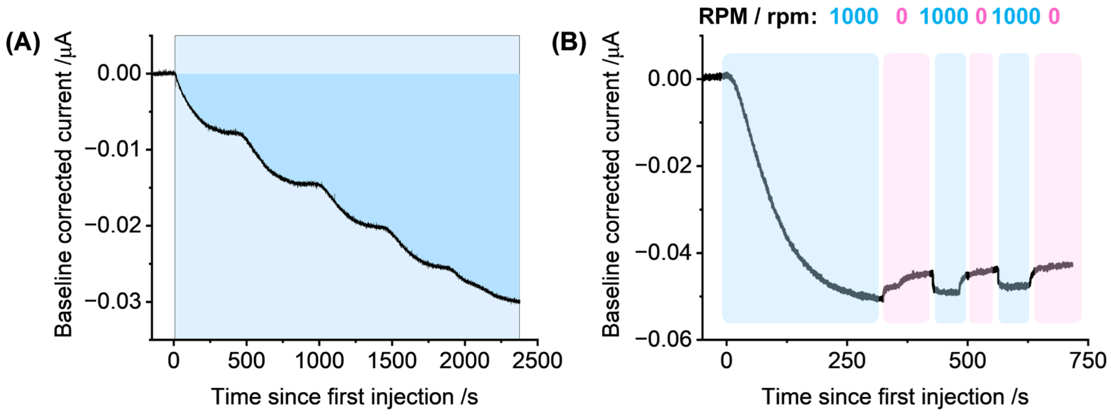

Figure S19(A) Integration of the area under the current-time trace recorded for the O_2-reduction ability of the bare electrode - “blank” trace in Figure 8(A)(i)/(B)(i). **(B)** Chronoamperometric response of a film of *Cf*AA10 in the presence of 130 μM O_2 with either electrode rotation at 2000 rpm (regions shaded in blue) or no electrode rotation (0 rpm) (regions shaded in pink).

Electrocatalytic Assay Analysis

Current extraction

As shown in the H_2O_2 chronoamperometry data in the main paper, after each H_2O_2 injection there is a spike in reductive current which quickly levels off, resulting in a flat “step” as illustrated in Figure S20. The current of each step is then averaged. The averaged current response from the blank electrode at each H_2O_2 concentration is subtracted from the equivalent protein experiment. The blank-corrected, enzyme-only current is then used for Michaelis-Menten analysis.

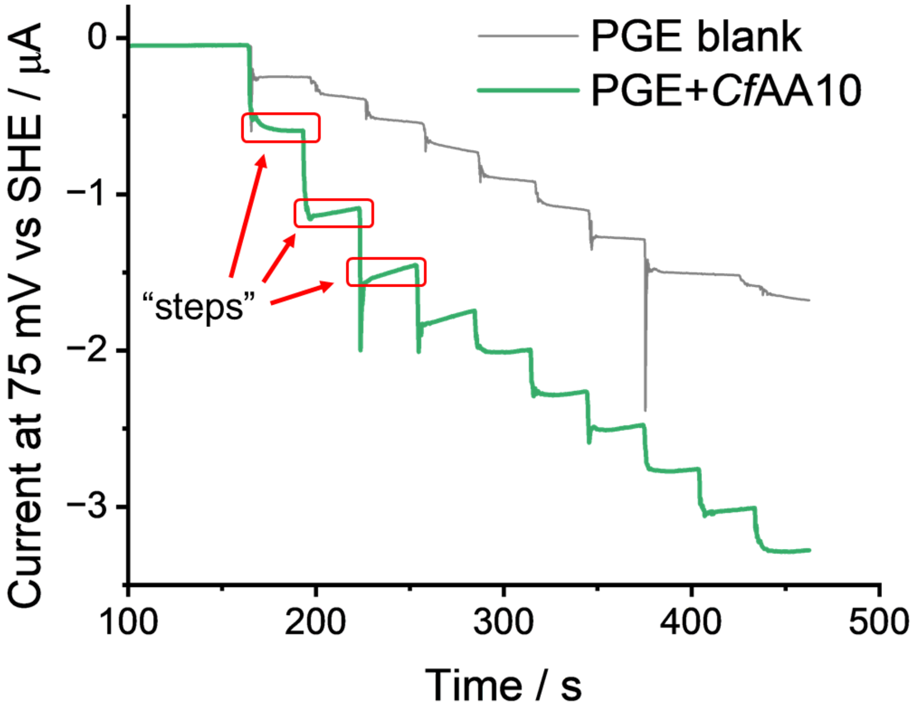

Figure S20 Illustration of the "steps" observed over the course of a chronoamperometric H_2O_2 assay carried out on *Cf*AA10, adapted from Figure 7 of the main paper.

Michaelis Menten and Lineweaver-Burk Analysis - H_2O_2

The blank-corrected current is plotted against the concentration of H_2O_2 resulting in a Michealis-Menten plot, as shown in Figure 7 of the main paper. Alternatively, the inverse of the blank-corrected, enzyme-only current can be plotted against the inverse of the H_2O_2 concentration yielding a Lineweaver-Burk plot, as shown in Figure S21. Equation S3 can be used to extract the kinetic parameters of i_{max} (maximum current response) and K_M (Michaelis constant) from these Lineweaver-Burk plots. A value of k_{cat} can be calculated as described in the main paper.

$$\frac{1}{i} = \frac{K_M}{i_{max}[S]} + \frac{1}{i_{max}}$$ **Equation S3**

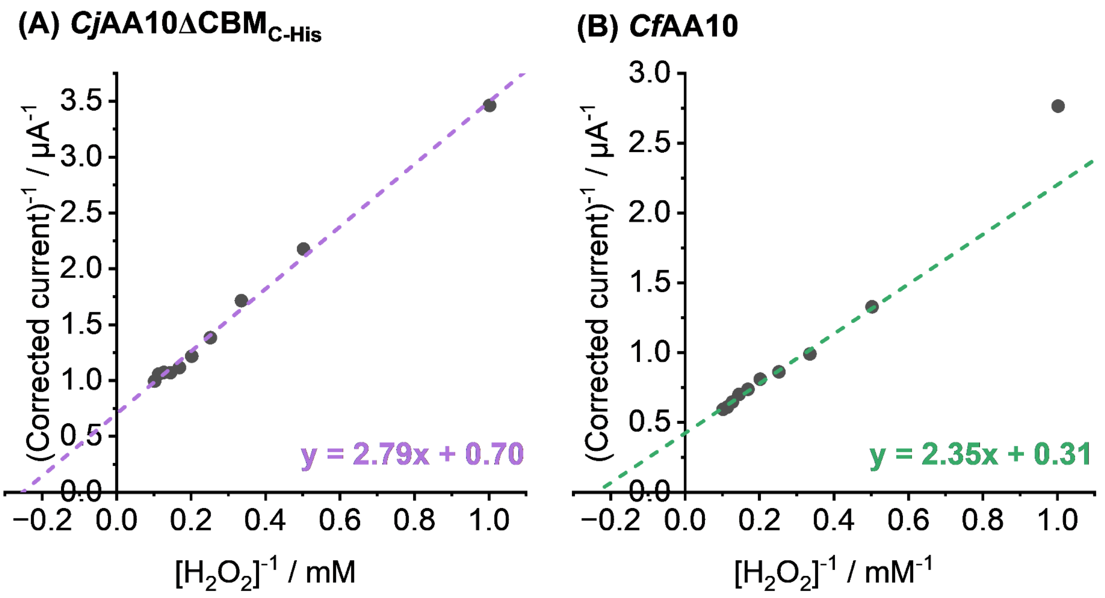

Figure S21 Lineweaver-Burk plots for (A) *Cj*AA10BΔCBM$_{C\text{-}His}$ (purple) and (B) *Cf*AA10 (green) produced by plotting the inverse of the corrected current response against the inverse of [H_2O_2], black points are experimental data and dotted line is best-fit.

The resulting parameters can be compared to those extracted from directly fitting to the Michaelis-Menten curves, shown in Figure 7 of the main paper. Both methods of extracting kinetics result in similar values, as shown in Table S2.

Table S2 Comparison of values extracted via Michaelis-Menten (M-M) versus Lineweaver-Burk (LW-B) analysis for H_2O_2 electrochemical assays

	i_{max} / μA		K_M / mM		k_{cat} / s^{-1}	
	**M-M**	**LW-B**	**M-M**	**LW-B**	**M-M**	**LW-B**
***Cj*AA10BΔCBM$_{C\text{-}His}$**	1.41	1.42	3.88	3.96	1.28	1.29
***Cf*AA10**	4.40	4.61	7.76	8.25	3.81	3.99

Michaelis Menten and Lineweaver-Burk Analysis - O_2

The analysis of the O_2 assay was performed identically to that of the H_2O_2 assay described above. Each "step" was averaged and the inverse of the current was plotted against the inverse of the substrate concentration as shown in Figure S22. Kinetic parameters were then extracted using Equation S3.

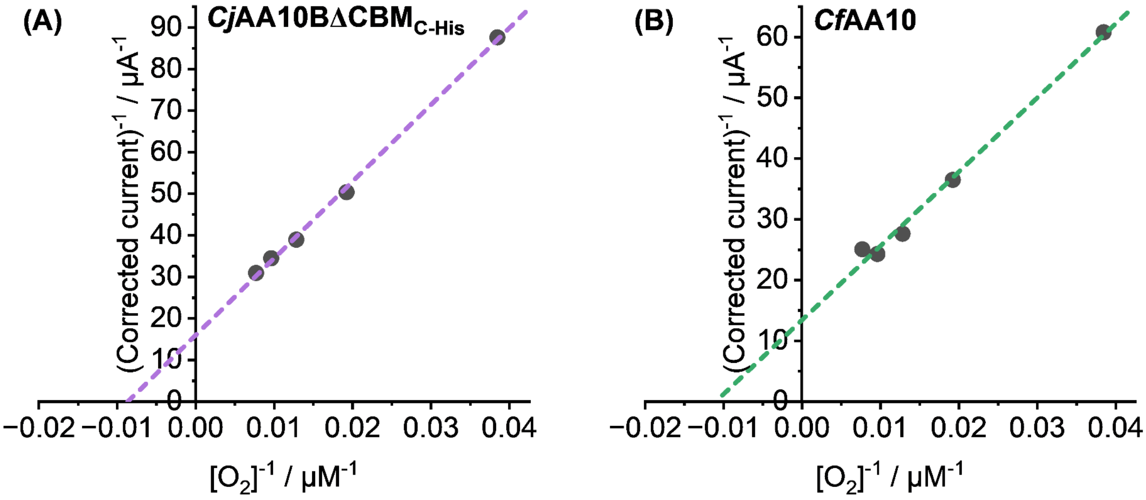

Figure S22 Lineweaver-Burk plots of *Cf*AA10 and *Cj*AA10BΔCBM$_{C\text{-His}}$, produced by plotting the inverse of the current response against the inverse of [O_2]. A linear fit was used to extract the kinetic parameters as detailed above.

A comparison of the kinetic values extracted from the Michaelis-Menten and equivalent Lineweaver-Burk plots is shown below in Table S3.

Table S3 Comparison of values extracted via Michaelis-Menten (M-M) versus Lineweaver-Burk (LW-B) analysis for O_2 electrochemical assays

	i_{max} / μA		K_M / μM		k_{cat} / s^{-1}	
	**M-M**	**LW-B**	**M-M**	**LW-B**	**M-M**	**LW-B**
***Cj*AA10BΔCBM$_{C\text{-His}}$**	0.056	0.062	95	115	0.026	0.029
***Cf*AA10**	0.065	0.074	69	90	0.073	0.083

Error Propagation

Error propagation for the specificity constant was performed using Equation S4.

Equation S4

$$\Delta c = \frac{a}{b} \times \sqrt{\left(\frac{\Delta a}{a}\right)^2 + \left(\frac{\Delta b}{b}\right)^2}$$

Literature Values for the 2,6-Dimethoxyphenol Assay

Table S4 Table of literature values for the 2,6-dimethoxyphenol assay. An enzyme unit U, is defined as 2 μmol of 2,6-DMP consumed per minute. The values obtained in this study are indicated in the shaded dark blue boxes.

LPMO	Family	pH	k_{cat} (s^{-1})	U g^{-1}
BaLPMO10A[1]	AA10	6.0	0.042	N/A
NcLPMO9C[2]	AA9	6.0	0.57	N/A
AfAA9B[3]	AA9	6.0	0.021	N/A
TthLPMO9G[4]	AA9	6.0	N/A	11.5
NcLPMO9C[5]	AA9	7.5	N/A	32.5
StAA9[6]	AA9	6.0	0.0031	N/A
McAA9[6]	AA9	6.0	0.0020	N/A
TrAA14A[7]	AA14	7.0	2.95	N/A
***Cf*AA10**	AA10	7.5	0.11	1.16
***Cj*AA10BΔCBM$_{C\text{-}His}$**	AA10	7.5	0.015	0.11

Included are values for 3 LPMO families; AA10s[9], AA9s[3,10–13] and AA14s[14]

Solution Voltammetry of 2,6-dimethoxyphenol at Different pH

Solution voltammetry experiments were performed to investigate the impact of pH on the reduction potential of 2,6-DMP. Buffer solutions were prepared at a range of pHs from pH 4 to pH 8 containing 20 mM sodium acetate, 20 mM sodium phosphate and 500 mM sodium sulfate. A stock solution of 10 mM 2,6-DMP was prepared for solution voltammetry. For each CV experiment, 22.5 mL buffer solution was introduced to the electrochemical cell before the addition of 2.5 mL of 2,6-DMP stock solution, diluting the concentration of 2,6-DMP to 1 mM. Cyclic voltammetry was performed across a potential range in order to isolate the signal relating to 2,6-DMP oxidation (between 290-440 mV vs SHE to 840 mV vs SHE). As shown in Figure S23, the peak potential of the 2,6-DMP oxidation shifts to more negative potentials with an increase in pH. This observation corresponds with what is observed in a lack of activity of the dye assays (described above) at lower pHs. As the LPMO is not impacted by changes in pH, the ability for the 2,6-DMP to reduce

the copper site is dependent on the system being at the correct pH to overlap with the 2,6-DMP oxidation peak.

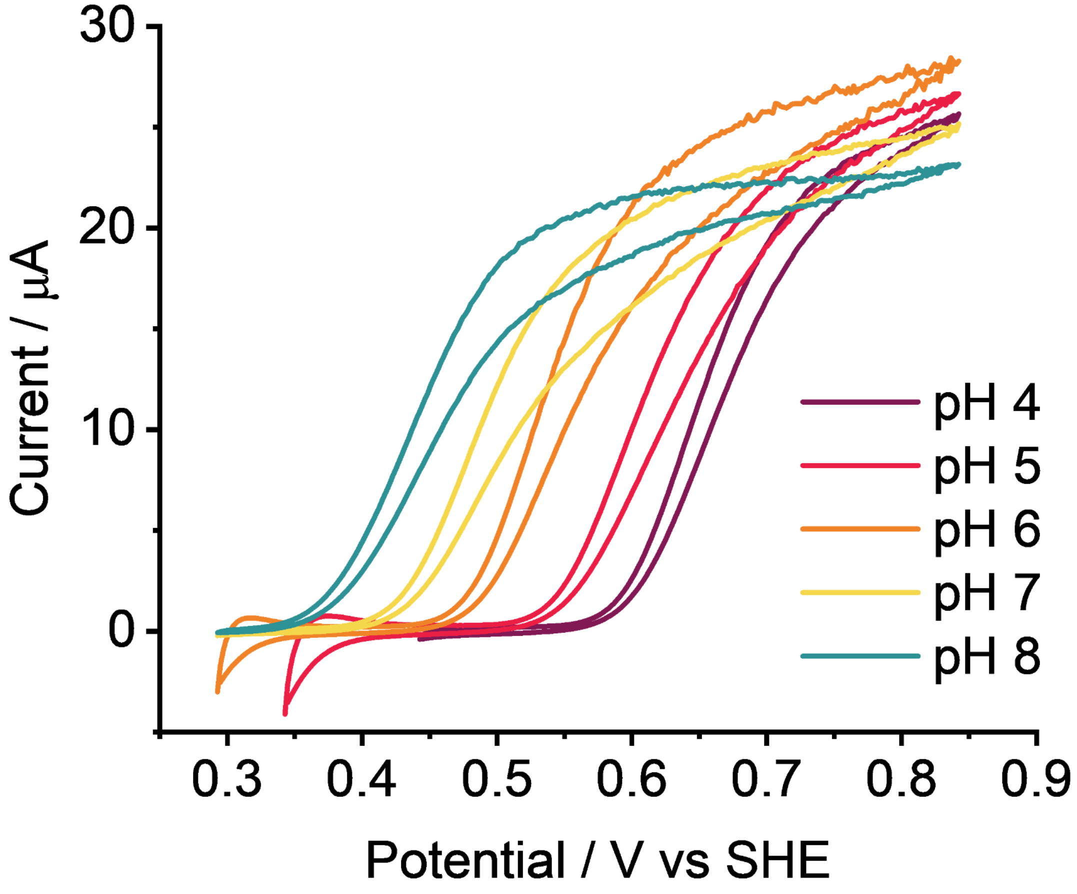

Figure S23 Solution CVs of 1 mM, 2,6-DMP at pH 4.01, pH 5.01, pH 6.00, pH 6.99 and pH 8.02. CVs were recorded over a potential range from 0.29/0.39/0.44 to 0.84 V vs SHE at 10 mV s^{-1} and 2000 rpm at 35 °C.

Impact of pH on Non-Catalytic Copper Redox Activity

Direct current voltammetry experiments shown in Figure S24 confirm the pH-induced change in peak-size is a reversible process. A film of *Cf*AA10 was drop-cast onto the surface of the working electrode and added to the electrochemical cell containing 25 mL of pH 4.0 buffer solution. A DCV experiment was performed at 5 mV s^{-1} before a buffer exchange to pH 4.5 followed by a further DCV experiment. The buffer was then exchanged up to pH 5.0 and scanned before dropping the experiment pH back to pH 4.0 and performing a final DCV scan. The LPMO signal can be seen to decrease from pH 4.0 to pH 5.0 before increasing upon reintroduction to pH 4.0.

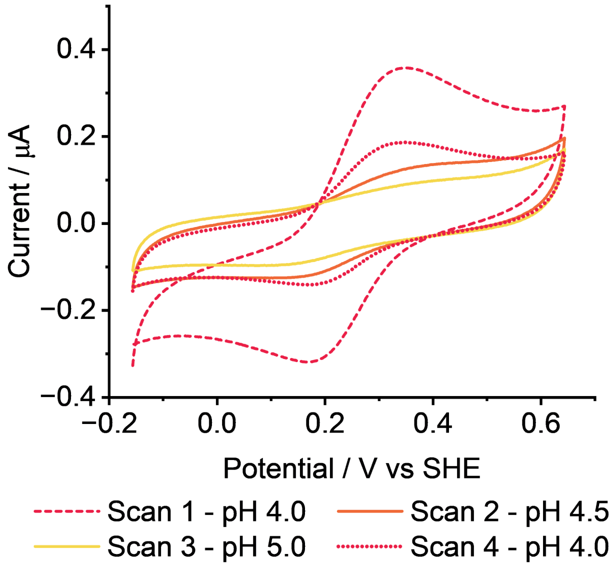

Figure S24 Impact of cycling pH on *Cf*AA10. An overlay of DCV experiments shows the decrease of signal intensity from pH 4.0 (dashed red line) to pH 4.5 (solid orange line) to pH 5.0 (solid yellow line) followed by an increase in intensity as the final experimental conditions are reverted to pH 4.0 (dotted red line). All buffer solutions consisted of 20 mM sodium acetate, 20 mM sodium phosphate and 500 mM sodium sulfate.All experiments were carried out under a N_2 environment at 35 °C across a potential range of -0.16 to 0.64 V vs SHE at a scan rate of 5 mV s^{-1}.

A further pH cycling experiment was performed, this time using SWV. A sample of LPMO was adsorbed onto the surface of the working electrode. Once the film had dried, a DCV experiment was carried out in pH 5.0 buffer solution at 2000 rpm, allowing the signal to equilibrate. After the signal had stabilised, a reductive sweep SWV experiment was performed at 2 Hz using a 10 mV pulse amplitude. After performing a pH 5.0 SWV scan, the working electrode was removed from the cell and rinsed with MilliQ water. The buffer solution in the cell was exchanged to pH 6.0 and another SWV scan was performed. This process was repeated, cycling between pH 5.0 and pH 6.0. Figure S25(A)(i) and S25(B)(ii) show SWV responses from *Cj*AA10BΔCBM and *Cf*AA10 respectively. Figure S25(A)(i) and S25(B)(ii) show the same responses after baseline-subtraction to ensure that the peak intensities are easily comparable. For both *Cj*AA10BΔCBM and *Cf*AA10, a signal with a maximum peak intensity of ~0.02 µA is seen, this signal then diminishes when the buffer is exchanged to pH 6.0, before recovering to almost the same level when returned to pH 5.0. This process can be repeated multiple times with the same decrease followed by an increase in peak intensity when transitioning from pH 5.0 to pH 6.0 and back to pH 5.0. The signal not returning to its original intensity can be attributed to film loss. This experiment again proves that the change in signal size with pH is a reversible process.

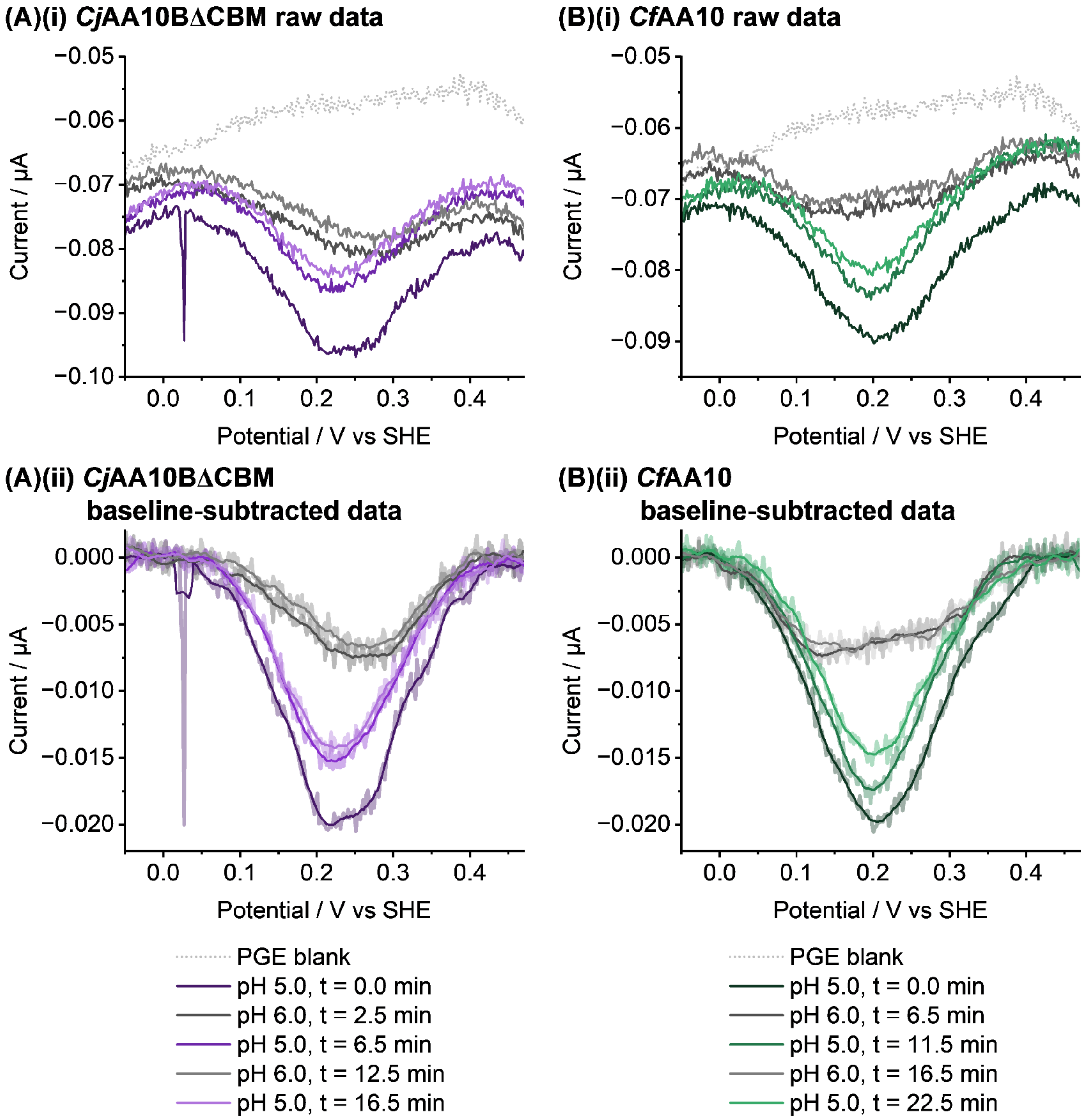

Figure S25: SWV experiment showing a reductive sweep of (A)(i) *Cj*AA10BΔCBM and (B)(ii) *Cf*AA10 in pH 5.0 vs pH 6.0 buffer solution. (A)(ii) and (B)(ii) show the same data after baseline subtraction. Scans are shown from -50 to 470 mV vs SHE, taken with a 2 Hz frequency, 10 mV pulse amplitude and 2 mV E_{step}. All experiments were carried out at 35°C with the working electrode rotating at a rate of 2000 rpm.

As shown in Figure 26, SWV experiments across a wider pH range were carried out on *Cf*AA10, with complementary constant-pH control experiments to quantify the decay in signal over the same time period.

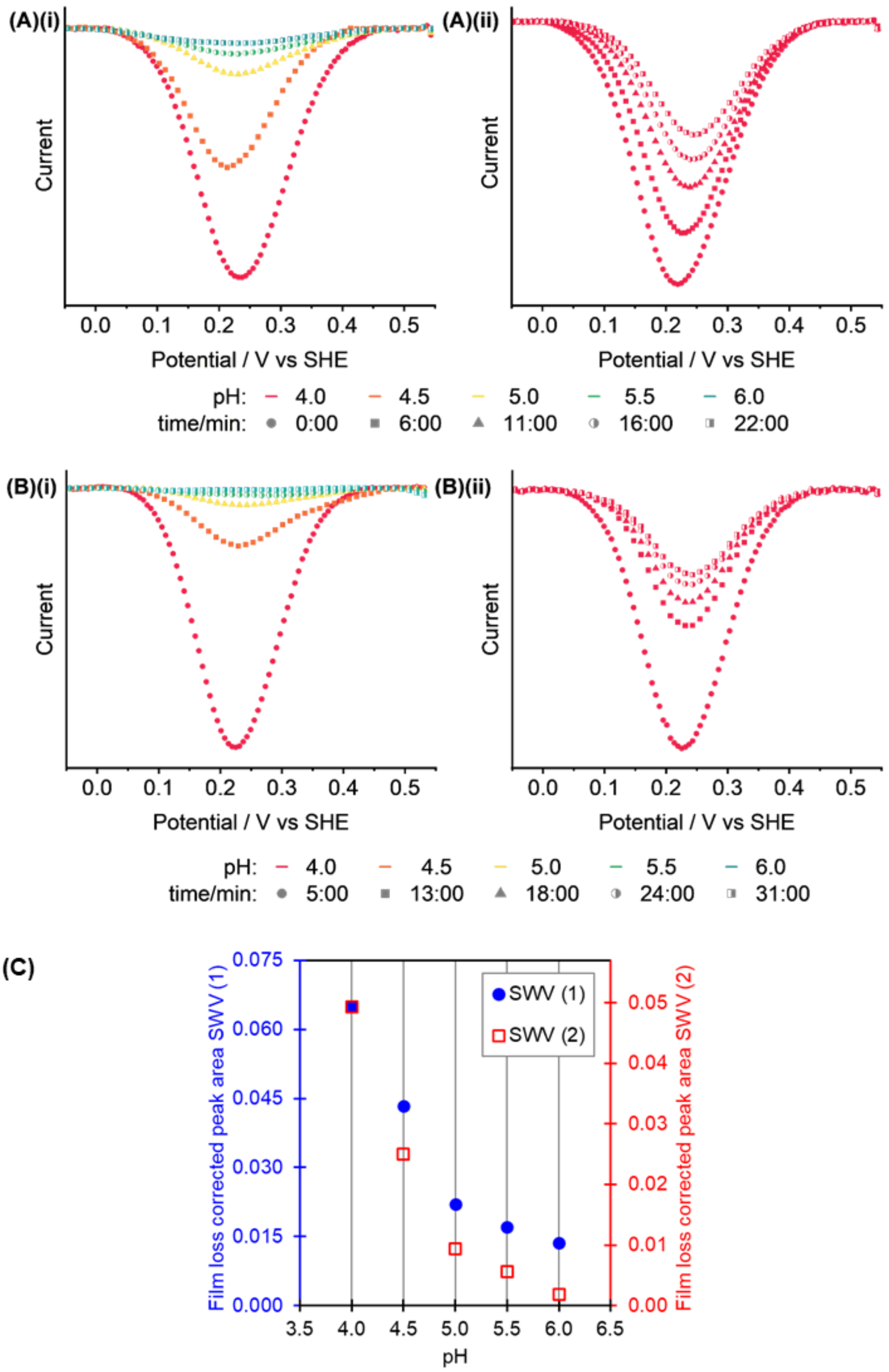

Figure S26 (A) Repeat 1 and (B)repeat 2 of non-catalytic SWV voltammetry experiments on *Cf*AA10 where the pH is steadily raised over time from pH 4.0 to pH 6.0, and control experiments where the pH is held constant at pH 4.0 and data is recorded at the same points in time. (C) Film-loss corrected square wave voltammetry (SWV) peak area vs pH for the two separate experiments (SWV (1) blue solid circles, left y-axis; SWV (2) red open boxes, right y-axis). All measurements were recorded across a potential range from 0.54 to -0.06 V vs SHE at 35 °C under an atmosphere of N_2.

The impact of pH on the kinetics of the $Cu^{2+/1+}$ electron transfer process was analysed using simulations of SWV scans taken of *Cf*AA10 at pH 4.0, pH 4.5 and pH 5.0 (data taken from experiment described in Figure S26). Figure S27 (A-C) shows these simulations overlaid on top of the raw experimental data with (D) MCMC analysis that visually reports the parameter combinations that can be used to give good fits between the mathematical model of the electron transfer and the experimental data.

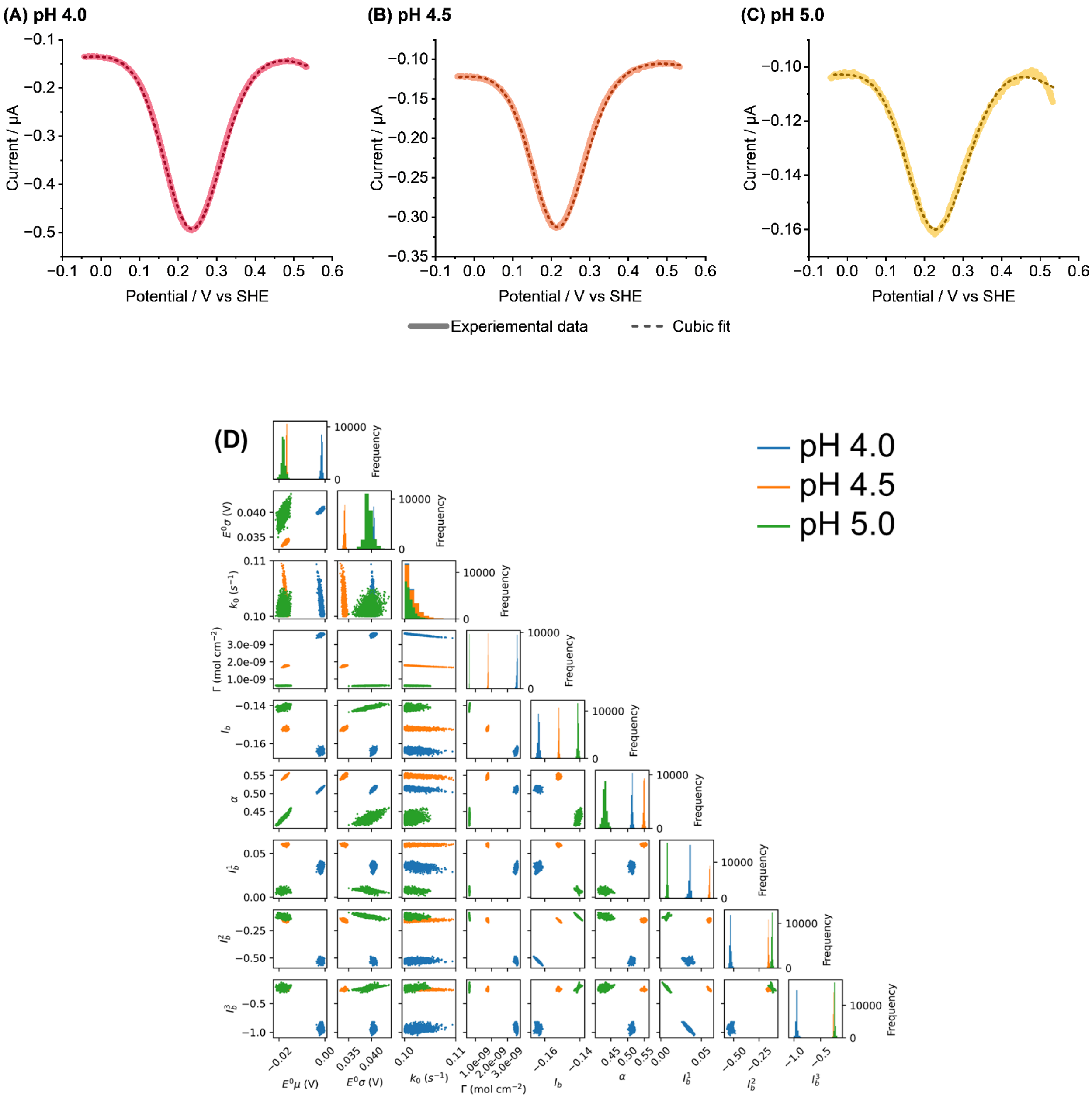

Figure S27: Overlays of experimental data with best-fit using a cubic model to account for non-Faradaic current of the reductive sweep of SWV measurements taken for *Cf*AA10 across a potential range of -60 → 540 mV vs SHE at a range of pHs; (A) pH 4.0, (B) pH 4.5 and (C) pH 5.0. In all cases, the Faradaic $Cu^{2+/1+}$ current is modelled using the Butler-Volmer equation. All experiments were carried out using a 10 mV pulse amplitude and a 2 Hz frequency under an atmosphere of N_2. Markov chain Monte Carlo (MCMC) analysis of this data is shown in Figure S22D.

LPMO Surface Charge

Analysis of the surface charge of the LPMOs was performed to determine if this provided an explanation for the observed change in DCV

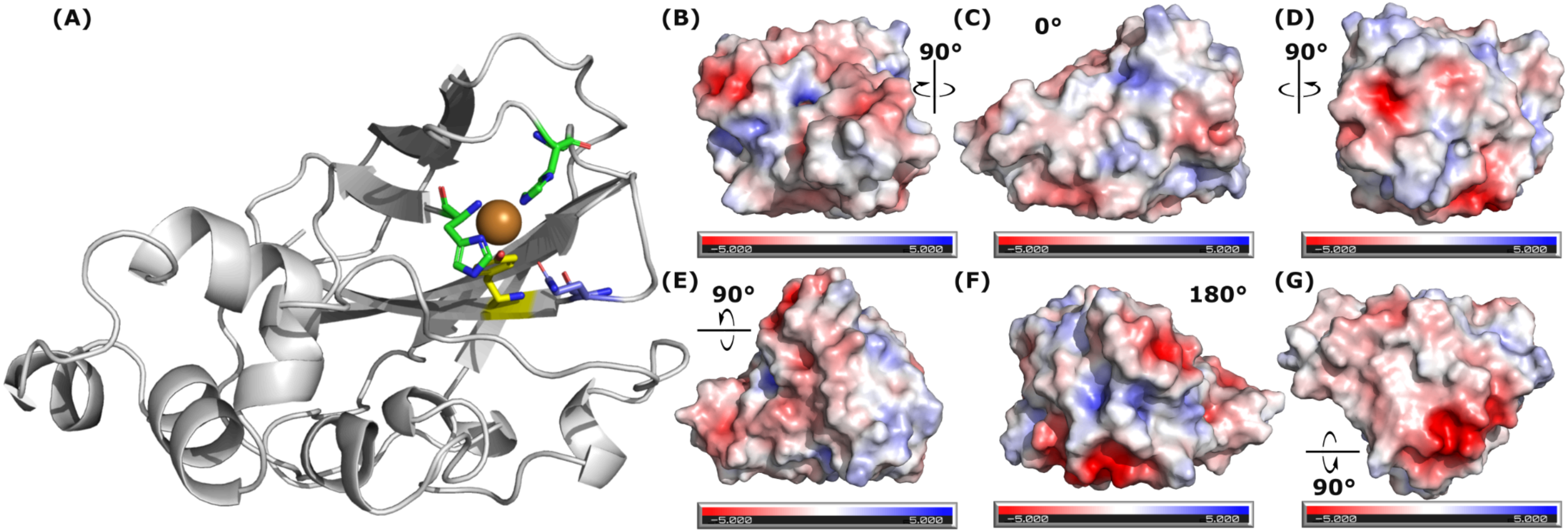

Figure S28 Surface charge of the predicted structure of *Cf*AA10. Negative charges are shown in red, positive charges are shown in blue. (A) Front cartoon view of the predicted structure of *Cf*AA10 with active site residues displayed in sticks. (B) Surface charge of *Cf*AA10 rotated 90° Y anticlockwise from front view. (C) Surface charge of front view (D) Surface charge of *Cf*AA10 rotated 90° Y clockwise from front view. (E) Surface charge of *Cf*AA10 rotated 90° X clockwise from front view. (F) Surface charge of *Cf*AA10 rotated 180° Y from front view. (G) Surface charge of *Cf*AA10 rotated 90° x anticlockwise from front view.

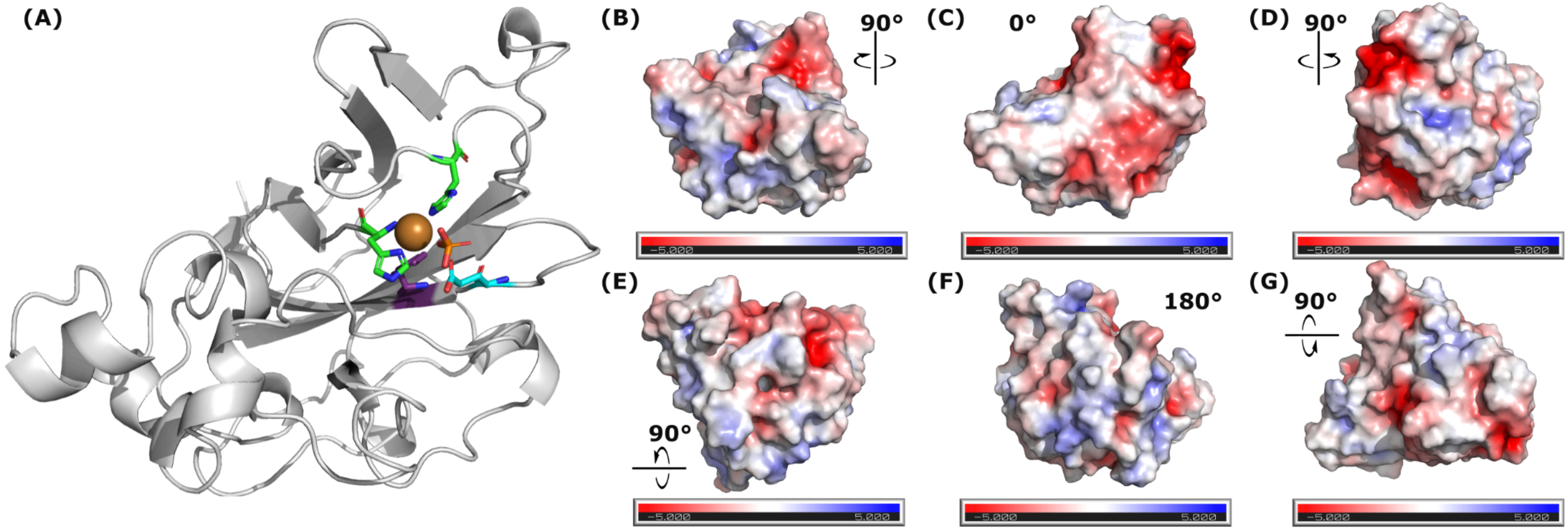

Figure S29 Surface charge of the crystal structure of *Cj*AA10BΔCBM. Negative charges are shown in red, positive charges are shown in blue. (A) Front cartoon view of the predicted structure of *Cj*AA10BΔCBM with active site residues displayed in sticks. (B) Surface charge of *Cj*AA10BΔCBM rotated 90° Y anticlockwise from front view. (C) Surface charge of front view (D) Surface charge of *Cj*AA10BΔCBM rotated 90° Y clockwise from front view. (E) Surface charge of *Cj*AA10BΔCBM rotated 90° X clockwise from front view. (F) Surface charge of *Cj*AA10BΔCBM rotated 180° Y from front view. (G) Surface charge of *Cj*AA10BΔCBM rotated 90° x anticlockwise from front view.

EPR Simulation Details

The EPR data was simulated heuristically using EasySpin.

	***Cj*AA10BΔCBM**	***Cf*AA10**
**pH 8.0**	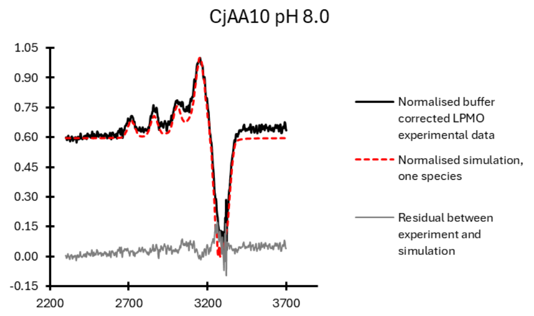	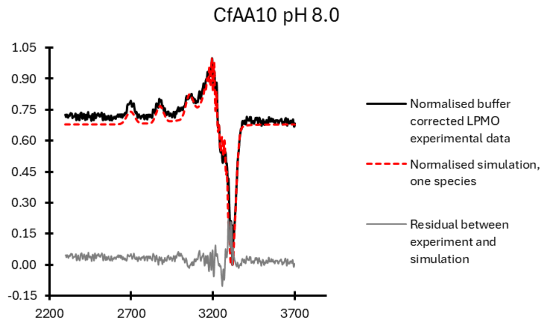
**pH 7.0**	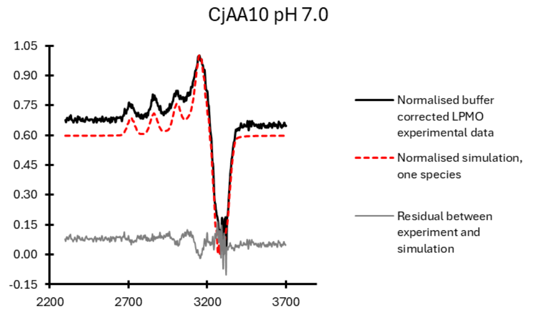	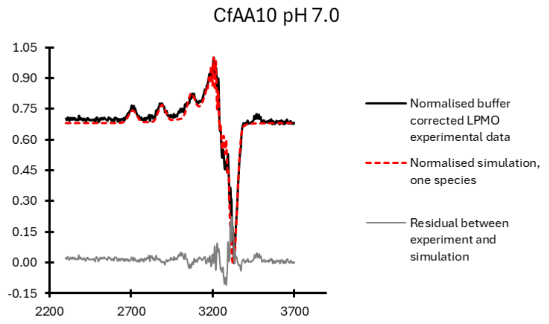
**pH 6.0**	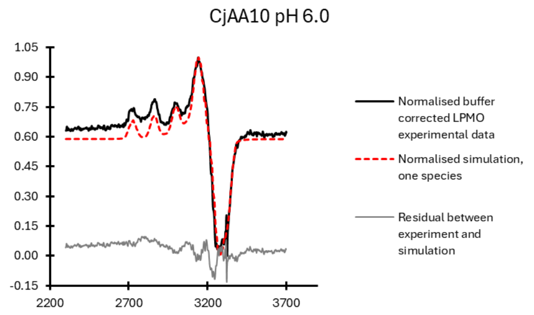	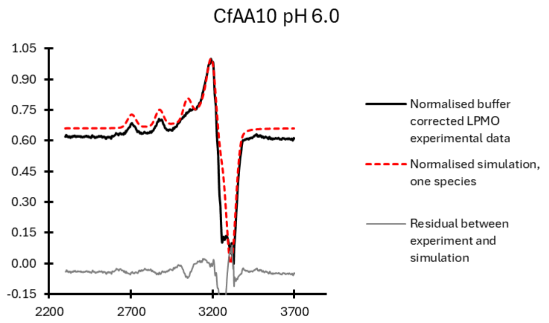
**pH 5.0**	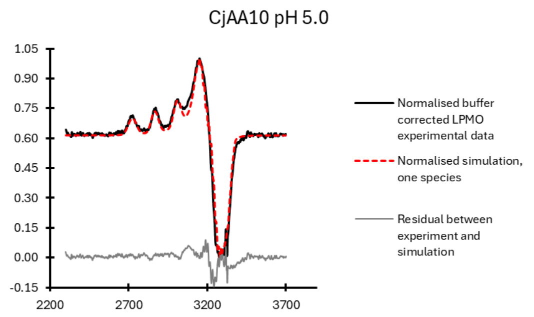	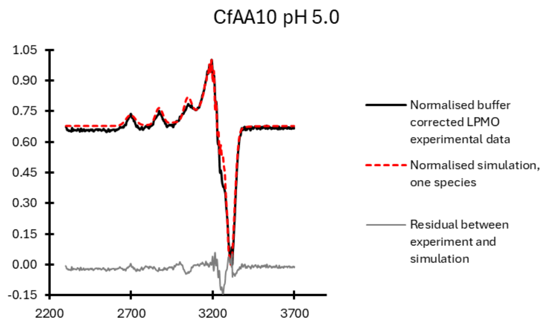

<table>
<tr><td rowspan="3">pH 4.5</td><td>Simulations of species 1 only</td><td>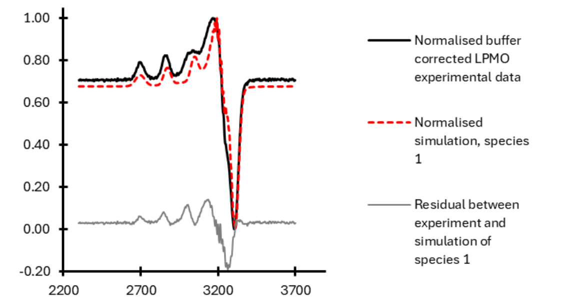
</td></tr>
<tr><td>Simulations of species 2 only</td><td>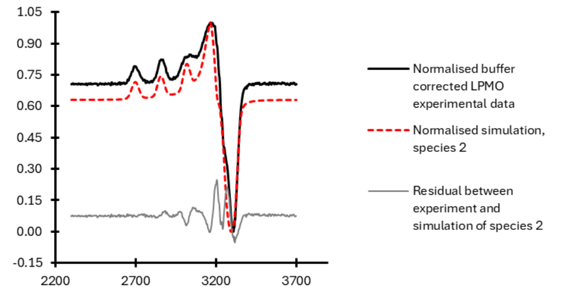
</td></tr>
<tr><td>Simulation of 44% species 1 + 56% species 2</td><td>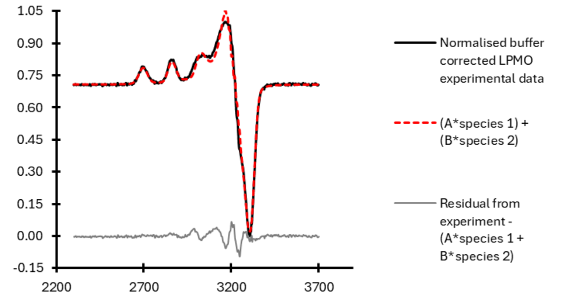
</td></tr>
</table>

Figure S30 Plots of normalised EPR experimental data (solid black lines), EPR simulations (red dashed lines) and the residual obtained from subtracting the normalised simulation from the experimental data (thin solid grey lines). All x-axis are the magnetic field in Gauss.

Table S5 EPR simulation parameters used to generate the simulation plots shown in Figure S30 and Figure 10.

	***Cj*AA10BΔCBM**	***Cf*AA10**	
	**Species 1**	**Species 1**	**Species 2**
**pH 8.0**	**g1:** 2.030; **g2:** 2.082; **g3:** 2.265 **Cu-A1:** 75; **Cu-A2:** 60; **Cu-A3:** 450 **N1-A:** 45, 45, 45; **N2-A:** 45, 45, 45; **N3-A:** 35, 35, 35 **LW:** 0.8, 0.8	**g1:** 2.046; **g2:** 2.064; **g3:** 2.238 **Cu-A1:** 35; **Cu-A2:** 55; **Cu-A3:** 550 **N1-A:** 45, 45, 45; **N2-A:** 43, 43,43; **N3-A:** 35, 35, 35 **LW:** 0.6, 0.6	N/A
**pH 7.0**	**g1:** 2.030; **g2:** 2.082; **g3:** 2.265 **Cu-A1:** 75; **Cu-A2:** 60; **Cu-A3:** 450 **N1-A:** 45, 45, 45; **N2-A:** 45, 45, 45; **N3-A:** 35, 35, 35 **LW:** 0.8, 0.8	**g1:** 2.046; **g2:** 2.064; **g3:** 2.238 **Cu-A1:** 35; **Cu-A2:** 55; **Cu-A3:** 550 **N1-A:** 45, 45, 45; **N2-A:** 43, 43, 43; **N3-A:** 35, 35, 35 **LW:** 0.6, 0.6	N/A
**pH 6.0**	**g1:** 2.032; **g2:** 2.080; **g3:** 2.27 **Cu-A1:** 95; **Cu-A2:** 70; **Cu-A3:** 425 **N1-A:** 45, 45, 45; **N2-A:** 45, 45, 45; **N3-A:** 35, 35,35 **LW:** 0.8, 0.8	**g1:** 2.042; **g2:** 2.072; **g3:** 2.249 **Cu-A1:** 60; **Cu-A2:** 30; **Cu-A3:** 525 **N1-A:** 45, 45, 45; **N2-A:** 43, 43, 43; **N3-A:** 35, 35, 35 **LW:** 1.5, 1.5	N/A
**pH 5.0**	**g1:** 2.034; **g2:** 2.086; **g3:** 2.266 **Cu-A1:** 85; **Cu-A2:** 80; **Cu-A3:** 445 **N1-A:** 45, 45, 45; **N2-A:** 43, 43, 43; **N3-A:** 35, 35, 35 **LW:** 0.8, 0.8	**g1:** 2.048; **g2:** 2.065; **g3:** 2.245 **Cu-A1:** 50; **Cu-A2:** 50; **Cu-A3:** 540 **N1-A:** 45, 45, 45; **N2-A:** 43, 43, 43; **N3-A:** 35, 35, 35 **LW:** 0.6, 0.6	N/A
**pH 4.5**	N/A	**g1:** 2.044; **g2:** 2.075; **g3:** 2.245 **Cu-A1:** 80; **Cu-A2:** 80; **Cu-A3:** 540 **N1-A:** 45, 45, 45; **N2-A:** 43, 43, 43; **N3-A:** 35, 35, 35 **LW:** 1.5, 1.5	**g1:** 2.040; **g2:** 2.080; **g3:** 2.260 **Cu-A1:** 85; **Cu-A2:** 60; **Cu-A3:** 495 **N1-A:** 45, 45, 45; **N2-A:** 35, 35, 50 **LW:** 1.5, 1.5

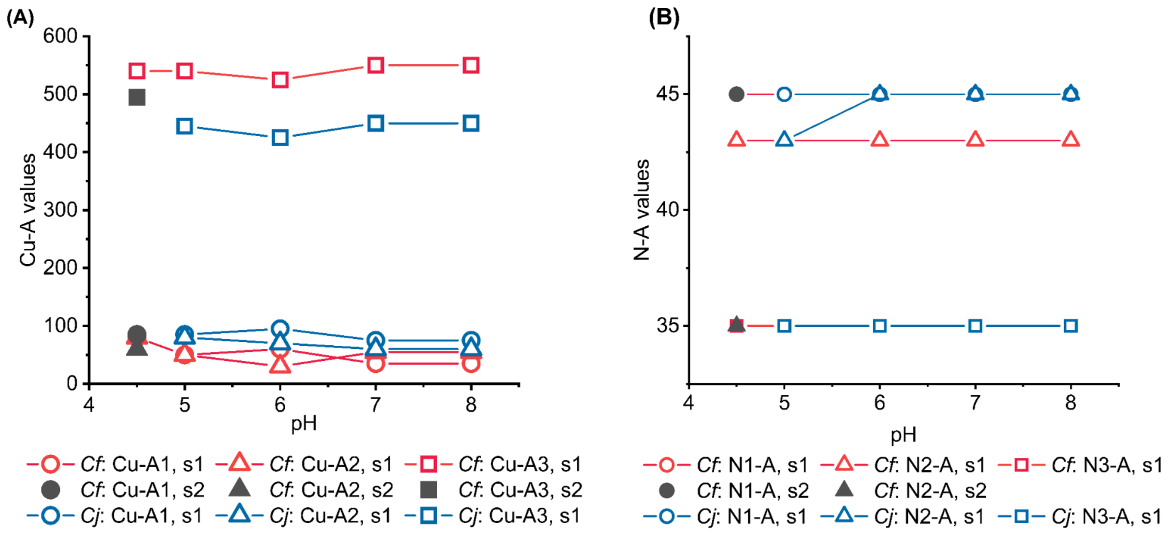

Figure S31 Plots of A values in Table S5 against the experimental pH. (A) Copper hyperfine splitting values, and (B) nitrogen hyperfine values.

Electrocatalytic Data at Different pHs

Figure S32 shows that when the current-time trace is corrected to subtract the current in zero substrate and adjust along the x-axis so that t_0 is the time of first substrate injection it is evident that there is no consistent correlation between the magnitude of current response and the pH of the experiment.

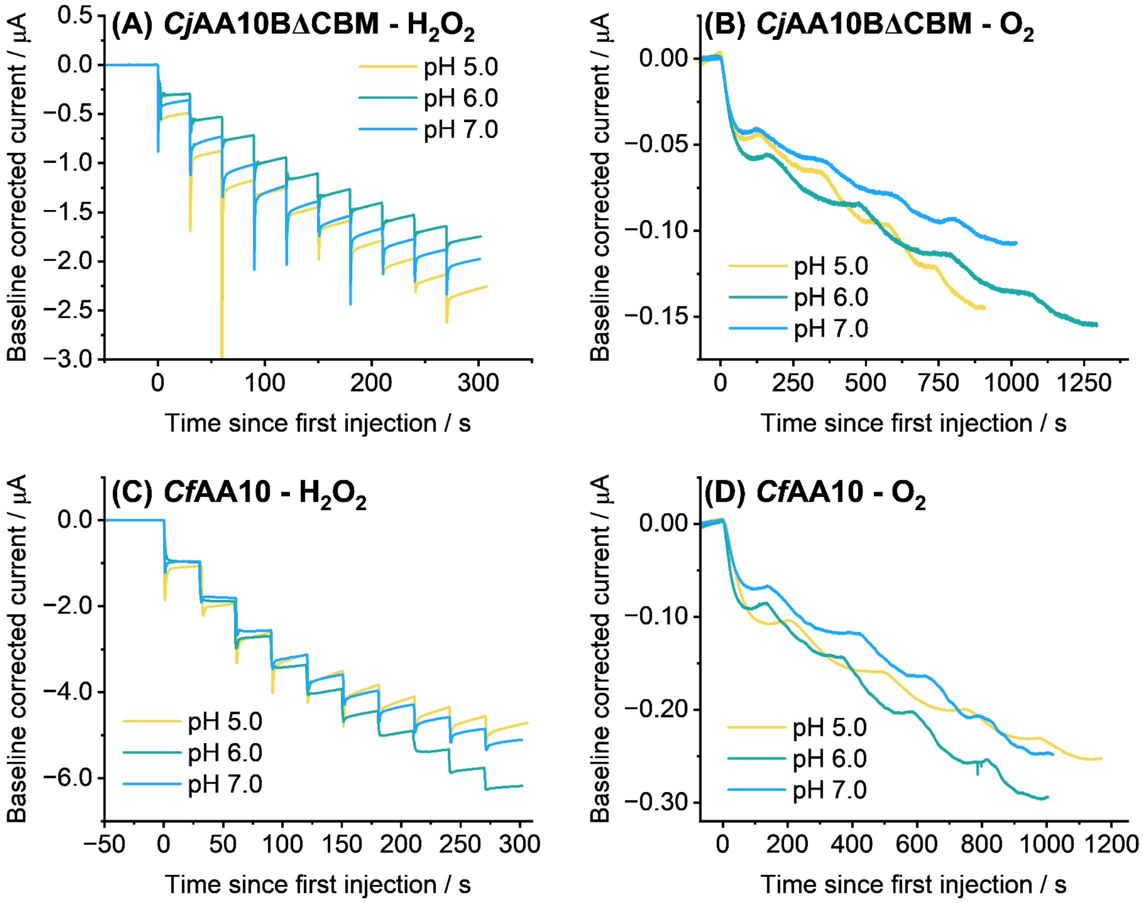

Figure S32 Experimental data collected for *Cj*AA10BΔCBM with (A) H_2O_2, (B) O_2 and *Cf*AA10 with (C) H_2O_2, (D) O_2 at pH 5.0, 6.0 and 7.0

Figure S33 shows a comparison of the extracted values of all i_{max} for both enzymes for both the H_2O_2 and O_2 assays. This indicates that there is no strong correlation between pH and electroactivity, even without accounting for the size of the LPMO film on the electrode.

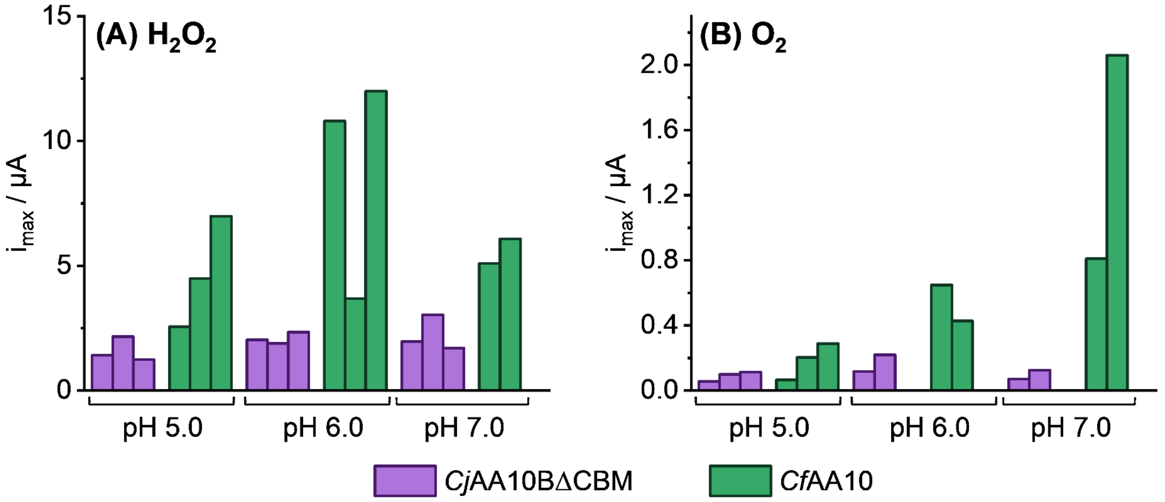

Figure S33: Bar charts showing variation in the extracted value of i_{max} from Michaelis-Menten fitting for (A) H_2O_2 and (B) O_2 assays. *Cj*AA10BΔCBM shown in purple and *Cf*AA10 shown in green.

Table S6 Electrocatalytic assay data for both H_2O_2 and O_2 assays for *Cj*AA10BΔCBM

H_2O_2, pH 5.0	i_{max} / μA	$\Gamma A/mol$	k_{cat} / s^{-1}	O_2, pH 5.0	i_{max} / μA	$\Gamma A/mol$	k_{cat} / s^{-1}
Repeat 1	1.41	6.81 x 10^{-12}	1.07	Repeat 1	0.056	1.11 x 10^{-11}	0.026
Repeat 2	2.16	9.80 x 10^{-12}	1.14	Repeat 2	0.099	1.43 x 10^{-11}	0.036
Repeat 3	1.24	1.67 x 10^{-11}	0.39	Repeat 3	0.113	1.39 x 10^{-11}	0.042
		Average (k_{cat}):	0.87			Average (k_{cat}):	0.035
		Standard error:	0.19			Standard error:	0.004
H_2O_2, pH 6.0	i_{max} / μA	$\Gamma A/mol$	k_{cat} / s^{-1}	O_2, pH 6.0	i_{max} / μA	$\Gamma A/mol$	k_{cat} / s^{-1}
Repeat 1	2.03	1.93 x 10^{-11}	0.54	Repeat 1	0.118	1.56 x 10^{-11}	0.039
Repeat 2	1.89	1.95 x 10^{-11}	0.50	Repeat 2	0.219	1.30 x 10^{-11}	0.088
Repeat 3	2.34	1.78 x 10^{-11}	0.68	-	-	-	-
		Average (k_{cat}):	0.57			Average (k_{cat}):	0.063
		Standard error:	0.04			Standard error:	0.014
H_2O_2, pH 7.0	i_{max} / μA	$\Gamma A/mol$	k_{cat} / s^{-1}	O_2, pH 7.0	i_{max} / μA	$\Gamma A/mol$	k_{cat} / s^{-1}
Repeat 1	1.96	1.39 x 10^{-11}	0.73	Repeat 1	0.070	1.50 x 10^{-11}	0.024
Repeat 2	3.03	1.54 x 10^{-11}	1.02	Repeat 2	0.125	1.69 x 10^{-11}	0.038
Repeat 3	1.70	1.32 x 10^{-11}	0.67	-			
		Average (k_{cat}):	0.81			Average (k_{cat}):	0.031
		Standard error:	0.09			Standard error:	0.004

Table S7 Electrocatalytic assay data for both H_2O_2 and O_2 assays for *Cf*AA10

**H_2O_2, pH 5.0**	i_{max} / μA	$\Gamma A/mol$	k_{cat} / s^{-1}	**O_2, pH 5.0**	i_{max} / μA	$\Gamma A/mol$	k_{cat} / s^{-1}
Repeat 1	2.56	5.98 x 10^{-12}	2.22	Repeat 1	0.065	4.62 x 10^{-12}	0.073
Repeat 2	4.49	3.30 x 10^{-11}	0.71	Repeat 2	0.203	3.68 x 10^{-11}	0.029
Repeat 3	6.99	3.14 x 10^{-11}	1.16	Repeat 3	0.288	3.49 x 10^{-11}	0.043
		Average (k_{cat}):	1.36			Average (k_{cat}):	0.048
		Standard error:	0.37			Standard error:	0.011
**H_2O_2, pH 6.0**	i_{max} / μA	$\Gamma A/mol$	k_{cat} / s^{-1}	**O_2, pH 6.0**	i_{max} / μA	$\Gamma A/mol$	k_{cat} / s^{-1}
Repeat 1	10.8	1.62 x 10^{-11}	3.47	Repeat 1	0.648	1.33 x 10^{-11}	0.250
Repeat 2	3.68	9.56 x 10^{-12}	2.00	Repeat 2	0.427	5.10 x 10^{-11}	0.043
Repeat 3	12.0	4.67 x 10^{-11}	1.33	-	-	-	-
		Average (k_{cat}):	2.27			Average (k_{cat}):	0.148
		Standard error:	0.51			Standard error:	0.060
**H_2O_2, pH 7.0**	i_{max} / μA	$\Gamma A/mol$	k_{cat} / s^{-1}	**O_2, pH 7.0**	i_{max} / μA	$\Gamma A/mol$	k_{cat} / s^{-1}
Repeat 1	5.09	4.37 x 10^{-11}	0.60	Repeat 1	0.81	4.46 x 10^{-11}	0.094
Repeat 2	6.08	5.10 x 10^{-11}	0.62	Repeat 2	2.06	5.88 x 10^{-11}	0.181
-	-	-	-	Repeat 3	-	-	-
		Average (k_{cat}):	0.61			Average (k_{cat}):	0.138
		Standard error:	0.004			Standard error:	0.025

Table S8: Electrocatalytic assay data for both *Cf*AA10 and *Cj*AA10BΔCBM including values for k_{cat}, K_M, and p-values from pH 5.0 - pH 7.0. All p-values are referenced to pH 5.0.

H_2O_2

*Cj*AA10ΔCBM		Repeat	k_{cat} / s^{-1}	Repeat	K_M / mM
	pH5	1	1.07	1	3.86
		2	1.14	2	6.04
		3	0.39	3	1.55
		Average (k_{cat})	0.87	Average (K_M)	3.81
		Standard error	0.20	Standard error	1.06
	pH6	1	0.54	1	4.19
		2	0.50	2	5.67
		3	0.68	3	4.62
		Average (k_{cat})	0.58	Average (K_M)	4.83
		Standard error	0.04	Standard error	0.36
	pH7	1	0.73	1	6.77
		2	1.02	2	6.09
		3	0.67	3	4.21
		Average (k_{cat})	0.81	Average (K_M)	5.69
		Standard error	0.09	Standard error	0.62

p-values (*Cj*AA10ΔCBM, H_2O_2): k_{cat} pH5–pH6 $p = 0.34$; pH5–pH7 $p = 0.83$. K_M pH5–pH6 $p = 0.52$; pH5–pH7 $p = 0.30$.

*Cf*AA10		Repeat	k_{cat} / s^{-1}	Repeat	K_M / mM
	pH5	1	1.36	1	4.79
		2	0.71	2	3.21
		3	1.16	3	9.00
		Average (k_{cat})	1.36	Average (K_M)	5.67
		Standard error	0.37	Standard error	1.41
	pH6	1	3.47	1	10.30
		2	1.33	2	11.77
		-	-	-	-
		Average (k_{cat})	2.40	Average (K_M)	11.04
		Standard error	0.51	Standard error	0.52
	pH7	1	0.60	1	6.70
		2	0.62	2	5.23
		-	-	-	-
		Average (k_{cat})	0.61	Average (K_M)	5.97
		Standard error	0.004	Standard error	0.52

p-values (*Cf*AA10, H_2O_2): k_{cat} pH5–pH6 $p = 0.31$; pH5–pH7 $p = 0.23$. K_M pH5–pH6 $p = 0.08$; pH5–pH7 $p = 0.88$.

O_2

*Cj*AA10ΔCBM		Repeat	k_{cat} / s^{-1}	Repeat	K_M / mM
	pH5	1	0.026	1	0.095
		2	0.036	2	0.185
		3	0.042	3	0.148
		Average (k_{cat})	0.035	Average (K_M)	0.143
		Standard error	0.004	Standard error	0.021
	pH6	1	0.039	1	0.101
		2	0.088	2	0.102
		-	-	-	-
		Average (k_{cat})	0.063	Average (K_M)	0.102
		Standard error	0.014	Standard error	0.0003
	pH7	1	0.024	1	0.063
		2	0.038	2	0.084
		-	-	-	-
		Average (k_{cat})	0.031	Average (K_M)	0.073
		Standard error	0.004	Standard error	0.007

p-values (*Cj*AA10ΔCBM, O_2): k_{cat} pH5–pH6 $p = 0.44$; pH5–pH7 $p = 0.71$. K_M pH5–pH6 $p = 0.26$; pH5–pH7 $p = 0.11$.

*Cf*AA10		Repeat	k_{cat} / s^{-1}	Repeat	K_M / mM
	pH5	1	0.073	1	0.069
		2	0.029	2	0.047
		3	0.043	3	0.093
		Average (k_{cat})	0.048	Average (K_M)	0.070
		Standard error	0.011	Standard error	0.011
	pH6	1	0.252	1	0.316
		2	0.043	2	0.309
		-	-	-	-
		Average (k_{cat})	0.148	Average (K_M)	0.316
		Standard error	0.060	Standard error	0.003
	pH7	1	0.094	1	0.614
		2	0.181	2	1.184
		-	-	-	-
		Average (k_{cat})	0.138	Average (K_M)	0.899
		Standard error	0.025	Standard error	0.201

p-values (*Cf*AA10, O_2): k_{cat} pH5–pH6 $p = 0.52$; pH5–pH7 $p = 0.27$. K_M pH5–pH6 $p = 0.001$; pH5–pH7 $p = 0.21$.

The information shown in Table S8 has been displayed below in Figure S34.

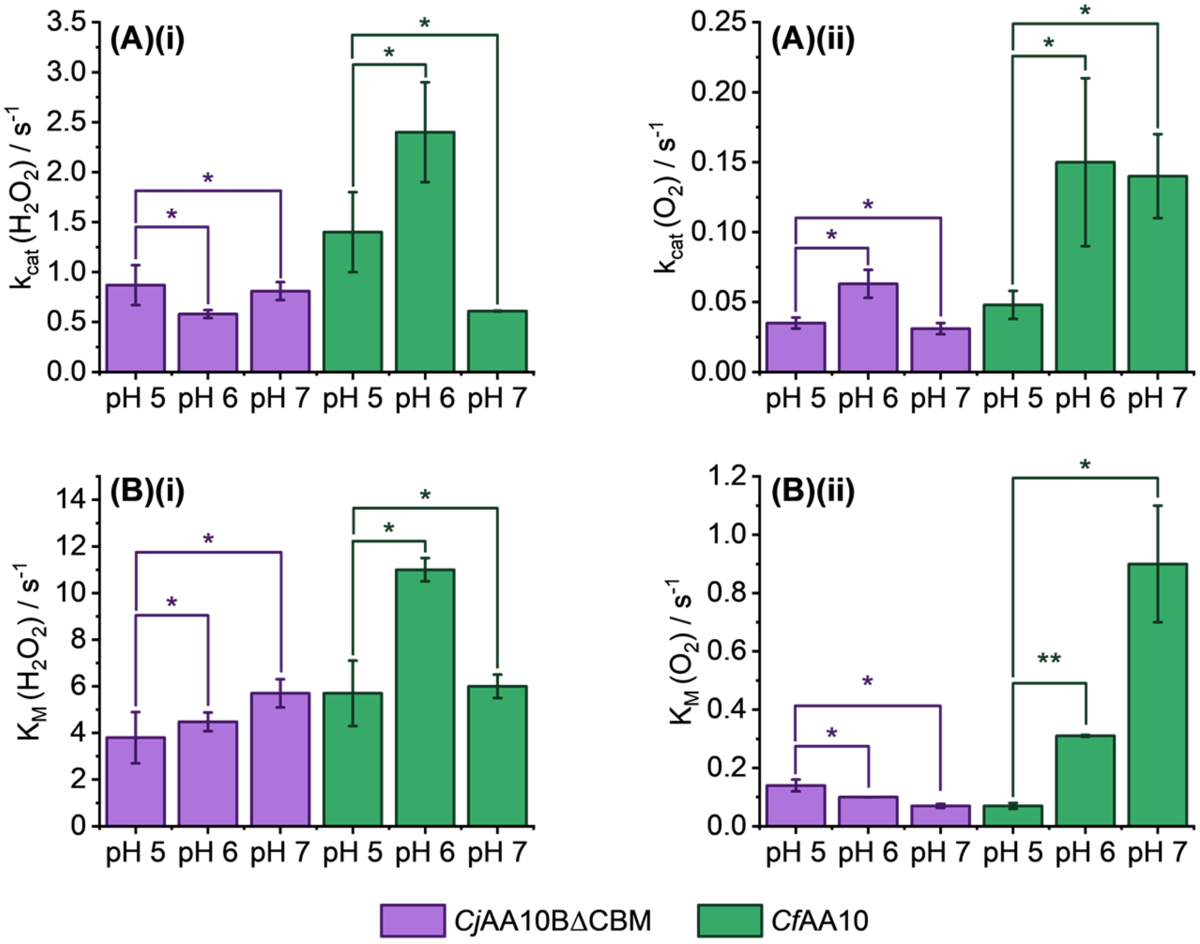

Figure S34: Bar charts showing average values for (A) k_{cat} and (B) K_M in the presence of (i) H_2O_2 and (ii) O_2 for *Cj*AA10BΔCBM and *Cf*AA10. P-values from a Student t-test are shown as * > 0.05 and ** < 0.05.

References

1 H. E. Klock and S. A. Lesley, in *High Throughput Protein Expression and Purification: Methods and Protocols*, ed. S. A. Doyle, Humana Press, Totowa, NJ, 2009, pp. 91–103.
2 J. Branch, B. S. Rajagopal, A. Paradisi, N. Yates, P. J. Lindley, J. Smith, K. Hollingsworth, W. B. Turnbull, B. Henrissat, A. Parkin, A. Berry and G. R. Hemsworth, *Biochem. J*, 2021, **478**, 2927–2944.
3 E. Breslmayr, M. Hanžek, A. Hanrahan, C. Leitner, R. Kittl, B. Šantek, C. Oostenbrink and R. Ludwig, *Biotechnol. Biofuels*, 2018, **11**, 79.
4 Resorufin, https://www.sigmaaldrich.com/GB/en/product/sigma/73144, (accessed 20 May 2024).
5 H. A. Heering, J. H. Weiner and F. A. Armstrong, *J. Am. Chem. Soc.*, 1997, **119**, 11628–11638.
6 C. F. Blanford and F. A. Armstrong, *J. Solid State Electrochem.*, 2006, **10**, 826–832.
7 V. Mirčeski and M. Lovrić, *Electroanalysis*, 1997, **9**, 1283–1287.
8 L. J. C. Jeuken and F. A. Armstrong, *J. Phys. Chem. B*, 2001, **105**, 5271–5282.
9 M. H. Berhe, X. Song and L. Yao, *Int. J. Mol. Sci.*, , DOI:10.3390/ijms24108963.
10 C. V. F. P. Laurent, P. Sun, S. Scheiblbrandner, F. Csarman, P. Cannazza, M. Frommhagen, W. J. H. van Berkel, C. Oostenbrink, M. A. Kabel and R. Ludwig, *Int. J. Mol. Sci.*, , DOI:10.3390/ijms20246219.
11 A. V. Bernardi, L. E. Gerolamo, P. F. de Gouvêa, D. K. Yonamine, L. M. S. Pereira, A. H. C. de Oliveira, S. A. Uyemura and T. M. Dinamarco, *Int. J. Mol. Sci.*, , DOI:10.3390/ijms22010276.
12 K. Chorozian, A. Karnaouri, N. Georgaki-Kondyli, A. Karantonis and E. Topakas, *Biotechnol Biofuels Bioprod*, 2024, **17**, 19.
13 D. Agrawal, N. Basotra, V. Balan, A. Tsang and B. S. Chadha, *Appl. Biochem. Biotechnol.*, 2020, **191**, 463–481.
14 K. Chen, X. Zhao, P. Zhang, L. Long and S. Ding, *Biotechnol Biofuels Bioprod*, 2024, **17**, 30.
